# The *Litsea* genome and the evolution of the laurel family

**DOI:** 10.1038/s41467-020-15493-5

**Published:** 2020-04-03

**Authors:** Yi-Cun Chen, Zhen Li, Yun-Xiao Zhao, Ming Gao, Jie-Yu Wang, Ke-Wei Liu, Xue Wang, Li-Wen Wu, Yu-Lian Jiao, Zi-Long Xu, Wen-Guang He, Qi-Yan Zhang, Chieh-Kai Liang, Yu-Yun Hsiao, Di-Yang Zhang, Si-Ren Lan, Laiqiang Huang, Wei Xu, Wen-Chieh Tsai, Zhong-Jian Liu, Yves Van de Peer, Yang-Dong Wang

**Affiliations:** 1 State Key Laboratory of Tree Genetics and Breeding, Chinese Academy of Forestry, Beijing, 100091 China; 2 Research Institute of Subtropical Forestry, Chinese Academy of Forestry, Hangzhou, 311400 China; 3 Department of Plant Biotechnology and Bioinformatics, Ghent University, 9052 Ghent, Belgium; 4 VIB Center for Plant Systems Biology, VIB, 9052 Ghent, Belgium; 5 Key Laboratory of National Forestry and Grassland Administration for Orchid Conservation and Utilization at College of Landscape Architecture, Fujian Agriculture and Forestry University, Fuzhou, 350002 China; 6 College of Forestry and Landscape Architecture, South China Agricultural University, Guangzhou, 510642 China; 7 School of Life Sciences, Tsinghua University, Beijing, 100084 China; 8 Center for Biotechnology and Biomedicine, Shenzhen Key Laboratory of Gene and Antibody Therapy, State Key Laboratory of Chemical Oncogenomics, State Key Laboratory of Health Sciences and Technology (prep), Tsinghua Shenzhen International Graduate School, Tsinghua University, Shenzhen, Guangdong 518055 China; 9 Center for Precision Medicine and Healthcare, Tsinghua-Berkeley Shenzhen Institute (TBSI), Shenzhen, Guangdong 518055 China; 10 Department of Life Sciences, National Cheng Kung University, Tainan, 701 Taiwan; 11 Orchid Research and Development Center, National Cheng Kung University, Tainan, 701 Taiwan; 12 Novogene Bioinformatics Institute, Beijing, 100083 China; 13 Institute of Tropical Plant Sciences, National Cheng Kung University, Tainan, 701 Taiwan; 14 Henry Fok College of Biology and Agriculture, Shaoguan University, Shaoguan, 512005 China; 15 Center for Microbial Ecology and Genomics, Department of Biochemistry, Genetics and Microbiology, University of Pretoria, Pretoria, 0028 South Africa; 16 College of Horticulture, Nanjing Agricultural University, Nanjing, 210095 China

**Keywords:** Evolutionary genetics, Genome evolution, Genome duplication

## Abstract

The laurel family within the Magnoliids has attracted attentions owing to its scents, variable inflorescences, and controversial phylogenetic position. Here, we present a chromosome-level assembly of the *Litsea cubeba* genome, together with low-coverage genomic and transcriptomic data for many other Lauraceae. Phylogenomic analyses show phylogenetic discordance at the position of Magnoliids, suggesting incomplete lineage sorting during the divergence of monocots, eudicots, and Magnoliids. An ancient whole-genome duplication (WGD) event occurred just before the divergence of Laurales and Magnoliales; subsequently, independent WGDs occurred almost simultaneously in the three Lauralean lineages. The phylogenetic relationships within Lauraceae correspond to the divergence of inflorescences, as evidenced by the phylogeny of *FUWA*, a conserved gene involved in determining panicle architecture in Lauraceae. Monoterpene synthases responsible for production of specific volatile compounds in Lauraceae are functionally verified. Our work sheds light on the evolution of the Lauraceae, the genetic basis for floral evolution and specific scents.

## Abstract (web summary)

*Litsea cubeba* belongs to the Lauraceae family within the Magnoliids clade. Here, the authors assemble its genome and reveal divergence of inflorescence and sexual differentiation, the phylogenetic relationships across the Lauraceae and related species, and biosynthetic genes related to essential oil synthesis.

## Introduction

Lauraceae, also referred to as the laurel family, is a family from the order Laurales in Magnoliids^1^. The family includes a total of 2500–3000 globally distributed species in 44 genera of the woody subfamily Lauroideae and about 25 species in 1 genus of the parasitic subfamily Cassythoideae^2^. The morphological features of flowers in Lauraceae species^3^, including various inflorescences and the existence of both bisexual and unisexual flowers^4–6^ (Supplementary Fig. 1), provide a reference for studying flower evolution in angiosperms. In addition, the specific scents from Lauraceae species have made the laurel family economically important as a source of medicine, spices, and perfumes^2^. A diverse array of terpenoids, mainly monoterpenes and sesquiterpenes, defines the scents of different species in Lauraceae^7,8^. Terpene synthases (TPSs) have been primarily responsible for the monoterpene production^9^; however, research on the TPS gene families in Lauraceae is still in its infancy due to the hitherto limited genomic data. From a phylogenetic perspective, the relationships among Magnoliids, monocots, and eudicots still remain to be debated^10–13^. For instance, the analysis of the *Cinnamomum kanehirae*^12^ genome supported a sister relationship of Magnoliids and eudicots to the exclusion of monocots, while the genomes of *Liriodendron chinense*^11^ and *Persea americana*^14^ suggested Magnoliids as a sister group to the clade consisting of both eudicots and monocots. Still some other studies, amongst those based on organellar genes and a limited number of nuclear genes, support a sister relationship between Magnoliids and monocots, to the exclusion of eudicots^15,16^. The often-conflicting evolutionary relationships also reflect the morphological complexities among monocots, eudicots, and Magnoliids. For example, the spiral floral phyllotaxis is present in Magnoliids and eudicots, but not in monocots; and Magnoliids and eudicots generally have carpels with one, two, or more ovules, while most monocots have more than two ovules^17^. However, flowers are trimerous in Magnoliids and monocots but tetramerous or pentamerous in eudicots. Intermediate ascidiate carpels are predominantly present in Magnoliids, which differentiates them from other angiosperm lineages^17^.

As two species, *C. kanehirae* and *P. americana*, from the core Lauraceae (including the Laureae-*Cinnamomeae* group and *Persea* group)^18^ have already been sequenced^12,14^, we here present a chromosome-level assembly of the genome of May Chang tree (*Litsea cubeba* Lour.), which is from the sister clade to *C. kanehirae* in the core Lauraceae. It is an important species for producing essential oils (roughly 95% terpenoid) that are widely used in perfumes, cosmetics, and medicine all over the world^19–21^. Further, to revisit the phylogenetic position of Magnoliids relative to eudicots and monocots^11–13^ and to study the evolutionary relationships within the Lauraceae, we sequence the genomes of 47 species of 20 genera in Lauraceae at a low coverage. Also, to uncover the molecular basis for the various floral features and the biosynthesis of scents in Lauraceae, we analyze mixed-tissue and flower bud transcriptomes for 23 species of 16 genera in the Lauraceae family, following by further functional verifications using transient overexpression and enzyme activity assay.

## Results

### Genome sequencing and annotation

*L. cubeba* has a diploid genome (2*n* = 24) (Supplementary Fig. 2a) with an estimated haploid genome size of 1370.14 Mb (Supplementary Fig. 2b). The genome was initially assembled with 155.64× sequencing reads from the PacBio platform. The assembled contigs were subsequently linked into 1514 scaffolds with 10× genomics barcoded reads (Supplementary Note 1 and Supplementary Table 1). We obtained an initial genome assembly with 1514 scaffolds covering 1325.69 Mb, with a contig N50 value of 607.34 kb (Supplementary Table 2). Further scaffolding was done based on 292.17 Gb reads from a sequencing library of Genome-wide Chromosome Conformation Capture (Hi-C). We were able to anchor a total of 1018 scaffolds covering 1253.47 Mb (94.56%) of the assembled genome into 12 pseudochromosomes (Supplementary Figs. 2c, 3, and Supplementary Table 3). To confirm the completeness of the assembly, we performed CEGMA^22^, BUSCO^23^ assessments, and used mRNA sequences of *L. cubeba* and found the completeness of the genome to be 95.97% (Supplementary Table 4), 88.4% (Supplementary Table 5), and 97% (Supplementary Table 6), respectively. A combination of homolog-based comparisons and structure-based analyses resulted in an annotation of 735 Mb transposable elements (TEs), representing 55.47% of the *L. cubeba* genome (Supplementary Table 7), which is between that of *C. kanehirae* (~48% in a 730.7 Mb genome)^12^ and *L. chinense* (~62% in a 1742.4 Mb genome)^11^ (Supplementary Table 8). Long terminal repeats (LTRs) are the predominant TEs in the genome of *L. cubeba*, which represent 47.64% (631 Mb) of the whole genome. Both *L. cubeba* and *L. chinense* have a larger genome size than that of *C. kanehireae*^12^, and they both contain higher content of LTR/gypsy and copia elements (45.31%) than that of the *C. kanehireae* genome (16.50%)^12^. Hence, it suggests that LTR/gypsy and copia elements contribute most to the expansions of the *L. cubeba* and *L. chinense* genomes (Supplementary Table 8).

A high-confidence set of 31,329 protein-coding genes were predicted in the *L. cubeba* genome, of which 29,262 (93.4%) and 27,753 (88.59%) were supported by transcriptome data and protein homologs, respectively (Supplementary Fig. 4a and Supplementary Table 9). A total of 29,651 (94.6%) predicted protein-coding genes were functionally annotated (Supplementary Fig. 4a and Supplementary Table 10) and 30,314 (91.3%) of the genes could be located on the 12 pseudochromosomes. In addition, 1284 (89.2%) of the 1440 protein-coding genes in the BUSCO plant set were predicted in the *L. cubeba* genome (Supplementary Table 5).

We then compared the genomes of 26 plant species to obtain gene families that are significantly expanded in Lauraceae or that are unique to Lauraceae (Supplementary Fig. 4b, c and Supplementary Table 11). Kyoto Encyclopedia of Genes and Genomes (KEGG) and Gene Ontology (GO) enrichment analyses found that the significantly expanded gene families are especially enriched in the KEGG pathways of monoterpenoid biosynthesis, biosynthesis of secondary metabolites, and metabolic (Supplementary Table 12) and in the GO terms of TPS activity, transferase activity, and catalytic activity (Supplementary Table 13). Many monoterpene synthase (TPS-b) genes are included in the above enriched KEGG pathways and GO terms, in line with the roles of TPS-b in the biosynthesis of specific scents (mainly monoterpene) in Lauraceae^12,24^. The enrichment analyses showed that the 711 unique Lauraceae gene families are specifically enriched in the KEGG pathways of plant hormone signal transduction and circadian rhythm—plant (Supplementary Table 14) and in the GO terms of regulation of cellular metabolic and organic cyclic compound metabolic processes (Supplementary Table 15). Hormone-related transcriptional factors are over-presented in the unique gene families to Lauraceae, for example, ABSCISIC ACID-INSENSITIVE 5 (ABI5)^25^ and ethylene-responsive transcription factor ERF098. Furthermore, the species-specific gene families of *L. cubeba* are significantly enriched in the KEGG pathways of biosynthesis of terpenoids and steroids and nitrogen metabolism, and the gene families under significant expansion in *L. cubeba* include many members from the ABC transporter C family, which is generally involved in the membrane transport of the secondary metabolism^26^ (Supplementary Tables 16–21). It is interesting to notice that the TPS and ABC transporter members form gene clusters on chromosomes 8 and 12 (Supplementary Fig. 5).

### The phylogenetic position of Magnoliids among angiosperm

**Fig. 1:**
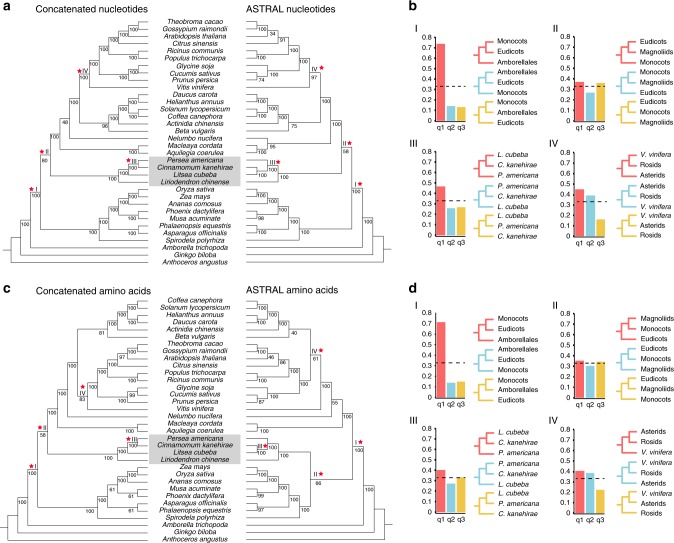
Concatenated- and ASTRAL-based phylogenetic trees. **a** Phylogenetic trees based on the concatenated (left) and multi-species coalescent (MSC) methods (right) using nucleotide sequences. Magnoliids are indicated with a gray background. Red stars with labels I, II, III, and IV refer to the discussions on phylogenetic discordances (see text). **b** Estimated proportions of the 160 single-copy gene trees with different topologies based on nucleotide alignments. The *x*-axis labels q1, q2, and q3 refer to the quartet support for the main topology (red), the first alternative (blue), and the second alternative (yellow), respectively. The dashed line refers to a proportion of 0.33. **c** Phylogenetic trees based on the concatenated (left) and MSC methods (right) using amino acid sequences. Interpretation is as in **a**. **d** Estimated proportions of the 160 single-copy gene trees based on amino acid sequences. Interpretation is same as **b**. Source data underlying (**a**) and (**c**) are provided as a Source Data file.

Laurales are an order of Magnoliids, whose evolutionary position, mainly with respect to eudicots and monocots, is still the object of contention^10–13^. On the basis of the 160 single-copy gene families derived from 19 eudicots, 8 monocots, 4 Magnoliids, and 3 outgroup species (*Amborella trichopoda*, *Ginkgo biloba*, and *Anthoceros punctatus*), we constructed phylogenetic trees from the concatenated sequence alignments of both nucleotide and amino acids sequences (Fig. 1a, c, left side). In these analyses, Magnoliids were found as a sister group to eudicots after their common ancestor diverged from monocots, which agrees with a previous study using the *C. kanehirae* genome^12^. To reduce the possibility of long branch attraction in our phylogenetic analysis, we conducted another phylogenomic analysis without Gramineae species and obtained the same topology (Supplementary Figs. 6 and 7).

Because incomplete lineage sorting (ILS) may play a role in confounding resolution of early-diverging branches within angiosperms, such as the divergence of Magnoliids, eudicots, and monocots, we further conducted the multi-species coalescent (MSC)-based phylogenomic analyses using ASTRAL^27^ by considering each gene tree from the 160 single-copy gene families separately (Fig. 1a, c, right side). The MSC-based phylogeny using nucleotides (Fig. 1a, right side) again supports a sister group relationship between Magnoliids and eudicots, to the exclusion of monocots. However, the MSC-based tree using amino acids (Fig. 1c, right side) suggests Magnoliids to form a sister group with monocots, after their divergence from eudicots. To evaluate the discordance of gene trees in our single-copy gene data set, we used the *Q* value in ASTRAL to display the percentages of gene trees in support of the main topology (q1), and the first (q2) and second (q3) alternative topologies^27^ (Fig. 1b, d). For example, the majority of gene trees inferred by both nucleotide and amino acid sequences support Amborellales being a sister group to the other angiosperms as the main topology (q1), while there are few gene trees that support either monocots (q2) or eudicots (q3) being the sister group to other angiosperms, respectively (I in Fig. 1b, d). In contrast, the branching order for Magnoliids, monocots, and eudicots displays a high level of discordance among the single-copy gene trees, with two nearly equally supported (and one slightly less supported) topologies in both nucleotide and amino acid sequences-based analyses (II in Fig. 1b-II, d-II).

Other discordances among the single-copy gene trees analyzed with ASTRAL concerned the phylogenetic position of *Vitis vinifera*^28^ and the phylogenetic position of *P. americana* (left side in Fig. 1b, d). All phylogenomic analyses focusing on Lauraceae species support a sister relationship of *Litsea* and *Cinnamomum* (right side in Fig. 1b, d).

### Whole-genome duplications in Laurales

**Fig. 2:**
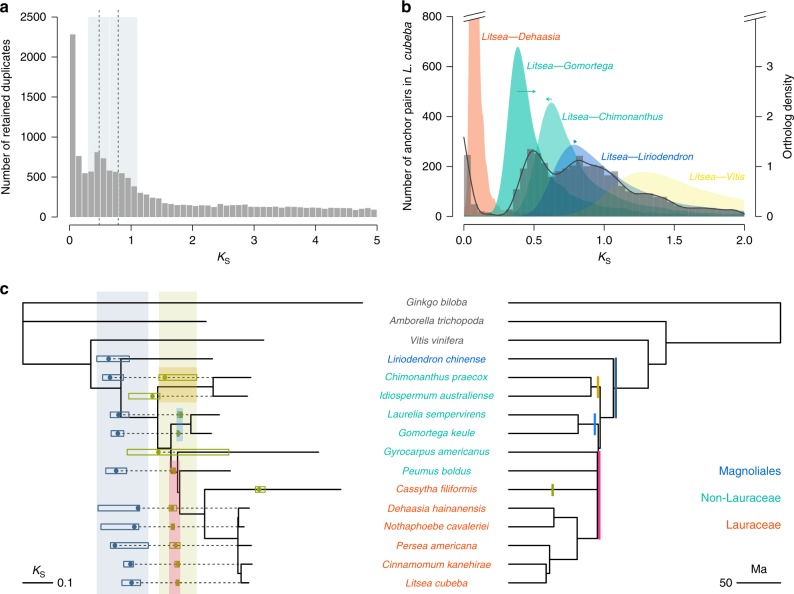
Whole-genome duplications in Laurales. **a** *K*_*S*_ age distribution for the whole paranome of *L. cubeba*. Two *K*_*S*_ peaks are shown by dotted lines at *K*_S_ ≈ 0.5 and 0.8 falling in two *K*_*S*_ ranges highlighted by two gray rectangles in the background from 0.3 to 0.645 and from 0.645 to 1.1, respectively. **b** *K*_*S*_ age distributions for anchor pairs of *L. cubeba* (dark gray histogram and line; peaks represent WGD events) and for one-to-one orthologs between *L. cubeba* and selected Lauralean species and *V. vinifera* (colored filled curves of kernel-density estimates; a peak represents a species divergence event). The arrows in different colors indicate under- (to the left) and overestimations (to the right) of the divergence events and point to the *K*_*S*_ values after corrections of different substitution rates in the three comparisons based on that in *L. cubeba* (see Methods). **c** The phylogeny of Laurales and Magnoliales with branch lengths in *K*_*S*_ units (left) and in absolute divergence time (right). The tree topology and absolute divergence time were retrieved. The *K*_*S*_ ages and their 95% confidence intervals (CIs) for WGDs identified from the whole paranome (Supplementary Fig. 9) are shown in dots and rectangles, respectively, on the left phylogeny. Without considering species with only one *K*_*S*_ peak in their *K*_*S*_ distributions of paralogs, the blue and green rectangles highlight the ranges of 95% CIs of WGDs for the older peaks and the younger peaks, respectively. The red, light blue, and yellow rectangles show the ranges of 95% CIs for three independent WGD events in different lineages of Laurales. Correspondingly, the red, light blue, and yellow bars illustrate the independent WGD events on the right phylogenetic tree with absolute divergence time. The three WGD events occurred at about the same time of the radiation of Lauralean species within ~3 million years. In addition, the green bar denotes a lineage-specific WGD event in *C. filiformis* and the blue bar denotes the WGD event before the divergence of Laurales and Magnoliales.

Genome collinearity and paralog age distributions all show indications of two ancient whole-genome duplication (WGD) events for *L. cubeba*. Intragenomic analysis of gene order reveals collinear regions with up to five (but mostly two to four) paralogous segments (Supplementary Table 22 and Supplementary Fig. 8), while age distributions of synonymous substitutions per synonymous site (*K*_S_) for all paralogous genes (paranome), as well as duplicates retained in collinear regions (anchor pairs) both show two signature peaks for WGD events with a recent peak at *K*_S_ ≈ 0.5 and a more ancient peak at *K*_S_ ≈ 0.8 (Fig. 2a, b). Similarly, previously sequenced genomes (of *C. kanehirae*^12^ and *P. americana*^14^) and transcriptomes (from this study and 1KP^29^) of Lauraceae also show two signature peaks in their paranome *K*_*S*_ distributions, except for *Cassytha filiformis* (Supplementary Fig. 9), for which only one signature peak could be identified. WGD analyses using the *C. kanehirae* genome suggested that the recent WGD in *C. kanehirae* is shared by all the Lauraceae species except *C. filiformis*, while the ancient WGD seems shared by Laurales and Magnoliales, i.e., the two clades that form a sister group in the Magnoliid clade^12^. Also, the analysis of the genome of *L. chinense*, another species in the order of Magnoliales, supported a WGD prior to the divergence between Laurales and Magnoliales^8^.

Our analyses of the transcriptomes of non-Lauraceae Lauralean species, however, found that although some of these species have only one WGD peak in their paranome *K*_*S*_ distributions, others, such as *Peumus boldus*, *Laurelia sempervirens*, *Gomortega keule*, and *Chimonanthus praecox*, have two such peaks (Supplementary Fig. 9). Specifically, compared with those in *L. cubeba*, the *K*_*S*_ values of the two WGD peaks are larger in *C. praecox* and smaller in the other three species, suggesting that the signature peaks in these species may result from either different WGD events or from various substitution rates for duplicates retained following the same WGD events. Indeed, the *K*_*S*_ distributions of one-to-one orthologs identified between *V. vinifera* and species from Laurales and Magnoliales show different *K*_*S*_ peaks for the divergence event between *V. vinifera* and Magnoliids, indicating different substitution rates within Laurales (Supplementary Fig. 10). In short, our results provide evidence of two WGD events not only in the Lauraceae species but also in some non-Lauraceae species in the Laurales order.

To better position the two WGD events identified in the *L. cubeba* genome in the lineage of Laurales, we compared the anchor-pair *K*_*S*_ distribution of *L. cubeba* with the orthologous *K*_*S*_ distributions (Fig. 2b): (1) between *L. cubeba* and *Dehaasia hainanensis*, *G. keule*, and *C. praecox* to represent the divergence of different lineages in Laurales (Fig. 2c); (2) between *L. cubeba* and *L. chinense* to represent the divergence between Laurales and Magnoliales; and (3) between *L. cubeba* and *V. vinifera* to represent the divergence between Magnoliids and eudicots. Both the analysis of *K*_*S*_ distributions and relative rate tests to correct for different substitution rates in different species of Laurales (Supplementary Note 2) suggest that the ancient *L. cubeba* WGD has occurred shortly before the divergence of Laurales and Magnoliales, while the recent WGD has occurred before the divergence of Lauraceae but closely following the divergence of the lineage including *C. praecox* and the lineage including *G. keule* (Fig. 2b, c). Some species, such as *L. sempervirens*, *G. keule*, and *C. praecox*, have not experienced the recent WGD identified in *L. cubeba*, but also show two signature peaks for WGDs in their paranome *K*_*S*_ distributions (Supplementary Fig. 9). Considering *K*_*S*_ peak values (Supplementary Note 2), we infer independent WGDs in three different lineages of Laurales: one in the lineage leading to *C. praecox* and *Idiospermum australiense*; one in the lineage leading to *L. sempervirens* and *G. keule*; and another in the lineage, including Lauraceae, *P. boldus*, and possibly *G. americanus* (Fig. 2c). Interestingly, *C. filiformis*, an obligate parasitic plant in the Lauraceae, and the one with the highest substitution rate in our analysis (Supplementary Fig. 10), show a *K*_*S*_ peak that represents a lineage-specific WGD event after its divergence from other Lauraceae species (Supplementary Fig. 9 and Fig. 2c). However, we propose that *C. filiformis* shares the same WGD history as *L. cubeba* and the other Lauraceae, but draws a different picture because of its accelerated substitution rate responsible for diminishing the signature peaks for the two WGDs in its paranome *K*_S_ distribution.

Lauralean species must have experienced rapid radiation over ~3 million years^30^. Interestingly, the younger WGD peaks in Laurales seem to coincide with such a period (the right-hand tree in Fig. 2c), which could imply that these WGD events might even have facilitated the rapid radiation of the early Lauralean species. On the other hand, it cannot be ruled that there has been only one WGD event that has occurred shortly before the rapid radiation of Laurales. Under such scenario, our observation of three independent WGDs could be explained by one single WGD that has occurred just before the divergence of Laurales followed by independent diploidizations in the three Lauralean lineages during species radiation, with similarity to the process described in the “lineage-specific ohnologue resolution” model^31^.

### The evolution of floral structures in Lauraceae

**Fig. 3:**
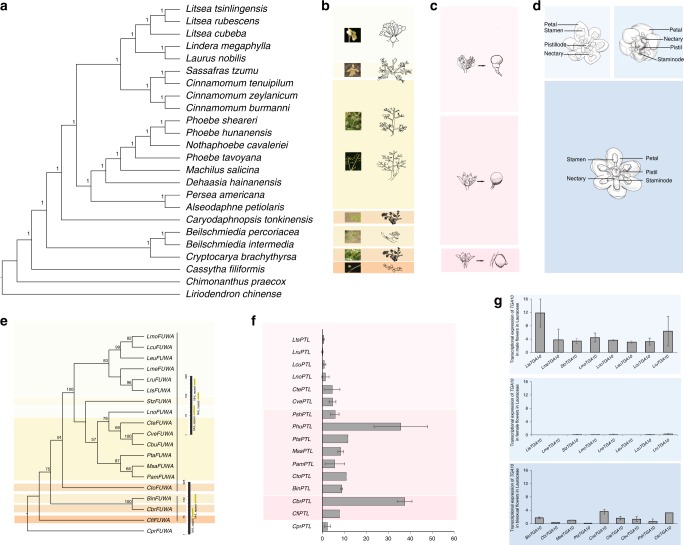
The evolution of floral structures in Lauraceae. **a** Phylogeny of Lauraceae based on a concatenated sequence alignment of 275 single-copy gene families for 22 species in the Lauraceae. **b** The variable panicles in Lauraceae (from bottom to up): spikes in *Cassytha*, spikelike panicles in the *Cryptocarya* group, cymoses panicles in the *Alseodaphne*-*Phoebe* clade and *Cinnamomum*, pseudo-umbel in *Sassafras*, and umbels in the *Laurus*-*Litsea* clade. **c** Perianth tube turbinate or suburceolate present in *Cryptocarya* group and *Cassytha*. *Caryodaphnopsis* and *Alseodaphne*-*Phoebe* clade appear broadly conical and short perianth tube. Perianth tubes are campanulate, short to nearly absent in the *Cinnamomum*-*Litsea* clade. **d** The *Cinnamomum*-*Litsea* clade has unisexual flowers and the other species in Lauraceae have bio-sexual flowers. **e** The phylogenetic tree of FUWA homologs in different Lauraceae species. **f** *PTL* expression in the flower buds of Lauraceae species. The *PTL* expression level was noted as being consistent with the variation of perianth morphology in Lauraceae. *PTL* exhibited a higher level of expression in the flower buds of the basic group lineage (*Cryptocarya* group), which presented an abscission of the perianth tube from the perianth tube encapsulated in fruits. *PTL* had a lower level of expression in the *Litsea*-*Cinnamomum* clade, where the fruit receptacle developed from the perianth tube. **g** TGACG motif-binding protein family member *TGA10* has higher transcriptional expression level in male flowers than that in female flowers from eight unisexual species representing four genera, and *TGA10* also has higher expression lever in male flowers comparing with that in bisexual flowers from nine bisexual species representing six genera of Lauraceae. Source data underlying **a**, **e**–**g** are provided as a Source Data file.

To investigate the evolution of floral structures in Lauraceae, we first inferred the phylogenetic relationships within Lauraceae using both the concatenated and MSC approaches based on single-copy genes identified from the transcriptomes of 22 species representing 16 genera (Fig. 3a, Supplementary Notes 3 and 4, Supplementary Fig. 11, and Supplementary Table 23). We also obtained a plastid phylogeny based on the reconstructed plastid genomes from 27 species representing 19 genera in Lauraceae (Supplementary Note 5 Supplementary Tables 24 and 25). Phylogenetic trees reconstructed from concatenated sequence alignments had similar topologies than the MSC trees, except for the position of *Lindera* and *Laurus* (Supplementary Fig. 12). Comparing the nuclear and plastid trees, however, we identified notable differences for some genera in Lauraceae, such as *Lindera*, *Laurus*, *Nothaphoebe*, *Phoebe*, *Dehaasia*, *Persea*, and *Alseodaphne* (Supplementary Note 6). ASTRAL analysis also shows phylogenetic discordance among gene trees for the discordant nodes between the nuclear tree and plastid tree (Supplementary Fig. 12), indicating a complicated evolutionary history of Lauraceae. Specifically, *Cryptocarya* is the sister group to other Lauraceae species in the plastid tree, while in both the concatenated and MSC trees based on nuclear genes, *Cassytha* is a sister to other Lauraceae species. Similar differences have been reported in previous studies^32–34^, at least in our ASTRAL analysis; strong support is given to the sister relationship between *Cassytha* and other Lauraceae species with few discordant gene trees with respect to *Cassytha* (Supplementary Note 6, Supplementary Fig. 13).

Generally, the inflorescences in Lauraceae are panicles, spikes, racemes, pseudo-umbels, and umbels^2^. These characteristics of inflorescences are of importance to the classification of the Lauraceae family (Fig. 3b). To investigate the genes that are potentially involved in the evolution of inflorescences, we considered the transcriptomes of flower buds for 21 different species (Supplementary Note 7 and Supplementary Table 26) and phylogeny of Lauraceae inferred by MrBayes^35^ were conducted (Fig. 3a). In particular, we focused on the *FUWA* genes in 13 genera, because *FUWA* has been reported to play an essential role in determining panicle architecture in rice, sorghum, and maize^36^. A phylogenetic tree based on the orthologs of FUWA in different Lauraceae species (Supplementary Note 8) seems consistent with the evolution of inflorescences (Fig. 3a, b, and e). The *Cassytha* and *Cryptocarya* group, which diverged earlier than other Lauraceae lineages, have spike and spikelike panicles, respectively, which are usually referred to as irregular panicles. Subsequently, regular panicles and umbels are present in other Lauraceae species. For example, cymose panicles appear in the *Alseodaphne*-*Phoebe* clade, pseudo-umbels appear in *Sassafras*, and umbels appear in the *Litsea*-*Laurus* clade. Consistently, the analyses of domain architectures of the *FUWA* gene indicate that the gene contains two conserved NHL domains in the early-diverging lineages of the *Cryptocarya* Group and *Cassytha* and three NHL domains in other Lauraceae species (Fig. 3e). A similar pattern has also been observed in Lauraceae for involucres. In Lauraceae, genera with panicles and racemes have no involucre, while Sassafras with pseudo-umbels has bracts linear to filamentous, and the *Litsea*-*Laurus* clade with umbels has an obvious involucre. The inflorescences morphological differentiation could be related to the geographic distribution of Lauraceae. The *Cryptocarya* Group and *Cassytha* are found in the Southern Hemisphere, while the other clades are mainly distributed in the amphi-Pacific or Asian areas.

The evolution of perianth tubes in Lauraceae also seems to mirror the phylogeny of Lauraceae (Fig. 3a, c). It has been known that the loss of the trihelix transcription factor PETAL LOSS (PTL) could induce the disruption of perianth development in *Arabidopsis*^37^. Therefore, the differences in *PTL* expression in Lauraceae could be consistent with the variations of perianth tubes. Indeed, comparing with the *Litsea*-*Cinnamomum* clade, where the perianth tubes are indistinct, short, and campanulate, *PTL* genes from other Lauracea clades exhibit higher level of expression in the flower buds and these species have perianth tubes turbinate or suburceolate (Supplementary Note 9 and Fig. 3c, f).

The most recent common ancestor of Laurales was a tree with actinomorphic and bisexual flowers^38^. Extant Lauraceae species include both bisexual (dioecious) and unisexual (monoecious) species (Fig. 3d and Supplementary Fig. 1). To identify the genes involved in the sexual determination in Lauraceae, we produced and integrated Illumina transcriptome data for flower buds from 17 species in 10 genera of Lauraceae, including 8 unisexual species in 4 genera and 9 bisexual species in 6 genera (Supplementary Note 7 and Supplementary Table 26). The comparative analyses of the transcriptome data illustrate that the differentially expressed genes (DEGs) between unisexual and bisexual species are enriched in the KEGG pathway of plant hormone signal transduction. Among these genes, *TGA10* shows obviously higher expression in male flowers from monoecious species than both female flowers from monoecious species and bisexual flowers from dioecious species in Lauraceae (see “Methods,” Supplementary Note 10 and Fig. 3d, g). Our results hence suggest that *TGA10* is involved in male flower development, which is consistent with studies that have found that *TGA10* is required for anther development^39^. Moreover, a hypothesized protein (Lcu01G_02292) may also have contributed to sexual determination in Lauraceae, considering their differential expression patterns in bisexual and unisexual flowers in Lauraceae (Supplementary Fig. 14). We also compared the MADS-box genes in the sequenced Lauraceae species and a few other angiosperms (Supplementary Note 11, Supplementary Fig. 15, and Supplementary Table 27). Notably, we found that *SOC1*-like genes are expanded in both *L. cubeba* (seven members of *SOC1*) (Supplementary Table 28) and *C. kanehirae* (eight members of *SOC1*)^12^. Consistent with the expanded *SOC1* clade, the *SVP* clade is also expanded and it counts five members in *L. cubeba* (Supplementary Fig. 15). It has been reported that the interaction of *SOC1* and *AGL24* from the SVP clade integrates flowering signals in *Arabidopsis*^40^. Both the expanded *SOC1* and *SVP* clades could be involved in complex flowering regulation networks and could relate to differential regulation of dioecious plant flowering.

### Mono-TPS involved in volatiles production in Lauraceae

**Fig. 4:**
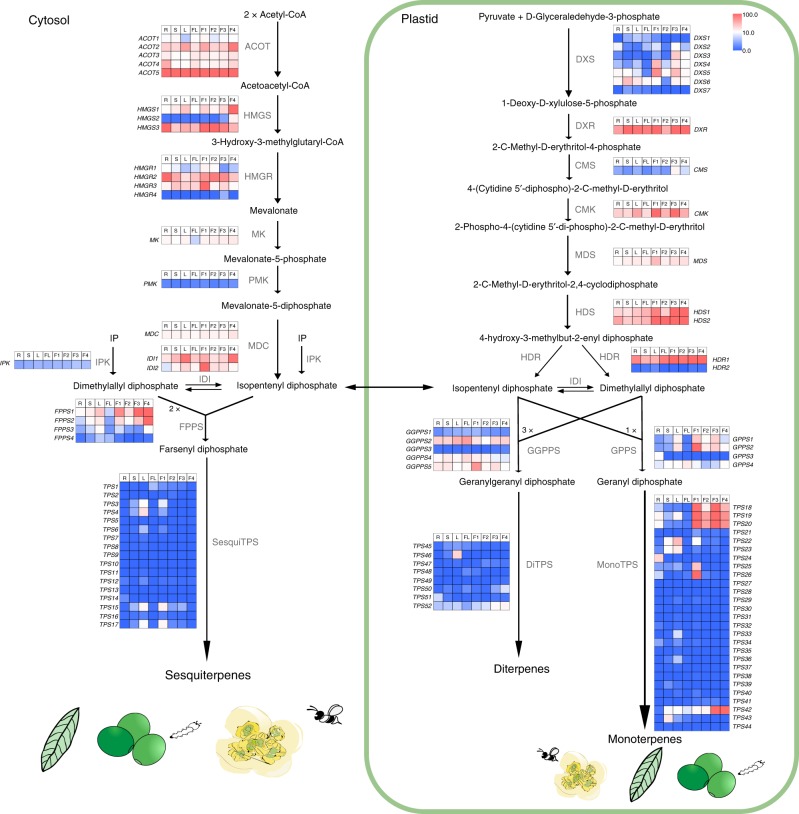
Scent biosynthesis in Lauraceae. Tissue-specific relative expression profiles (red–blue scale) of genes implicated in terpenoid biosynthesis (heat map). Intermediates are shown in black, and the enzymes (Supplementary Table 34) involved at each step are shown in gray. The genes involved in the MEP pathway exhibit a high level of fruit-development-specific expression, which may contribute to the biosynthesis of large amounts of monoterpenes. SesquiTPSs, or the responsible sesquiterpene biosynthesis of flowers, involve the gene expansion of 17 members (full amino acid length >200 aa). MonoTPSs involved in the production of monoterpenes in fruits also show signs of family expansion for 27 members (full amino acid length >200 aa) of *L. cubeba*. *LcuTPSs* form a gene cluster in chromosome 8 (Supplementary Fig. 5). MVA pathway mevalonate pathway, MEP pathway mevalonate-independent (deoxyxylulose phosphate) pathway, R root, S stem, L leaves, FL flower, F1 fruit 40 days after full bloom, F2 fruit 70 days after full bloom, F3 fruit 100 days after full bloom, F4 fruit 140 days after full bloom. Source data are provided as a Source Data file.

The essential oils produced by Lauraceae are widely used commercially, and contain a variety of components (Supplementary Table 29), such as geranial, neral, limonene, and linalool^7,12,19,20^. TPSs are the rate-limiting enzymes in the production of such terpenoids^9,41^ (Fig. 4). The present gene family analysis suggests that the TPS-b gene clade is significantly expanded in Lauraceae (Supplementary Tables 12 and 13). We hence identified all the TPS genes in Lauraceae by combining the data for the *L. cubeba* genome and the transcriptome data for 23 species, from 16 genera, in the Lauraceae family (Supplementary Tables 30 and 31). Lauraceae species with a high percentage content of essential oil had larger numbers of TPS-b members (Supplementary Tables 30 and 31), for example, *L. cubeba* possessed 24 TPS-b genes in (3–7%, the percentage of essential oil in fresh fruit), *Cinnamomum verum* possessed 12 TPS-b genes (1.32–2.13%, the percentage of essential oil in fresh leaves), *Machilus salicina* possessed 13 TPS-b genes (1.05%, the percentage of essential oil in fresh leaves), and *P. americana* possessed 17 TPS-b genes (1%, the percentage of essential oil in fresh ripe fruit)^14^ (Supplementary Tables 30 and 31).

We analyzed the first key enzyme in the scent biosynthetic pathway, namely 1-deoxyxylulose 5-phosphate synthase (DXS), which has three clades (Supplementary Fig. 16). The members in the clade B are expanded across *Litsea*, *Beilschmiedia*, and *Sassafras* (Supplementary Note 12 and Supplementary Fig. 16); as a result, Lauraceae produce high levels of terpenoids. *LcuDXS3-5* belongs to clade B and exhibits very high expression in fruits according to the transcriptome data^42^ (Fig. 4). Furthermore, transient overexpression of *LcuDXS3* in *L. cubeba* could induce the increase of several components of monoterpene and sesquiterpene (Supplementary Note 12 and Supplementary Fig. 17).

**Fig. 5:**
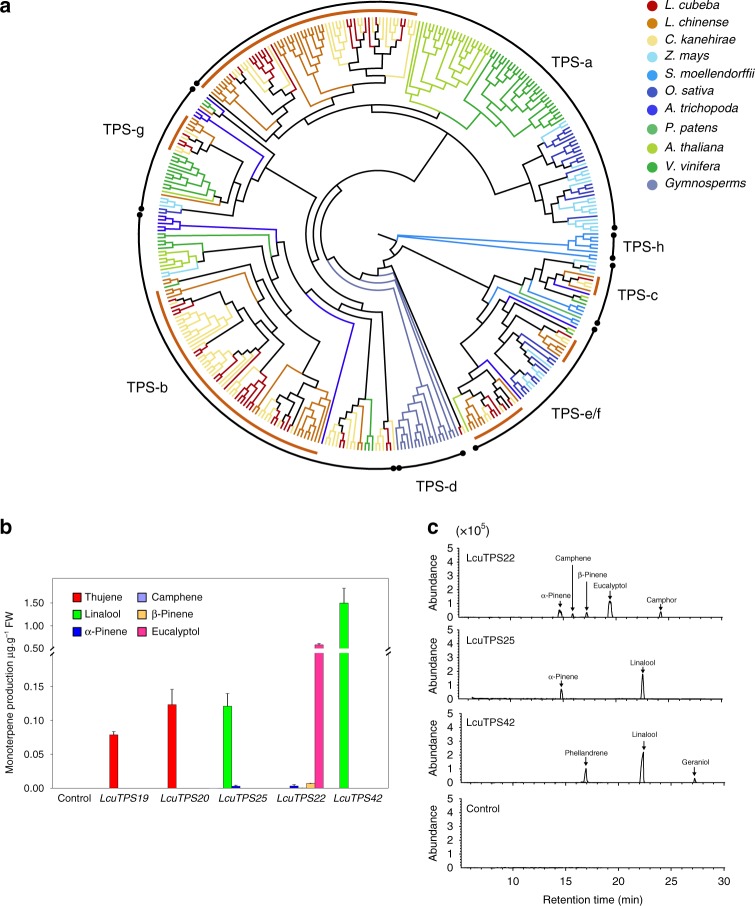
Phylogeny and functional verification of LcuTPSs. **a** Phylogeny of TPSs. Putative full-length TPS proteins (>200 amino acids in length) identified in *L. cubeba* (Supplementary Table 32) and 10 other sequenced plant genomes (Supplementary Table 33) were subjected to phylogenetic analysis. TPS subfamilies are shown along the circumference of the circle. **b** Transient overexpression of *LcuTPSs* in *Nicotiana benthamiana* leaves. After infiltration, the plants were grown for 2 days and the presence or concentration of the monoterpenes was detected using GC-MS analysis. Data represent the mean ± SDs of three biological replicates. **c** Identification of enzymatic products after incubating recombinant LcuTPSs proteins with geranyl diphosphate. The recombined enzyme expressed in *Escherichia coli* was purified by Ni^2+^ affinity. The volatile terpenes were further analyzed by GC-MS analysis comparison with authentic standards (Supplementary Fig. 18). Source data underling **a**, **b** are provided as a Source Data file.

To further investigate the members of TPS-b and TPS-g involved in terpenoid biosynthesis, additional expression and functional verification studies were conducted in *L. cubeba*. Among the 52 full-length TPS genes in *L. cubeba*, 27 were predicted as monoterpene synthase genes, 17 as sesquiterpene synthase genes, and the remaining 8 as diterpene synthase genes (Fig. 4 and Supplementary Tables 32–34). Tandem duplication (Fig. 5a and Supplementary Fig. 5) events have contributed to the expansion of the TPS-b subfamilies. Both Illumina transcriptome sequencing data (Fig. 4) and qRT-PCR verification showed (Supplementary Fig. 17) that *LcuTPS22* specifically accumulated in leaves. Transient overexpression and enzyme activity assay both demonstrated that LcuTPS22 catalyzed the accumulation of α-pinene, β-pinene, eucalyptol, camphene, eucalyptol, and camphor, which are the main volatile components of the leaves of Lauraceae species (Fig. 5). In addition, *LcuTPS18*, *19*, *20*, *25*, *26*, and *42* were all highly expressed in the *L. cubeba* fruits (Fig. 4 and Supplementary Fig. 17). Transient overexpression and enzyme activity assay also indicated that LcuTPS42 catalyzed the biosynthesis of linalool, phellandrene, and geraniol, which are the main components of the specific scents in Lauraceae (Fig. 5 and Supplementary Fig. 18).

Besides the TPS-b gene families, other genes may also function as mediators in scent biosynthesis in *L. cubeba*. For example, plant hormone signal transduction enrichment of gene families is unique to Lauraceae; therefore, we investigated the endogenous hormone content and found that abscisic acid (ABA) had a unique peak close to that of the biosynthesized monoterpene in *L. cubeba* (Supplementary Fig. 19a). Correspondingly, the treatment of *L. cubeba* leaves with ABA induced an increase in both the level of monoterpene (Supplementary Fig. 19b) and the expression of *LcuTPS22* (Supplementary Fig. 19c). In summary, our results provide insights into the candidate genes involved in scent production in the Lauraceae family. Further studies are, however, required to elucidate the mechanisms underlying the regulation of these genes in the scent biosynthetic pathways in Lauraceae.

## Discussion

It seemed that the evolutionary relationships between Magnoliids, monocots, and eudicots as still unresolved, since our ASTRAL analyses suggest the possible ILS during the rapid divergence of early mesangiosperms^29^. ILS is a result of polymorphic alleles in the ancestral populations. Despite nucleotide polymorphisms, prevalent copy number variations could also exist in the ancestral populations and exacerbate the effects of ILS, as suggested by gene count data used to infer the phylogenetic position of Magnoliids in a previous study on the *P. americana* genome^14^. Besides sequence data, approaches based on synteny data could perhaps provide additional evidence with respect to the evolutionary relationship of Magnoliids. For example, utilizing sequence dissimilarities of orthologous genes located on orthologous synteny blocks, synteny-based phylogenetic analyses for *P. americana* supported Magnoliids as the sister group to monocots and eudicots^14^. Recent analyses by some of us^43^, considering phylogenies based on the relative gene order using synteny network data, rather than gene sequence similarities, suggested a sister group relationship between Magnoliids and monocots, thereby supporting the MSC (ASTRAL) tree based on amino acid sequence alignments (Fig. 1c).

The results of the present analyses of the key enzymes involved in monoterpene biosynthesis pathway would hence suggest that the duplications of *DXS* and *TPS* gene families may have led to the separation of biological features in terpenoid production^9,44^, and gene overexpression may enhance the production of main components in terpene (Fig. 5). Therefore, we propose that gene family sizes and regulation of gene expression both contribute to the accumulation of terpenoid in *L. cubeba*. As reported in a recent publication, following expansion of the TPS gene family, gene cluster formation, gene functional differentiation, and gene regulation divergence all could contribute to the variations in terpene production and concentration among individual species^45^. In addition, new functions gained by recently duplicated TPS genes may be correlated with recently evolved terpene compounds that are capable of defending plants against biotic and abiotic stresses^46^.

In summary, the *L. cubeba* genome provides a valuable resource for elucidating the Lauraceae evolution toolkit. Most of the present phylogenetic analyses suggest a sister group relationship of Magnoliids and eudicots, after the divergence of their common ancestor from monocots. However, the exact evolutionary relationships between Magnoliids, monocots, and dicots remain to be solved because topological conflict suggests substantial ILS in the (short) branches separating these three groups. Phylogenetic inference also suggests that the obligatory parasitic species *Cassytha* is sister to the other Lauraceae. In the *L. cubeba* genome, remnants of two ancient WGD events could be detected. In addition, comparative and functional analyses showed that TPS-b genes were significantly expanded and responsible for terpenoid biosynthesis in Lauraceae. In conclusion, our data offer insight into the genetic diversity and evolution of Laurales—and the scents they produce—and provide a stronger understanding of the evolution and diversification of Lauraceae.

## Methods

### Sample preparation and sequencing

For genome sequencing, we collected tissues from *L. cubeba* in Zhejiang Province (Supplementary Note 1), China, with a karyotype of 2*n* = 24 and with uniform and small chromosomes (Supplementary Fig. 2a). Genomic DNA was isolated from the buds of *L. cubeba*, and at least 10 μg of sheared DNA was taken. SMRTbell template preparation involved DNA concentration, damage repair, end repair, ligation of hairpin adapters, and template purification, undertaken using AMPure PB Magnetic Beads (Pacific Biosciences). Finally, we carried out 20 kb single-molecule real-time DNA sequencing using PacBio to sequence a DNA library on the PacBio Sequel platform, yielding ~213 Gb PacBio data (read quality ≥0.80 and mean read length ≥7 kb) (Supplementary Table 1).

### Genome assembly and assessment

The assembly of *L. cubeba* genome was conducted using PacBio and 10× Genomics Linked-Reads. De novo assembly of the PacBio reads was performed using FALCON (https://github.com/PacificBiosciences/FALCON/) with single-molecule, real-time sequencing. Briefly, the longest 60× coverage of subreads were selected as seeds to do error correction with parameters “--output_multi --min_idt 0.70 --min_cov 4 -- max_n_read 300.” The corrected reads were then aligned to each other to construct string graphs with parameters “--length_cutoff_pr 11000.” The graph was further flited with parameters “--max_diff 70 --max_cov 70 --min_cov 3” and contigs were finally generated according to these graphs. The primary contigs were then polished using Quiver by aligning SMRT reads. The total length of this assembly was 1700.7 Mb, with a contig N50 of 460.7 kb. The total length of this assembly was significantly longer than the estimated genome size (1370.1 Mb), which indicated that several redundant sequences were presented in the assembly. This was confirmed by the large proportion of BUSCO duplicated genes, with 21.3% of the BUSCO genes duplicated in the assembly. We, therefore, undertook a redundancy filtering step for the assembly. The removal of genome hybrids was achieved by a purge of haplotigs, which provided a pipeline to the reassignment of allelic contigs^47^. Briefly, the pipeline first identified putative heterozygous contigs through read-depth analysis. Contigs with a high proportion of bases within the 0.5× read-depth peak were assigned as putative heterozygous contigs. These putative heterozygous contigs were then subject to a sequence alignment to identify its allelic companion contig. Then the identified haplotigs were removed from the assembly iteratively. We attempted several cutoffs for the pipeline, then selected a genome version that was balanced between redundancy and integrity. The total length of the filtered genome assembly was 1315.8 Mb, with a contig N50 of 603.7 kb. The filtered assembly was then connected by 10× genomics data. We used the BWA-ME algorithm (Toward better understanding of artifacts in variant calling from high-coverage samples) to align the 143.28 Gb 10× genomics data (Supplementary Table 1) by the default setting and the scaffolding was performed by FragScaff (A hybrid approach for de novo human genome sequence assembly and phasing) using the alignment file as the input. We selected on the parameters of *j* = 1.25 and *u* = 2 to achieve a relatively conservative assembly and minimize the introduction of scaffolding errors. After that, we used PBjelly (Mind the gap: upgrading genomes with PacificBiosciences RS long-read sequencing technology) software to fill gaps with PacBio data. The options were -minMatch 8 -sdpTupleSize 8 -minPctIdentity 75 -bestn 1 -nCandidates 10 -maxScore −500 -nproc 13 -noSplitSubreads. In order to get enough corrected genome sequences, we used Pilon (v1.18) with default settings to do the second round of error correction. For the input BAM file, we used BWA (Aligning sequence reads, clone sequences and assembly contigs with BWA-MEM) to align total 359.77 Gb Illumina short reads (Supplementary Table 1) to the assembly and used SAMTOOLS (The Sequence Alignment/Map format and SAMtools) to sort and index the BAM file. Finally, we obtained a genome with a contig N50 size of 607.34 kb and a scaffold N50 size of 1.76 Mb. The total length of this genome version was 1325.59 Mb, and it contained 0.90% Ns (Supplementary Table 2).

To confirm the quality of the genome assembly, we performed a BUSCO (http://busco.ezlab.org/) assessment using 1440 single-copy orthologous genes (Supplementary Table 5), and we found a genome completeness value of 88.4%. To confirm the high coverage of the assembly, we mapped available mRNA sequences to the assembled genome using BLAST software (http://genome.ucsc.edu/goldenpath/help/blatSpec.html). In total, 32,499 (97.91%) were supported by transcriptome data, with a sequence coverage >50% (Supplementary Table 6).

### Hi-C library construction and assembly of the chromosome

For Hi-C libraries construction, 3 g seedlings were crosslinked with 40 ml 2% formaldehyde solution at room temperature for 30 min in a vacuum. Glycine (2.5 M) was added to quench the crosslinking reaction. After fixation, 0.5 g of fixed tissue was ground with liquid nitrogen for the first round of library preparation. The extracted nuclei were resuspended with 50 μl 0.5% SDS followed by incubation at 62 °C for 10 min, and then SDS molecules were quenched by adding 25 μl 10% Triton X-100 and incubated at 37 °C for 20 min. For following restriction digestion in intact nuclei, DNA was digested with the four-cutter restriction enzyme DpnII and incubated at 37 °C overnight. The DpnII enzyme was inactivated at 62 °C for 20 min. The cohesive ends were filled and marked with biotin-labeled dCTP and dCAP, dTTP, and dGTP by Klenow and incubated at 37 °C for 30 min. The proximal chromatin DNA ligation was conducted using T4 DNA ligation enzyme at room temperature for 4 h. After centrifugation at 2500× *g* for 5 min, the reaction mixture was resuspended in SDS buffer (50 mM Tris-HCl, 1% SDS, 10 mM EDTA, pH 8.0), proteinase K was added, and the mixture was incubated at 55 °C for 30 min. The formaldehyde crosslinking of the nuclear complexes was reversed by the addition of 30 μl of 5 M NaCl and incubation at 65 °C overnight. For subsequent chromatin, DNA was purified and fragmented by sonication on a Covaris sonicator. After DNA repair and 3′ A addition, adaptor was added. The amplification of library molecules was performed according to the standard Illumina library preparation protocol. The libraries were sequenced on HiSeq X Ten DNA sequencers to obtain paired-end 150-nucleotide reads, following the manufacturer instructions (Illumina). The libraries were sequenced on HiSeq X Ten DNA sequencers to obtain paired-end 150-nucleotide reads, following the manufacturer instructions (Illumina). Two libraries were produced in this study, and each library produced about 400 million Hi-C reads. The high-quality reads were mapped to the draft scaffolds using a fast and accurate short-read alignment using a Burrows–Wheeler transform^48^, and then the duplicated mapping reads and unmapped reads were removed using SAMtools (Sequence Alignment/Map format and SAMtools)^49^. The separations of HiC read pairs mapped in draft scaffolds were analyzed using chromosome-scale scaffolding of de novo genome assemblies based on chromatin interactions (LACHESIS) to produce a likelihood model for genomic distance between read pairs, and the model was used to identify putative misjoins. The greater the number of reads of interaction between two contigs, the greater the likelihood of a class. Contig clustering was done according to the number of interactions reads. Then, HiC data were used to do scaffolding using LACHESIS software, and finally about 94.56% sequences were grouped into 12 super scaffolds (Supplementary Figs. 2c and 3). The contigs were sorted according to the intensity of every two contig interactions and the mapping location of interaction reads. The data on the clustering of the chromosomes and the assembly of *L. cubeba* are given in Supplementary Table 3.

### Transposable elements and repetitive DNA

TEs contribute to genome dynamism both in size and in structure, through insertion and eventual loss^50^. Genomic scaffolds were masked using RepeatMasker (http://www.repeatmasker.org) on the default settings after employing RepeatModeler/RepeatScout/Piler/LTR_finder software with RepBase database prediction. Meanwhile, the TEs in the genome were identified through Repeat ProteinMask soft annotation, using the RepBase database.

### Predictions of genes and noncoding RNA

To generate gene models with high confidence, we applied a gene-annotation framework by combining evidence drawn from spliced transcripts of RNA-seq ab initio gene predictors and protein evidence drawn from orthologous proteins of closely related and model plant species. The detailed procedure was referred to Supplementary Note 13 and finally 31,329 protein-coding genes were predicted (Supplementary Table 9). We then applied the functional assignments of protein-coding sequences of *L. cubeba* genes to the public protein databases KEGG (http://www.genome.jp/kegg/), SwissProt (http://www.uniprot.org/), TrEMBL (http://www.uniprot.org/), and InterProScan v5.11-51.0 (https://www.ebi.ac.uk/interpro/). In this way, we generated functional assignments for 94.6% (29,651/31,329) of the available *L. cubeba* genome (Supplementary Table 10).

Noncoding RNA was determined using structural features and homology assignments. rRNA was determined via BLAST to rRNA sequencing of other species, using the high levels of conservation in species. tRNA was identified using tRNAscn-SE (http://lowelab.ucsc.edu/tRNAscan-SE/). In addition, other types of noncoding RNA, including miRNA and snRNA, were identified using INFERNAL to search the Pfam database (http://infernal.janelia.org/).

### Ortholog detection with OrthoMCL

We downloaded genome and annotation data for *Actinidia chinensis* (GCA_003024255.1), *Anthoceros angustus (not published)*, *Aquilegia coerulea* (GCA_002738505.1), *Ananas comosus* (GCF_001540865.1), *Arabidopsis thaliana* (TAIR 10), *Asparagus officinalis* (GCF_001876935.1), *A. trichopoda* (V1.0), *Beta vulgaris* (V1.2.2), *C. kanehirae* (GCA_003546025.1), *Citrus sinensis* (GCF_000317415.1), *Coffea canephora* (V1.0), *Cucumis sativus* (GCF_000004075.2), *Daucus carota* (v2.0), *G. biloba* (2019-06-04), *Gossypium raimondii* (v2.1), *Glycine soja* (v1.1)^51^, *Helianthus annuus* (GCF_002127325.1), *L. chinense* (GCA_003013855.2)*, Musa acuminate* (V1.0), *Macleaya cordata* (GCA_002174775.1), *Nelumbo nucifera* (GCF_000365185.1), *Nymphaea colorata* (GCA_902499525.1), *Oryza sativa* (v7.0), *P. americana* (v2.0), *Phoenix dactylifera* (GCF_000413155.1), *Phalaenopsis equestris* (APLD00000000.1), *Prunus persica* (V2.1), *Populus trichocarpa* (V3), *Solanum lycopersicum* (SL2.50), *Ricinus communis* (GCF_000151685.1), *Spirodela polyrhiza* (GCA_001981405.1), *Theobroma cacao*(V1.1), *V. vinifera* (V12X), and *Zea mays* (v.2.1). We removed genes with open-reading frames of <200 bp and performed gene family clustering using OrthoMCL.

### Gene family expansion and contraction

We measured the expansion and contraction of orthologous gene families using the software CAFÉ 4.2 (https://github.com/hahnlab/CAFE)^52^. On the basis of the maximum likelihood modeling of gene gain and loss, we analyzed gene families for signs of expansion or contraction (Supplementary Fig. 4c) using genome data from 26 species (Supplementary Table 11). Our KEGG enrichment analyses were conducted for unique, significantly expanded, and constructed gene families in Magnoliids, Lauraceae, and *L. cubeba*.

### Phylogenetic reconstruction

In order to identify more phylogeny-informative sites, we screened the genome data of 26 species for common conserved gene families (Supplementary Table 11) by including the single-copy genes in at least 22 species, and two copies in the remaining 4 species. Thus, the total number for a single gene family was no more than 30. In species with two copies of a given gene family, we selected the gene with the best BLAST hits. Finally, we obtained 1201 common conserved gene families in 26 species. For the gene families that had undergone expansion and contraction and the divergent time estimation, we constructed a phylogenetic tree based on these 1201 common conserved gene families using MrBayes software with GTR+*Γ* model^35^.

For phylogeny reconstruction in angiosperms, we derived 160 common single-copy gene families from BUSCO database for 34 species (Fig. 1); the phylogenetic tree was constructed based on concatenated single-copy gene family alignment and coalescent-based approaches that incorporate individual gene tree. A concatenated phylogenomic tree (Fig. 1) was constructed using MrBayes with GTR+*Γ* Model^35^. For coalescent-based approaches, we used ASTRAL to combine gene trees from 160 single-copy genes, and the *q* value was used to account for variation among gene trees owing to ILS. For gene tree estimation, both the nucleotides and amino acids were used to reconstruct the phylogenetic trees by RAxML v.8.1.17^53^. GTRGAMMA and GAMMAJTT were set as estimation models for the nucleotide and amino acid tree, respectively.

The multi-locus bootstrapping and the built-in local posterior probabilities of ASTRAL were used to estimate branch support^27^ and to test for polytomies^54^. This estimation, which was conducted with the built-in functionality of ASTRAL (version 4.11.2) by finding the average number of gene tree quartets defined around the branch (Fig. 1), resulted in percentage of gene trees that agreed with each branch in the species tree. We use “ASTRAL topology” to refer to the tree inferred from 160 unbinned amino acid alignments in which branches with 33% or less support is contracted, which refers to the potential ILS.

Phylogenomic analysis in Lauraceae was conducted using both nuclear and plastid genome data. To conduct phylogeny analysis of Lauraceae using single-copy gene families, we generated and integrated Illumina-sequenced transcriptomic data for various tissues (including flower buds, flowers, leaves, stems, buds, and bark) of 23 species representing 16 genera, representing the main lineage of Lauraceae and *C. praecox* (Supplementary Note 3 and Supplementary Table 23) and the Pacbio Iso-seq data for *C. filiformis* and *C. praecox* (Supplementary Note 4). We developed a phylogenetic tree through a concatenated sequence alignment of 275 single-copy gene families as the phylogeny of the angiosperm (Fig. 3a). We also used ASTRAL5.6.3 to reconstruct the species tree based on a data set similar to the concatenated tree (275 single-copy tree), and the ILS was also identified by −q 8 argument. The 1st and 2nd codon positions and 3rd codon position were derived from the same data set of the species tree, and used to reconstruct the phylogenetic trees based on a similar method. To perform a phylogenetic analysis using plastid genome data, we assembled the plastid genomes from incorporated re-sequenced data of 27 species representing 19 genera of Lauraceae as well as the outgroup *C. praecox* (Supplementary Note 5 and Supplementary Table 24).

### Identification of whole-genome duplications in Laurales

*K*_*S*_-based age distributions for all paralogous genes (paranome) of genomes and transcriptomes in Magnoliids were constructed. Concisely, the paranome was built by identifying gene families with the mclblastline pipeline (v10-201) (micans.org/mcl)^55^ after performing all-against-all BLASTP search with an *E* value cutoff of 1 × 10^−10^. Each gene family was aligned using MUSCLE (v3.8.31)^56^. Then the CODEML program in the PAML package (v4.4c)^57^ was used to estimate *K*_*S*_ for all pairwise comparisons within a gene family. Gene families were further subdivided into subfamilies for which *K*_*S*_ values between members did not exceed 5. As a gene family of *n* members produces *n*(*n* − 1)/2 pairwise *K*_*S*_ estimates for *n* − 1 retained duplication event, we corrected for the redundancy of *K*_*S*_ values by first inferring a phylogenetic tree for each subfamily using PhyML^58^ with the default settings. Then, for each duplication node in the resulting phylogenetic tree, all *m K*_*S*_ estimates for a duplication between the two child clades were added to the *K*_*S*_ distribution with a weight of 1/*m*, so that the sum of the weights of all *K*_*S*_ estimates for a single duplication event was 1.

To identify synteny or collinear segments in the genome of *L. cubeba*, i-ADHoRe (v3.0) was used with the parameters level_2_only=FALSE, enabling the ability to detect highly degenerated collinear segments resulting from more ancient large-scale duplications (this is achieved by recursively building genomic profiles based on relatively recent collinear segments)^59^. The *K*_*S*_ distribution of paralogs located on collinear segments (anchor pairs) was calculated using maximum likelihood in the CODEML program of the PAML package (v4.4c)^57^.

The *K*_*S*_-based ortholog age distributions were constructed by identifying one-to-one orthologs between species by selecting reciprocal best hits^60^, followed by *K*_*S*_ estimation using the CODEML program, as above. To compare different substitution rates in Magnoliids species, we compared the *K*_*S*_ distribution of one-to-one orthologs identified between *V. vinifera* and *L. cubeba* and the *K*_*S*_ distributions of one-to-one orthologs identified between *V. vinifera* and *C. kanehirae*, *P. americana*, *D. hainanensis*, *Nothaphoebe cavaleriei*, *C. filiformis*, *P. boldus*, *G. americanus*, *L. sempervirens*, *G. keule*, *C. praecox*, *I. australiense*, and *L. chinense* (Supplementary Fig. 9). Because *V. vinifera* and Magnoliids diverged at a specific time, we would expect similar peaks in orthologous *K*_*S*_ distributions if all Magnoliid species had similar substitution rates.

To circumscribe the placements of the WGDs identified in the genome of *L. cubeba* in the phylogeny of Magnoliids, we compared the anchor-pair *K*_*S*_ distribution of *L. cubeba* and the orthologous *K*_*S*_ distributions between *L. cubeba* and *D. hainanensis*, *G. keule*, *C. filiformis*, *C. praecox*, *L. chinense*, and *V. vinifera*. To quantify the differences in substitution rates among these Magnoliids species, we performed a relative rate test, using *V. vinifera* as an outgroup to calculate *K*_*S*_ distances after the divergence between *L. cubeba* and each of the Magnoliids species. The *K*_*S*_ distance between any two species in a relative rate test was estimated by the mode of their orthologous *K*_*S*_ distribution. As the substitution rates seemed to vary considerably among the sequenced Magnoliids so far (Supplementary Fig. 8), the calculated *K*_*S*_ distance for *L. cubeba* in each relative rate test was used to correct the orthologous *K*_*S*_ peaks between *L. cubeba* and other Magnoliids species under the assumption that the two species have an identical substitution rate after their divergence (arrows in Fig. 2b). For example, using the *K*_*S*_ distance between *L. cubeba* and *G. keule*, the *K*_*S*_ distance between *V. vinifera* and *G. keule*, and the *K*_*S*_ distance between *V. vinifera* and *L. cubeba*, we used a relative rate test to calculate *K*_*S*_ distances to the lineage of *G. keule* and *L. cubeba* after their divergence, respectively. Then, orthologous *K*_*S*_ between *L. cubeba* and *G. keule* was corrected by twice of the *K*_*S*_ distance to *L. cubeba* (assuming that *L. cubeba* and *G. keule* had the same substitution rate).

To further place the WGD peaks identified in the paranome *K*_*S*_ distributions from other genomes and transcriptomes in Magnoliids (Supplementary Fig. 8), we built gene families with a collection of species in Laurales and *L. chinense*, along with *A. trichopoda* and *G. biloba* as extra outgroups (Fig. 2d), using OrthoMCL on the default settings^61^. Among the identified gene families, we selected 23 single-copy gene families to estimate the branch lengths in the *K*_*S*_ unit using PAML (v4.4c)^57^ with the free-ratio model. The topology and absolute divergence times of the species tree were retrieved from TimeTree^30^. To infer the ages of WGDs in the *K*_*S*_ unit as well, *K*_*S*_ peaks were identified in a paranome *K*_*S*_ distribution by an R function from github.com/stas-g/findPeaks after a smooth spline was fitted to the *K*_*S*_ distribution. To obtain a 95% CI for each identified *K*_*S*_ peak, *K*_*S*_ values of paralogs in a wide range of the estimated peak were resampled 100 times to obtain 100 bootstrapped peaks. To map all the identified *K*_*S*_ peaks and their 95% CIs onto the species phylogeny in the *K*_*S*_ unit, we divided *K*_*S*_ values of the identified peaks and the 95% CIs by two, with the assumption that duplicate genes evolved at similar substitution rates after WGD events. We then considered each tip in the species phylogeny as a starting point and mapped half of the *K*_*S*_ value of each peak from the tip toward the root of the phylogeny to date when WGD events occurred in the phylogeny (Fig. 2d).

### Low-coverage genome sequencing and plastid genome assembly

Low-coverage genome sequence data were generated for 47 species, including a 15× strategy for species in *Litsea* and a 30× strategy for species in other genera in Lauraceae (Supplementary Tables 24 and 25). The plastid genome data were de novo assembled. We also downloaded the complete plastid genomes of Lauraceae species from the NCBI and combined them into a single database. Then, BLAST was used to search against our plastid database, and the blast-hit pair reads were corresponding to the plastid origin, which accounted for about 3% for all species in this study. Then the PLATANUS^62^ software was used to assemble the picked reads. After the contig was assembled, the scaffold tool from PLATANUS was used to scaffold the first assembly version based on the same paired-end reads to obtain the second assembly. Next, gap-closing was performed using the PLATANUS assembler to close the gap in the second assembly and obtain the final assembly, which contained at least three scaffolds, representing LSC, SSCm, and IRs. The scaffold parts were then annotated in DOGMA (http://dogma.ccbb.utexas.edu/), and artificially assembled into the almost complete plastid genome, with reference to the published plastid genome of Lauraceae. To identify the conservative segments for phylogenetic reconstruction, we used a HomBlocks pipeline^63^ to locate the collinear regions for alignment. The plastid genomes were used to construct a phylogenetic tree (Fig. 3).

### Transcriptomic data and analysis in Lauraceae

For library construction, a total of 1.5 µg RNA was prepared, and libraries were generated using the NEBNext^®^ Ultra™ RNA Library Prep Kit for Illumina^®^ (NEB, USA). The index codes were put into attribute sequences for each sample. The clustering of the index-coded samples was carried out on a cBot Cluster Generation System using the TruSeq PE Cluster Kit v3-cBot-HS (Illumina). Subsequently, the libraries were sequenced on an Illumina HiSeq platform to produce paired-end reads. The raw reads were further processed using in-house Perl scripts to remove reads containing adapters, reads containing ploy-N, and low-quality reads. At the same time, Q20, Q30, and GC-content levels and sequence duplication levels of the clean data were calculated. All downstream analyses were conducted with high-quality clean data. Transcriptome assembly was accomplished using Trinity^64^, with min_kmer_cov set to 2 as a default and with all other parameters set to default values. All transcriptome assemblies were further valued by a BUSCO assessment (https://busco.ezlab.org/)^23^ (Supplementary Fig. 20). Protein sequences and coding sequences of the transcripts were predicted using TransDecoder (http://transdecoder.github.io). For genes with more than one transcript, the longest was taken as the unigene and gene expression levels were estimated by RSEM^65^. Gene (or transcript isoform) expression values were provided using the fragments per kilobase per million mapped reads method^66^. To determine whether the chimeric transcripts occurred in the gene family of TPSs, we conducted a local BLAST (*E* value <1 × 10^−6^) and found that 18 transcripts from the de novo assembled transcriptome of *L. cubeba* were corresponded to the genome data (Supplementary Table 35).

We generated three sets of transcriptome data. First, we obtained Illumina-sequenced transcriptomic data for various *L. cubeba* tissues to enable the *L. cubeba* genome assembly. The transcriptome data were mapped on to the *L. cubeba* genome for gene expression analysis. Second, we generated and integrated Illumina-sequenced transcriptomic data for various tissues (including flower buds, flowers, leaves, stems, buds, and bark) of 23 species in 16 genera, representing the main lineage of Lauraceae and *C. praecox* (Supplementary Note 3 and Supplementary Table 23), and the Pacbio Iso-seq data of *C. filiformis* and *C. praecox* (Supplementary Note 4). These de novo mixed-tissue transcriptome data were used to conduct a phylogenetic analysis of Lauraceae, and were also used to annotate gene homologs, including the *TPSs* and *DXSs* of this family. The gene homologs were identified using the HMMER software package. Third, to explore the genes involved in the regulation of the evolution of inflorescences of Lauraceae, we generated transcriptomic data for flower buds in triplicate for 21 species, representing 13 genera in Lauraceae (Supplementary Note 7 and Supplementary Table 26).

The transcriptomes of flower buds were used to excavate the genes involved in inflorescence adaptation and sexual differentiation in Lauraceae. We screened genes that had been reported to be involved in panicle and perianth development in other species. The phylogenetic trees of the candidate genes in Lauraceae were constructed. Only the phylogenetic tree constructed by the FUWA homologs of Lauraceae was consistent with the evolutionary characteristics of inflorescences in this family. The detailed method for the selection of FUWA in Lauraceae is given in Supplementary Note 8. The expression levels of the gene homologs in Lauraceae were compared and *PTL* was found to have a similar expression pattern to that during the presentation of the abscission of the perianth tube and its encapsulation in fruit (Fig. 3f and Supplementary Note 9). Moreover, the DEGs involved in the development of bisexual and unisexual flower buds of Lauraceae were analyzed based on the flower bud transcriptome data. The DEGs (fold change >2, *P* < 0.05) between the female and male flowers in *Litsea tsinlingensis*, *Litsea rubescens*, *L. cubeba*, *Lindera megaphylla*, and *Sassafras tzumu*, were selected. KEGG pathway and GO term-enrichment analyses of DEGs were subsequently conducted for each species. Interestingly, the DEGs were observed to be significantly enriched in the Plant Hormone Signal Transduction (map04075) in each species. Unexpectedly, TAG10 and a hypothesized protein (Lcu01G_02292 in the region of 124099255-124107806 in chr1 of the *L. cubeba* genome) were included in the enriched plant hormone signal transduction pathway of each of the above species, and exhibited distinctively different expression modes between male and female flower buds. Finally, we analyzed the expression modes of *TAG10* and the hypothetical protein in the transcriptome data of male, female, and bisexual flower buds in Lauraceae. The detailed method for the selection of *PTL*, *FUWA*, and *TGA10* genes in Lauraceae are given in Supplementary Notes 8–10.

### TPSs identification and functional validation experiments

To avoid missing potential TPS genes, candidate TPSs were identified from the predicted proteomes of *L. cubeba* and other species by pfamscan based on the HMMER suite (http://hmmer.janelia.org/), using the Pfam profiles of PF01397 and PF03936 as queries (*E* value < 10^−5^) with a protein length over 200 amino acids^9^. The candidate TPS genes were further inspected manually using InterProScan5 (http://www.ebi.ac.uk/interpro/) to confirm putative full-length TPS genes. A total of 52 such full-length *LcuTPS* genes were identified. Although the above criteria may result in the identification of pseudo- or partial genes, 41 of the 52 identified TPS genes were found to have more than 500 amino acids. The full-length TPSs were analyzed with ChloroP for the prediction of N-terminal plastidial targeting peptides (http://www.cbs.dtu.dk/services/ChloroP/). The analysis of the exon/intron structures of the full-length *TPS* genes were also conducted using GSDS (http://gsds.cbi.pku.edu.cn/), and the conserved motif RR(X)_8_W and DDXXD were labeled (Supplementary Fig. 21). The MG2C (http://mg2c.iask.in/mg2c_v2.0/) software was used to construct gene distribution maps of *L. cubeba* chromosomes. Putative full-length TPSs (>200 amino acids in length) identified in *L. cubeba* and other sequenced plant genomes (Supplementary Tables 32 and 33) and maximum likelihood trees were built using CIPRES (https://www.phylo.org) with the JTT model using 1000 bootstrap replicates.

Gene function was validated in vivo and in vitro. For gene function validation in vivo, endogenous transient overexpression was performed in *L. cubeba* and tobacco (*N. benthamiana*) leaves. The empty vector and constructs containing *LcuTPS19*, *LcuTPS20*, *LcuTPS22*, *LcuTPS25*, and *LcuTPS42* were carried by *Agrobacterium* cultures and infiltrated into the leaves using a 1 mL needleless syringe. After infiltration, the plants were grown for 2 days; then, a leaf near (<5 mm) the infiltration point was collected and immediately frozen in liquid nitrogen. These samples were stored at −80 °C for volatile analysis using GC-MS, with 1 μg of ethyl decanoate added to serve as an internal standard. For GC-MS analysis, the samples were ground and incubated at 40 °C for 30 min. The volatiles were further extracted using SPME fiber with 50/30 μm divinylbenzene/carboxen/polydimethylsiloxane (DVB/CAR/PDMS) (Supelco Co., Bellefonte, PA, USA). GC-MS analysis was conducted on an Agilent 6890N gas chromatograph coupled to a mass spectrometer (Agilent 5975B, Santa Clara, CA, USA) with a fused silica capillary column (DB-5MS) coated with polydimethylsiloxane (19091 S-433) (60 m × 0.25 mm internal diameter, 0.25 μm film thickness). The oven temperature was programmed to start at 50 °C for 2 min, and then ramped to 80 °C at a rate of 3 °C min^−1^, followed by a second ramp to 180 °C at a rate of 5 °C min^−1^, and a third ramp to 230 °C at a rate of 10 °C min^−1^, finally, ramp to 250 °C at a rate of 20 °C min^−1^. The conditions were as follows: ion source, 230 °C; electron energy, 70 eV; GC-MS interface zone, 250 °C, and a scan range of 50–500 m/z. There were three biological replicates for transient overexpression analysis. To identify the target monoterpene, the retention time was compared with that of an authentic standard purchased from Sigma-Aldrich, which was further validated using the NIST Mass Spectral Library. The primers are shown in Supplementary Table 36. The details of the experimental procedure for transient expression analysis in *L. cubeba* and tobacco are given in Supplementary Note 14.

For gene function validation in vitro, the full-length open-reading frames of *LcuTPS22*, *LcuTPS25*, and *LcuTPS42* were cloned and inserted into the pET28a vector, and then transformed into *E. coli* BL21 (DE3) pLysS cells (Transgen, China). Recombinant protein was induced with 0.2 mM isopropyl-β-d-galactopyranoside for 20 h at 16 °C, and the expressed recombinant protein was then purified. In the enzymatic assays, the recombinant protein was incubated with 25 mM HEPES, pH 7.2, 100 mM KCl, 10 mM MgCl_2_, 10% (v/v) glycerol, 5 mM DTT, and 30 μM geranyl diphosphate (GPP, Sigma) in pH 7.2 at 30 °C for 1 h^24^. The volatiles were analyzed using GC-MS analysis. To identify the target monoterpene, the retention time was compared with that of an authentic standard purchased from Sigma-Aldrich, which was further validated using the NIST Mass Spectral Library. There were three biological replicates for the analysis of the enzyme activity. The primers are shown in Supplementary Table 36, and the details of the procedure are given in the Supplementary Note 14.

### Reporting summary

Further information on research design is available in the Nature Research Reporting Summary linked to this article.

## Supplementary information

Supplementary Information

Peer Review

Reporting Summary

## Data Availability

A reporting summary for this article is available as a Supplementary Information file. Data supporting the findings of this work are available within the paper and its Supplementary Information files. The data sets generated and analyzed during the current study are available from the corresponding author upon request. The genome and transcriptome sequences described in this manuscript have been submitted to the National Center for Biotechnology Information (NCBI) under accession codes PRJNA562049 [https://www.ncbi.nlm.nih.gov/bioproject/PRJNA562049] (whole genome and assembly data), PRJNA562115 [https://www.ncbi.nlm.nih.gov/bioproject/PRJNA562115] (transcriptome data of 23 Lauraceae species), and PRJNA562080 [https://www.ncbi.nlm.nih.gov/bioproject/PRJNA562080] (low-coverage genome data of 47 Lauraceae species). The data underlying Figs. 1a, c, 3a, e–g, 4, 5a, b and Supplementary Figs. 6, 7, 13, 14, 15a, 16a, 17b–i, 19b, 19c, as well as Supplementary Tables 30 and 31 are provided as a Source Data file.

## Source data

Source Data
