# Supplementary material for: The Litsea genome and the evolution of the laurel family
Source: Nat Commun. 2020 Apr 3;11:1675. doi: 10.1038/s41467-020-15493-5 (PMC7125107; doi:10.1038/s41467-020-15493-5)
Supplement: Supplementary file 1 — Supplementary Information [file 41467_2020_15493_MOESM1_ESM.pdf]

The *Litsea* genome and the evolution of the laurel family

Chen *et al*

Supplementary Note 1. Sample preparation for *Litsea cubeba* genome sequencing

For genome sequencing, we collected buds of *L. cubeba*. Genomic DNA was extracted using a modified cetyltrimethylammonium bromide (CTAB) protocol. For transcriptome analysis, we collected leaves, flowers, and roots from *L. cubeba* in Zhejiang Province, China, using a karyotype of 2n = 24 (Supplementary Figure 2a).

Genome sizes can be determined from the total number of k-mers, divided by the peak value of the k-mer distribution[1]. To estimate the genome size of *L. cubeba*, we used a 350 bp pair-end library with 93.08 Gb high-quality reads to calculate the distribution of k-mer values, and found the main peak to be 54 (Supplementary Figure 2b). We estimated the *L. cubeba* genome size as 1370.14 Mbp, with a 1% heterozygosity rate and a 70.59% repeat sequence, based on an analysis of k-mer-numbers/depths. We used k-mer 41 to obtain a preliminary assembly of *L. cubeba*, with a scaffold N50 size of 776 bp and a corresponding contig N50 size of 591 bp.

Supplementary Note 2. Whole genome duplication analysis in Laurales

The K_S peaks for WGDs in *L. cubeba* are both younger (smaller K_S values) than the orthologous K_S peak between *L. cubeba* and *V. vinifera*, implying that the two WGD events are specific to Magnoliids. To compare the WGD peaks of *L. cubeba* and the speciation events in the lineage of Magnoliids, we performed relative rate tests and corrected orthologous K_S peaks between *L. cubeba* and other Magnoliids species by assuming that they have the same substitution rate as *L. cubeba* (see Methods and arrows in Fig. 2b). The K_S peak for the ancient WGD in *L. cubeba* has a slightly larger K_S value than the corrected orthologous K_S peak between *L. cubeba* and *L. chinense*, suggesting that the ancient WGD has occurred shortly before the divergence of Laurales and Magnoliales. The K_S peak for the recent WGD in *L. cubeba* seems older than the orthologous K_S peak between *L. cubeba* and *D. hainanensis* or *C. filiformis* (Supplementary Figure 22), but younger than the corrected orthologous K_S peak between *L. cubeba* and *C. praecox*. Furthermore, the recent K_S peak is smaller than but apparently overlaps with the corrected orthologous K_S peak between *L. cubeba* and *G. keule*. These comparisons hence indicate that the recent WGD has occurred before the divergence of

Lauraceae but closely following the divergence events of the lineage including *C. praecox* and the lineage including *G. keule*.

To test whether species such as *L. sempervirens*, *G. keule*, and *C. praecox*, that did not share the recent WGD identified in *L. cubeba*, but also show two signature peaks for WGDs in their paranome K_S distributions (Supplementary Figure 9), have undergone separate lineage-specific WGD events, we used half of the value of a K_S peak and its 95% confidence interval (CI) in a paranome K_S distribution to represent the age of a WGD event. Then, we mapped all the ages of WGD events onto a species phylogeny that has branch lengths in K_S units (see Methods and the left-hand tree in Fig. 2c). For the Lauralean species with two K_S peaks, the 95% CIs of the older K_S peaks all fall around the time of divergence between Laurales and Magnoliales, in line with the occurrence of the ancient WGD just before the divergence of the two lineages (Fig. 2b). The 95% CIs of the younger K_S peaks supported independent WGDs in three different lineages of Laurales (Fig. 2c): one WGD in the lineage leading to *C. praecox* and *Idiospermum australiense*; one WGD in the lineage leading to *L. sempervirens* and *G. keule*; and another in the lineage including Lauraceae, *P. boldus*, and possibly *G. americanus* as suggested by the analyses above (Fig. 2b). For species with one K_S peak, such as *I. australiense* and *G. americanus*, the WGD ages and their 95% CIs are between or overlapping with the 95% CIs for the ancient and recent WGDs from other Lauralean species, suggesting that the two WGD signatures in these two species may have been mixed due to artifacts in transcriptome sequencing and assembly.

Supplementary Note 3. Illumina sequencing of mixed-tissue samples for 23 species

We collected fresh tissue samples (flower buds, flowers, leaves, stems, buds, and bark) from 22 species of Lauraceae and *Chimonanthus praecox*, from late February to early May 2018 (Supplementary Table 23). All fresh tissue samples were collected from trees, and cut into small pieces with diameters of no more than 5 mm, and they were immediately added to the RNAlater™ Stabilization Solution (Invitrogen™, Thermo Fisher) for further RNA extraction. Fresh samples of *Machilus salicina* and *Phoebe tavoyana* were collected from the South China Botanical Garden in Guangzhou in March 2018. The five tissue samples did not include

flowers collected from *Alseodaphne petiolaris* of the South China Botanical Garden in early March. *Phoebe sheareri* and *Phoebe hunanensis* were collected from the Chenshan Botanical Garden, Shanghai. *Beilschmiedia intermedia* were sampled from the Tropics Rainforest of Jianfengling, Hainan in March, and *Beilschmiedia percoriacea* was collected from the Hainan Fengmu Experiment Forest Farm in Tunchang, Hainan. Samples of *Caryodaphnopsis tonkinensis*, *Cinnamomum verum*, *Cinnamomum tenuipile*, *Cinnamomum burmanni* and *Persea americana* were collected from the Xishuangbanna Tropical Botanical Garden. Samples of *Nothaphoebe cavaleriei* were collected from Mount Enmei, Sichuan Province in May 2018. Both female and male individuals of *Sassafras tzumu*, *Litsea rubescens*, and *Lindera megaphylla* were sampled from late February to early March 2018 from Wuling Mountain, Enshi Tujia, and from Miao Autonomous Prefecture, Hubei Province. Samples of *Litsea tsinlingensis* were collected from the Qinling Mountains in Hanzhong, Shanxi Province, in March 2018, and samples of *L. cubeba* were collected from the Fuyang District of Hanghzou, Zhejiang Province. Female and male individuals of *Laurus nobilis* were collected from the Chenshan Botanical Garden, Shanghai. *Cassytha filiformis* (May 29, 2018) was collected from the Phoenix Mountains, Guangzhou in late May 2018. Samples of *C. praecox* were collected from the Fuyang District of Hangzhou, Zhejiang Province in March 2018. For library construction, we mixed RNA with equal quantities (1 μg per tissue) of tissues drawn from the flower buds, flowers, leaves, stems, buds, and bark of each species.

Supplementary Note 4. PacBio transcriptome sequencing for two species

For PacBio library construction, we collected samples of *C. filiformis* and *C. praecox*, including flower buds, leaves, stems and bark. Samples of *C. praecox* were collected from the Fuyang District of Hangzhou, Zhejiang Province. Samples of *C. filiformis* were collected from the Phoenix Mountains of Guangzhou, Guangdong Province. Fresh tissues were picked from plants, cut into small pieces, and then immediately added to RNAlater™ Stabilization Solution (Invitrogen™, Thermo Fisher) for further RNA extraction. Total RNA was extracted using the RNAprep Pure Plant Kit and genomic DNA contaminants were removed using RNase-Free DNase I. It is not easy to extract RNA from tissues of *C. filiformis* and especially from its flower buds, as they become adherent after being digested. We obtained enough total RNA

from buds, leaves, stems, and bark after several rounds of extraction and freeze concentration but failed to extract enough RNA from the flower tissues. Therefore, we constructed a PacBio library from an RNA mixture with equal quantities of flower buds, leaves, stems, and bark of *C. filiformis* (1.02 μg per tissue) and *C. praecox* (3.00 μg per tissue).

Supplementary Note 5. Low-coverage genome sequencing for 47 species

Samples of 47 species (Supplementary Table 24) were collected. Fresh, healthy, and tender leaves, leaf buds, and flower buds were picked and dried using silica gel. Samples were mainly collected from three trees and then, for further sequencing, from another tree. We collected the samples between February 22 and June 20 in aforementioned region in China. Samples of *A. petiolaris*, *M. salicina*, *P. tavoyana*, *Actinodaphne lecomtei*, and *Neocinnamomum delavayi* were collected from the South China Botanical Garden in Guangzhou, China. From the Xishuangbanna Tropical Botanical Garden, Yunnan Province, we collected samples of *P. americana*, *C. tonkinensis*, *C. verum*, *C. tenuipile*, *C. burmanni*, and *Cryptocarya brachythyrsa*. From the Chenshan Botanical Garden in Shanghai, we collected samples of *P. hunanensis*, *P. sheareri*, and *L. nobilis*. From Jianfengling National Forest Park, Hainan Province, China, we collected *Dehaasia hainanensis*, *Syndiclis chinensis*, *B. intermedia*, and *Alseodaphne hainanensis*. Samples of *B. percoriacea* were collected from the Hainan Fengmu Experiment Forest Farm of Tunchang, Hainan Province, China. From the Wuling Mountains, Enshi Tujia, and the Miao Autonomous Prefecture, Hubei Province, China, we collected *S. tzumu*, *L. megaphylla*, and *L. rubescens*. Samples of *L. tsinlingensis* were collected from the Qinling Mountains, Hanzhong, Shanxi Province. *N. cavaleriei* was collected in Mount Enmei, Sichuan Province. *Neolitsea sericea* was sampled from the Institute of Forestry in Zhoushan, Zhejiang Province. Samples of *L. cubeba* and *C. praecox* were collected from the Fuyang District of Hangzhou, Zhejiang Province. *C. filiformis* was collected from the Phoenix Mountains of Guangzhou, Guangdong Province.

Supplementary Note 6. Phylogeny of Lauraceae

To investigate the evolution of Lauraceae, we reconstructed the phylogenetic tree of this family based on representative species from different groups. Both concatenated and MSC approaches

were applied using 275 single-copy genes (Fig. 3a) derived from the transcriptomes of 22 Lauraceae species, *C. praecox* (Calycanthaecae), and the annotated genome of *Liriodendron chinense*. The latter two species were treated as an outgroup based on the previously published phylogenetic tree by 1KP[2] (see Methods, and Supplementary Table 23. Then, the plastid genomes assembled from Illumina data were used to reconstruct the plastid phylogeny (see Methods, Supplementary Note 1.4 and Supplementary Tables 25, 26). The phylogenetic inference by concatenated and MSC methods revealed similar topology among different trees, with the only difference being the systematic position of *L. megaphylla* and *L. nobilis*. A sister relationship between these two species in the concatenated tree was observed, and *L. nobilis* diverged earlier than *L. megaphylla* in the MSC tree. However, these two trees, based on single copy nuclear genes, have several topological discordances compared with the plastid phylogeny in several clades. The *Laurus* was the first diverged taxa in the Laureae clade in plastid trees, which was similar to that observed in the MSC tree. *P. americana* and *A. petiolaris* form a sister clade, which diverged first in the *Persea* clade in the nuclear tree. In the plastid tree, the first divergent species is *D. hainanensis*, following by *P. americana* and *A. petiolaris*. Within the inter nodes of the Persea clade, the relationships between *P. tavoyana*, *N. cavaleriei*, and *M. salicina* also varied between markers. The systematic positions for the early divergent clades in Lauraceae have previously been reported to be variable; these groups included *Caryodaphnopsis* (Supplementary Figure 11, *Cary*. cl.), *Beilschmiedia* plus *Cryptocarya* (Supplementary Figure 11, *Bei*. cl.), and *Cassytha* (Supplementary Figure X, *Cas*. cl.)[3-5]. The present phylogenetic trees reconstructed based on nuclear genes (using concatenated and MSC methods) both supported that *Cassytha* is the first diverged group in Lauraceae, while the first divergent group is *Bei*. clade based on plastid phylogeny. Although topological inconsistency between nuclear and plastid genes was observed, the nodes of the relevant position yielded strong support values, indicating the complex evolutionary history of Lauraceae.

The quartet score, obtained with ASTRAL[6], was used to measure the amount of gene tree conflict around a branch. Then, we used the alternative quartet topologies setting to calculate the quartet score for main topology, the first alternative, and the second alternative

(Supplementary Figure 12). Although we obtained the high support values by Bayesian inference based on concatenated single copy genes, seven conflict signals (q1 $\leq$ 50) were identified in the MSC tree; these ILS events corresponded to the topological heterogenetic nodes between nuclear and plastid trees within the *Litsea* clade, in the ancestors of *Phoebe*, *Machilus*, *Dehaasia*, *Alseodaphne* and *Persea*. In the base group, we identified conflict signal around the early divergent nodes with the exceptions of *Cassytha*, suggesting that conflict in the set of gene trees contributed to the phylogenetic uncertainty for *Caryodaphnopsis* and *Cryptocarua* clades in previous phylogenetic studies. These findings also indicated the complex evolutionary history of this diverse family, and further study is required to explore the underlying reasons.

Interestingly, two methods based on single-copy genes supported the sister relationship between *Cassytha* and other Lauraceae species (also the high quartet score for the main topology), while *Cassytha* was shown to be sister to core Lauraceae and Persea clade in the plastid tree. *Cassytha*, the only parasitic genus in Lauraceae, is a vine-herbal and leafless plant with a short life cycle, and occupies a unique niche in the Lauraceae lineage. It also displays a long branch in both phylogenetic trees (Supplementary Figure 12). Therefore, the special systematic position between nuclear and plastid trees of *Cassytha* might result from the differential substitution rate between nuclear and plastid genes owing to its parasitic lifestyle.

Supplementary Note 7. Illumina transcriptome sequencing of flower buds in 21 species

Samples of flower buds of 21 species representing 12 genera (Supplementary Table 26) were collected from areas in which the species are found. Fresh flower bud tissues were collected from the plants, cut into small pieces, and immediately added to the RNAlater™ Stabilization Solution (Invitrogen™, Thermo Fisher) for further RNA extraction. We collected samples from February 22 to June 20 in China. Samples of *A. petiolaris*, *M. salicina, P. tavoyana, A. lecomtei*, and *N. delavayi* were collected from the South China Botanical Garden of Guangzhou. From the Xishuangbanna Tropical Botanical Garden, Yunnan Province we collected samples of *P. americana*, *C. tonkinensis*, *C. verum*, *C. tenuipile*, *C. burmanni*, and *C. brachythyrsa.* From the Chenshan Botanical Garden of Shanghai we collected samples of *P.*

hunanensis, *P. sheareri*, and *L. nobilis*. From the Tropics Rainforest of Jianfengling, Hainan Province we collected *D. hainanensis*, *S. chinensis*, *B. intermedia*, and *A. hainanensis*. Samples of *B. percoriacea* were collected from the Hainan Fengmu Experiment Forest Farm of Tunchang, Hainan Province. From the Wuling Mountains, Enshi Tujia and Miao Autonomous Prefecture of Hubei Province we collected *S. tzumu*, *L. megaphylla*, *L. rubescens*, *Litsea chunii, Litsea ichangensis*, and *L. elongata.* Samples of *L. tsinlingensis*, and *Litsea pungens* were collected from the Qinling Mountains of Hanzhong, Shanxi Province. *N. cavaleriei*, *Litsea veitchiana, Litsea coreana* var. lanuginosa, *Litsea moupinensis*, *Litsea sericea*, *Litsea moupinensis* var. szechuanica, and *Litsea populifolia* were collected from Mount Enmei, Sichuan Province. *N. sericea* was sampled from the Institute of Forestry in Zhoushan, Zhejiang Province. Samples of *L. cubeba* and *C. praecox* were collected from the Fuyang District of Hangzhou, Zhejiang Province. *C. filiformis* was collected from the Phoenix Mountains of Guangzhou, Guangdong Province. We collected *Litsea garrettii*, *L. rubescens*, *Litsea glutinosa* and *Litsea pierrei* from the Mengla County, Xishuangbanna. Samples of *Litsea mollis* and *Litsea euosma* were collected from Dushan County, Guizhou Province. From the Nonggang National Natural Reserve of Guangxi Province, *Litsea foveolata* and *Litsea dilleniifolia* were collected. Samples of *L. coreana* var. sinensis and *Litsea auriculata* were collected from the Tianmu Mountains in Hangzhou, Zhejiang Province.

Supplementary Note 8. Phylogenetic analysis of *FUWA*

To investigate the molecular basis of inflorescences, we conducted an analysis of the gene using the transcriptome data of the above flower buds of species representing 12 genera in Lauraceae (Supplementary Table 26). An evolutionarily conserved gene, *FUWA*, has been reported to play an essential role in determining panicle architecture in rice, sorghum, and maize[7]. We first conducted a local blast using the transcriptome data of the flower buds, with the *FUWA* gene sequence in rice (The GenBank accession number is KF736096) as an anchor. We found all *FUWA* homologs in the species of the Lauraceae. This includes *FUWA* in *L. euosma* (*FUWA*, Cluster-42021.35595.p1), *L. mollis* (*FUWA*, Cluster-24912.70110.p1), *M. salicina* (*FUWA*, Cluster-14576.92105.p1), *B. intermedia* (*FUWA*, Cluster-41104.4.p1), *C. brachythyrsa* (*FUWA*, Cluster-9924.29390.p1), *C. filiformis* (*FUWA*, Cluster-9564.0.p1), *C.*

tonkinensis (*FUWA*, Cluster-5363.0.p1), *P. americana* (*FUWA*, Cluster-25278.0.p1), *P. tavoyana* (*FUWA*, Cluster-33502.11337.p1), *C. burmanni* (*FUWA*, Cluster-19053.106858.p1), *C. verum* (*FUWA*, Cluster-48080.0.p1), *C. tenuipile* (*FUWA*, Cluster-4431.5014.p1), *S. tzumu* (*FUWA*, Cluster-24588.26985.p1), *L. nobilis* (*FUWA*, Cluster-8800.111830.p1), *L. megaphylla* (*FUWA*, Cluster-34192.82400.p1), *L. cubeba* (*FUWA*, Cluster-29324.38906.p1), *L. rubescens* (*FUWA*, Cluster-19053.106858.p1), *L. tsinlingensis* (*FUWA*, Cluster-44055.195733.p1), and *C. praecox* (*FUWA*, Cluster-39303.0.p1). Subsequently, we constructed a phylogenetic tree using the *FUWA* homologs in Lauraceae and found that this tree was consistent with the evolutionary characteristics of inflorescences in Lauraceae (Fig. 3a, e). To further examine the gene structure of *FUWA* homologs in Lauraceae, we performed blastp for each *FUWA* gene sequence on the NCBI website. The results demonstrated *FUWA* contains the three conserved protein domain and the NHL-like1 domain (cd14953) in most species, except *B. intermedia*, *C. brachythyrsa,* and *C. filiformis*; the three species only showed the two first NHL-like1 domains (Fig. 3e).

Supplementary Note 9. PETAL LOSS gene expression analysis in Lauraceae

PETAL LOSS (PTL), is a regulator of perianth architecture in *Arabidopsis*[8]. To elucidate the potential role of PTL in perianth architecture in Lauraceae, we identified isolated the PTL homologs from the transcriptome data of flower buds in species in Lauraceae using local blast. The expression level of PTL was further analyzed according to the FPKM value (the expected number of fragments per kilobase of transcript sequence per millions base pairs sequenced) using the transcriptome data. For unisexual flowers, including *L. cubeba* (Cluster-7222.0.p1), *L. tsinlingensis* (Cluster-18910.0.p1), *L. rubescens* (Cluster-19053.5683.p1), and *L. nobilis* (Cluster-8800.144294.p1), we took the average value of three replicates for female and male flower buds, respectively. For bisexual flowers, including *C. verum* (Cluster-16029.0.p1), *C. tenuipilum* (Cluster-4431.19175.p1), *P. tavoyana* (Cluster-33502.54075.p1), *P. hunanensis* (Cluster-10881.53903.p1), *P. sheareri* (Cluster-603.87353.p1), *M. salicina* (Cluster-48345.0.p1), *P. americana* (Cluster-21780.14861.p1), *C. tonkinensis* (Cluster-26554.3996.p1), *C. brachythyrsa* (Cluster-9924.21961.p1), *B. intermedia* (Cluster-16338.22.p1), and *C. filiformis* (Cluster-13468.0.p1). We used the three replicates for

flower buds. The results indicated that PTL had a higher expression level in the flower buds of the basic group lineage (*Cryptocarya* group), which indicated an abscission of the perianth tube from the perianth tube encapsulated in fruits. PTL had a lower expression level the *Litsea*-*Cinnamomum* clade, a fruit receptacle that develops from the perianth tube (Fig. 3c, f).

Supplementary Note 10 *TGA10* expression in unisexual and bisexual flowers in Lauraceae

To investigate the differentially expressed genes involved in the development of bisexual and unisexual flowers, we first selected the differentially expressed genes (fold change > 2, p < 0.05, FDR) between the female and male flowers in species of *L. tsinlingensis*, *L. rubescens*, *L. cubeba*, *L. megaphylla*, and *S. tzumu*. KEGG pathway and GO term-enrichment analyses were performed for each species. Interestingly, the differentially expressed genes were observed to be enriched in the 'Plant Hormone Signal Transduction' (map04075) in each species. Unexpectedly, *TAG10* was included in the enriched 'Plant Hormone Signal Transduction' pathway in each of the above species. Subsequently, we analyzed the expression mode of *TAG10* according to the FPKM value in the transcriptomic data of unisexual and bisexual flower buds in Lauraceae.

After the identification of the differentially expressed genes (fold change > 2, p < 0.05, FDR) between the female and male flowers in *L. tsinlingensis*, *L. rubescens*, *L. cubeba*, *L. megaphylla*, and *S. tzumu*, we excluded the sequences with a mapping rate of less than 0.5 to obtain 34 common differentially expressed genes in five species, using the reciprocal-best-BLAST-hits (RBH) method[9]. To verify these genes, we blasted the 34 sequences to the genome of *L. cubeba* and obtained the two differentially expressed genes. We then investigated the mode of expression of the two genes: one was a hypothesized protein (Lcu01G_02292 in the 124099255-124107806 region in chr1 of the *L. cubeba* genome), which exhibited a differential expression mode in eight unisexual species and nine bisexual species of Lauraceae, with some exceptions in the flowers of *S. tzumu.*

Supplementary Note 11. MADS-box genes in *L. cubeba*

MADS-box transcription factors are among the most important regulators of plant floral development and are a major class of regulators that mediate floral transition. The *L. cubeba*

genome encodes 64 MADS-box genes, 46 of which are type II MADS-box genes (Supplementary Table 28). The number of MADS-box genes in *L. cubeba* is comparable to that in *C. kanehirae* (Lauraceae). However, there are more MADS-box genes in *L. cubeba* than there are in the early-diverging angiosperm *Amborella*, the early-diverging eudicot *Macleaya* (Papaveraceae), or the early-diverging orchid *Apostasia*, but fewer than in other angiosperm (for example, *Arabidopsis*, poplar, and rice). Interestingly, the *L. cubeba* genome contains a comparable number of MADS-box genes with that of the early-diverging angiosperm *Nymphaea* (Supplementary Table 27). However, the *L. cubeba* genome has twice the number of type II MADS-box genes and half that of type I MADS-box genes relative to the *Nymphaea* genome (Supplementary Table 27).

A previous work indicated that a minimum set of 21 MADS-box gene clades exist in the MRCA of extant angiosperm[10]. Our results showed that *Nymphaea* also contained the same 21 MADS-gene clades as *Amborella* (Supplementary Figure 15). Most of the type II MADS-box gene clades that existed in *L. cubeba* were also found in *C. kanehirae*, which are both members of Lauraceae, with exception of the *OsMADS32* clade (Supplementary Figure 15). This implies that *OsMADS32*-like genes were independently lost for the common ancestor of Lauraceae. Furthermore, *L. cubeba* contained a member of the *TM8* gene lineage (Supplementary Figure 15), which existed in both basal angiosperm *Amborella* and *Nymphaea*, suggesting that *L. cubeba* might retain the conserved function of the *TM8* gene lineage.

The *L. cubeba* genome reveals a comparable number of floral organ identity genes than *Amborella*. These floral organ identity genes, from eight major lineages (AP1/SQUA, AP3/DEF, PI/GLO, AG, STK, AGL2/SEP1, AGL9/SEP3, and AGL6), existed in the MRCA of extant angiosperms. Additional members of the *SOC1* clade may have been evolved through genome duplication and tandem duplication (Supplementary Figure 15). *SOC1* integrates multiple flowering signals derived from the photoperiod, temperature, hormone, and age-related signals[11]. Transcription of two *SOC1*-like genes (*LcuMADS21* and *LcuMADS22*) was dominantly detected in the vegetative tissues (Supplementary Figure 15), suggesting that they are major integrators of flowering signals. Differential expression among these *SOC1*-like

genes could be associated with the functional diversification of *SOC1* clade in *L. cubeba.*

Supplementary Note 12 Identification and functional analysis of DXS in Lauraceae

The genes involved in the MEP pathway exhibit a high level of specific expression during fruit development, which may have contributed to the biosynthesis of large amounts of monoterpene. The first and a key rate-limited enzyme of the MEP pathway was 1-deoxyxylulose 5-phosphate synthase (DXS)[12,13]. To elucidate the evolutionary relationships between the DXS genes, we performed phylogenomic analyses of protein-coding genes from 28 species, including *L. cubeba*, 21 species of Lauraceae, *C. praecox*, *Oryza sativa*, *Arabidopsis thaliana*, *Vitis vinifera*, *Solanum lycopersicum*, and *Populus trichocarpa*. A phylogenetic tree was constructed utilizing RAxML (v8.2.10)[14], adopting the GTR + JTT model to estimate a maximum likelihood tree. The *L. cubeba* genomes contained seven DXS genes, six of which were Clade B members. Expression pattern analyses revealed that *LcuDXS6* (Clade A) was constitutively expressed in tissues, and thought to participate in primary metabolism, including that of carotenoids and chlorophyll. The members *LcuDXS1*, *LcuDXS2*, *LcuDXS3*, *LcuDXS4*, *LcuDXS5*, and *LcuDXS7* of Clade B had high transcriptional levels in specialized tissue such as fruit, which was associated with the synthesis of essential oils. Moreover, from mixed transcriptome data for 22 species, we further found that the *DXS*s involved in Clade B expanded across *Litsea*, *Beilschmiedia*, and *Sassafras* (Supplementary Figure 16), which produced higher levels of essential oils in Lauraceae. Interestingly, the Clade A gene had a higher level of expression than the genes for Clade B and C, which may be attributed the constitutive expression pattern. Conserved motifs of the DXS domain were identified from a motif using the sequence analysis tool MEME Suite version 5.0.2[15], with the following parameters: for any number of motif repetitions, with the maximum number of motifs set at 40, and with an optimum width of 6-50 amino acids. The motifs 'MALAAFSFPGHLQRDVVLDPL' and 'LRNTSTSNSLFGGADLQYSFHHRILKGRKGPCVSASLSERG' were found to be common to most genera of Lauraceae, with exception of for *C. filiformis*, *D. hainanensis*, and *C. burmanni* (Supplementary Figure 16). Aanalysis of transient expression was performed on *L. cubeba* leaves to characterize the potential functions of *LcuDXS3*. Empty vector or constructs containing *LcuDXS3*, under the control of the Cauliflower mosaic virus 35S promoter, were

carried out using *Agrobacterium* strain LBA4404 cultures and infiltrated into the same leaves on the left and right sides, separated by the midrib. After infiltration, the plants were grown for 2 days. Leaves that were positioned close (<5 mm) to the infiltration point (without infiltration) were collected and immediately frozen in liquid nitrogen[16]. These samples were stored at -80°C for qRT-PCR and volatile analysis. Three biological replicates were used for transient overexpression analysis. The volatiles were analyzed via GC-MS[16], and 1 μg of ethyl decanoate added as an internal standard. These results indicated that the introduction of *LcuTPS22* or *LcuTPS42* into *L. cubeba* leaves accelerated monoterpene biosynthesis. The primers are shown in Supplementary Table 36.

Supplementary Note 13. Predictions of genes and non-coding RNA

The detailed procedure for the predictions of genes was as follow. First, spliced transcript evidence was generated by RNA-seq using Cufflinks[17] and Program to Assemble Spliced Alignments[18]. The obtained long ORFs were used for *ab initio* gene annotation and for an initial set of gene models. Next, *ab initio* gene prediction was conducted using five *ab initio* gene predictors based on a hidden Markov model, namely, Augustus[19], GlimmerHMM v.3.0.1[20], SNAP (version 2006-07-28)[21], Genscan[22], and Geneid[23]. Then, orthologous protein sequences were spliced against the unmasked *L. cubeba* genome using exonerate[24] to obtain final-splice protein results. Finally, Evidence Modeler[25], a combined software for weighted consensus, was employed to generate a single high-confidence gene model set. From the above annotation pipeline, 31,329 protein-coding genes were predicted (Supplementary Table 9).

Supplementary Note 14. Identification and functional analysis of TPSs in *L. cubeba*

Total RNA of different tissues of *L. cubeba* was extracted with an RN38 EASY spin plus Plant kit (Aidlab, Beijing). Quantitative real-time PCR analysis was performed using the SYBR® Premix Ex Taq TM Kit (TaKaRa, Tokyo, Japan), and all reactions were conducted using an ABI7300 Fast Real-Time quantitative instrument (Applied Biosystems, Foster City, CA, USA). The PCR program were 95°C for 30 s, followed by 40 cycles at 95°C for 5 s and 60°C for 31 s. The constitutive gene *UBC* was used as an internal control to normalize the gene expression of the chosen transcripts[26]. The relative expression levels of the selected genes were

compared to those of the controls measured by the $2^{-\Delta\Delta Ct}$ method. The primers are shown in Supplementary Table 36. Three biological and three technical replicates were implemented for each gene.

The biosynthesis of terpene is typically tissue-specific in plants[27,28], as exemplified by geraniol and nerol, which are specifically synthesized and stored in the fruits of *L. cubeba*. Digital expression analysis (Fig. 5) showed that *LcuTPS22* specifically accumulated in leaves and monoterpene genes (*LcuTPS18*, *LcuTPS19*, *LcuTPS20*, *LcuTPS25, LcuTPS26* and *LcuTPS42*) showed high transcriptional levels during the period of fruit development, which was consistent with the large amounts of geraniol- (~50%) and nerol- (~35%) derived compounds produced (Supplementary Table 29). The products of these TPS enzymes may be the main components of essential oils found in fruits.

Transient expression analysis of *LcuTPS22* and *LcuTPS42* in *L. cubeba* leaves was performed as mentioned above. One micro gram of ethyl decanoate was added to serve as an internal standard. The transient overexpression demonstrated that LcuTPS22 catalyzed the products of α-pinene, β-pinene, eucalyptol, and camphene. The geraniol levels of the leaves infiltrated with *LcuTPS42* showed an increase relative to the levels in leaves infiltrated with empty vector (a 4.99-fold change). These results indicated that the introduction of *LcuTPS22* and *LcuTPS42* into *L. cubeba* leaves accelerated monoterpene biosynthesis.

To determine the function of *LcuTPS* genes in monoterpene synthesis, transient expression analysis was performed on *N. benthamiana* leaves planted in a growth chamber at 26 °C with a 16 h light/8 h dark photoperiod. The empty vector and constructs containing *LcuTPS19*, *LcuTPS20*, *LcuTPS22*, *LcuTPS25,* or *LcuTPS42* were carried by *Agrobacterium* strain GV3101 cultures and then infiltrated into the leaves[29]. After infiltration, the *N. benthamiana* plants were grown for 2 d; then, the leaves near (<5 mm) the infiltration point was collected and immediately frozen in liquid nitrogen. These samples were then stored at -80 °C for volatile analysis. There were three biological replicates for transient overexpression analysis. The primers used are shown in Supplementary Table 36.

To determine the enzyme activity of *LcuTPS22*, *LcuTPS25* and *LcuTPS42*, the full-length open reading frames were cloned and inserted into the pET28a vector. After transformation into *Escherichia coli* BL21 (DE3) pLysS cells (Transgen, China), recombinant protein expression was induced with 0.2 mM isopropyl-β-d-galactopyranoside for 20h at 16 °C, and the expressed protein was purified with either Ni-NTA agarose (Clontech). SDS-PAGE was carried out using Tris- HCl buffer (pH 7.5) and the protein was visualized by Coomassie brilliant blue staining. For *in vitro* enzymatic assays, the recombinant protein was incubated with 25 mM HEPES, pH 7.2, 100 mM KCl, 10 mM $MgCl_2$, 10% (v/v) glycerol, 5 mM DTT and 30 μM geranyl diphosphate (GPP, Sigma) at pH 7.2 and 30°C for 1 h[30]. The volatiles were analyzed using GC-MS. To identify the target monoterpene, the retention time was compared with that of authentic standard purchased from Sigma-Aldrich, which was further validated using the NIST Mass Spectral Library. There were three biological replicates for analysis of enzyme activity. The primers used are shown in Supplementary Table 36.

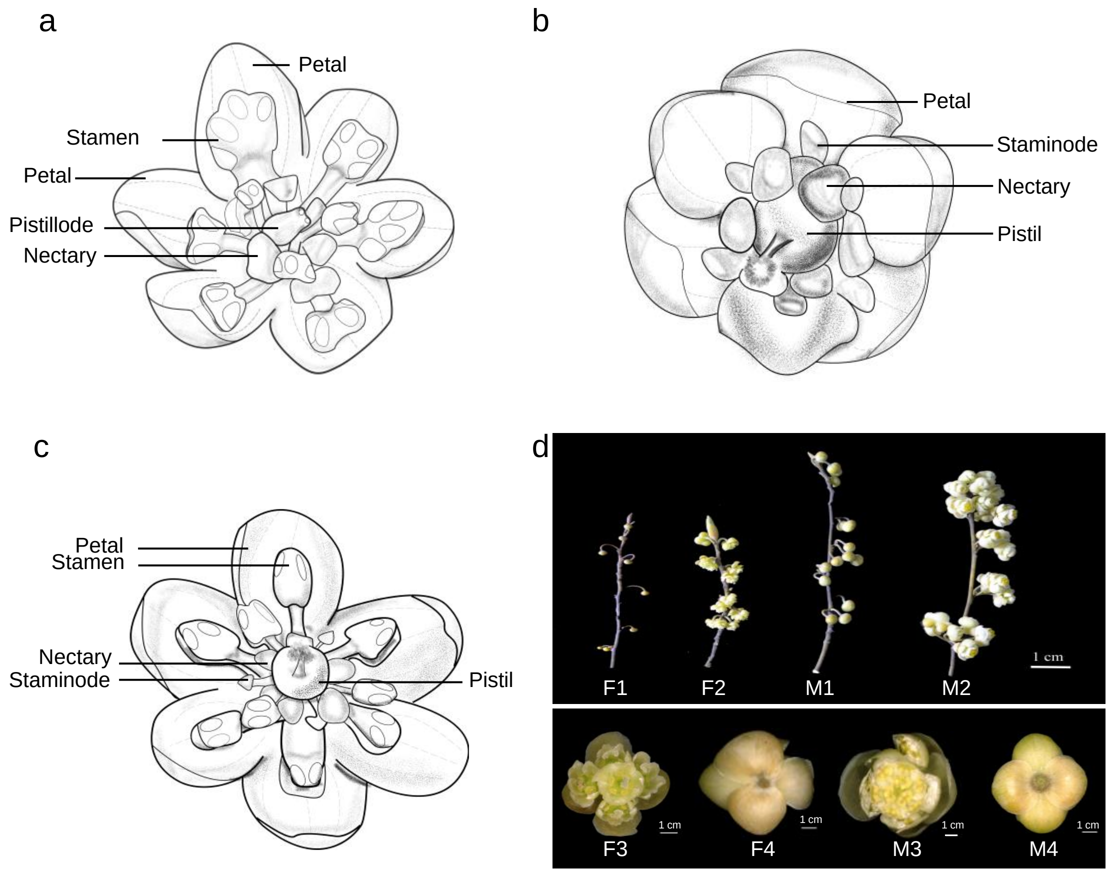

Supplementary Figure 1. The morphology of flowers in Lauraceae.

a. Illustration of a male flower in Lauraceae. b. Illustration of a female flower in Lauraceae. c. Illustration of a bisexual flower in Lauracea. d. Image of *L. cubeba* flowers. *L. cubeba* has a dioecious and unisexual flower, and the umbels of the male and female flower often have four to six flowers. The male flower has six perianth segments, which are broadly ovate. Nine stamens have filaments that are hairy below their middles, with three whorls each and two shortly stipitate glands at the base, and the pistillode is glabrous. The female flower has an oval ovary and six staminodes, pubescent in the lower part. F1-F4, female flower; M1-M4, male flower.

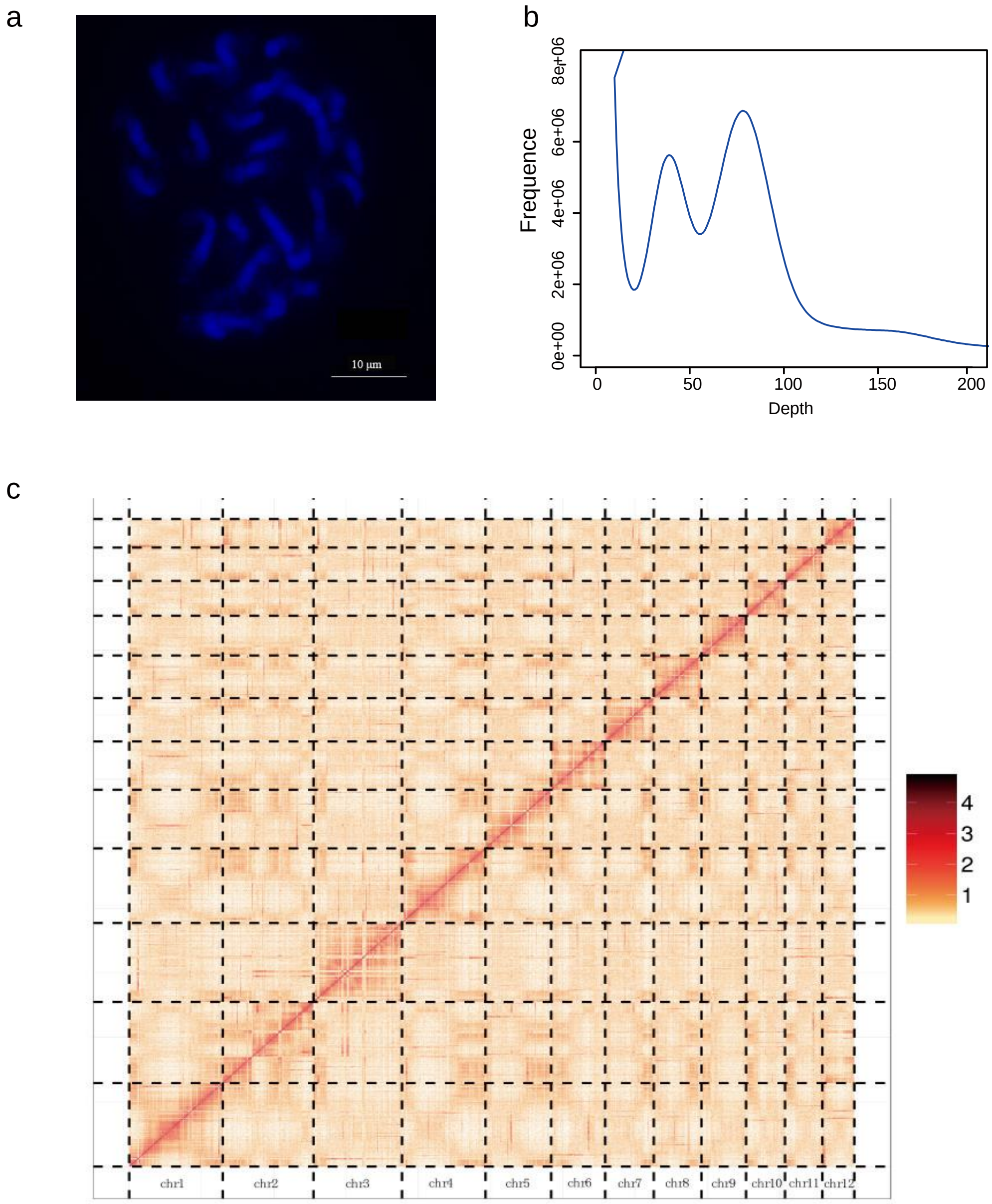

Supplementary Figure 2. Genome information for *L. cubeba* in chromosomes.

a. The karyotype of *L. cubeba*. The basic root-tip metaphase cells show the number of chromosomes number (2n = 24). b. The k-mer distribution of sequencing reads. According to the distribution, we estimated the genome size of *L. cubeba* as 1370.14 Mbp, with a 1% heterozygosity rate and a 70.59% repeat sequence, based an analysis of k-mer numbers/depths. c. Interchromosomal Hi-C contact map of *L. cubeba*. The intensity of each pixel represents the number of Hi-C links of 500 kb resolution in the chromosomes. Darker red pixels denote higher contact probabilities. Most interactions were observed within the chromosomes.

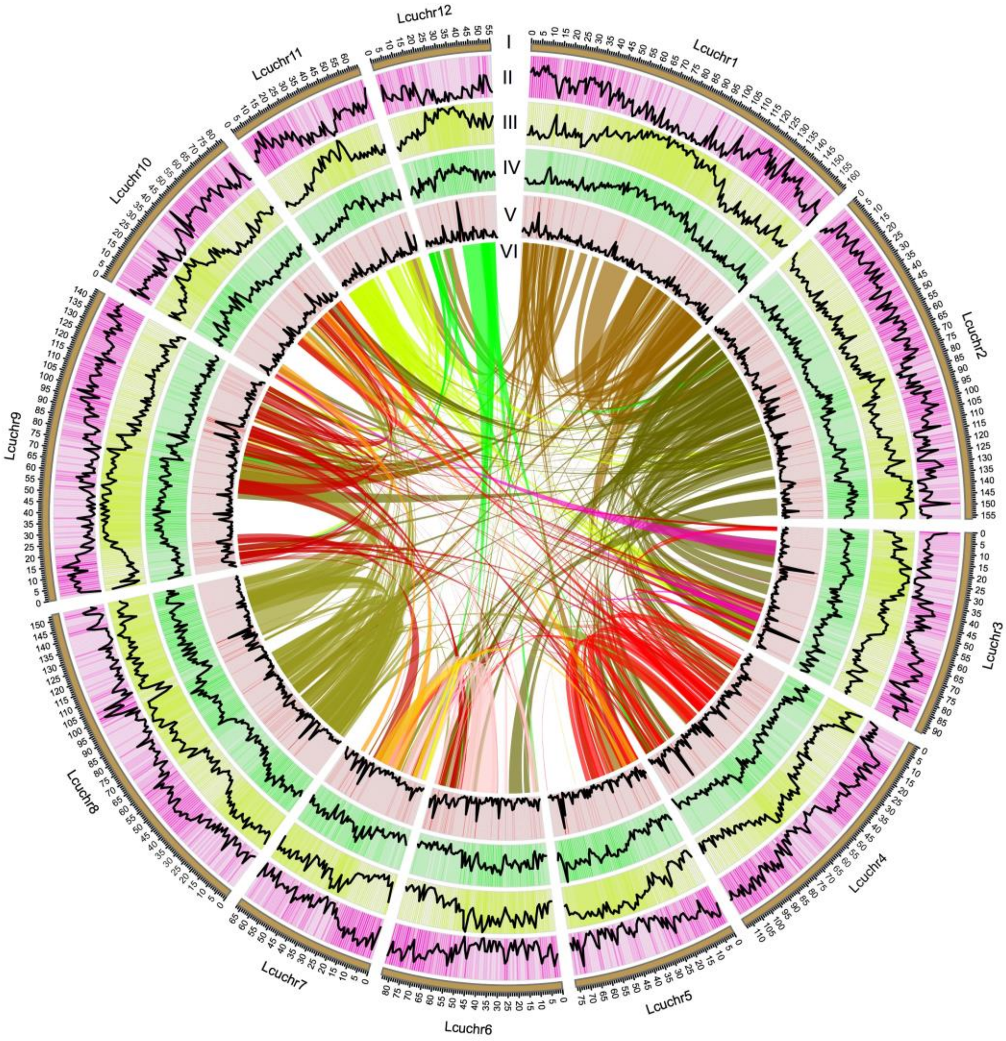

Supplementary Figure 3. Genomic structure of *L. cubeba*.

Chromosome-level assembly of the *L. cubeba* genome, with (i) circular representation of the *L. cubeb* 12 pseudochromosomes (on an Mb scale), (ii) gene density represented as number of genes per Mb, (iii) TE distribution, (iv) GC content in 1 Mb windows, (v) gene expression level, with the transcription level estimated from read counts per million mapped reads in 1 Mb windows, and (vi) synteny of the *L. cubeba* genome. This Figure was generated using Circos (http://circos.ca/).

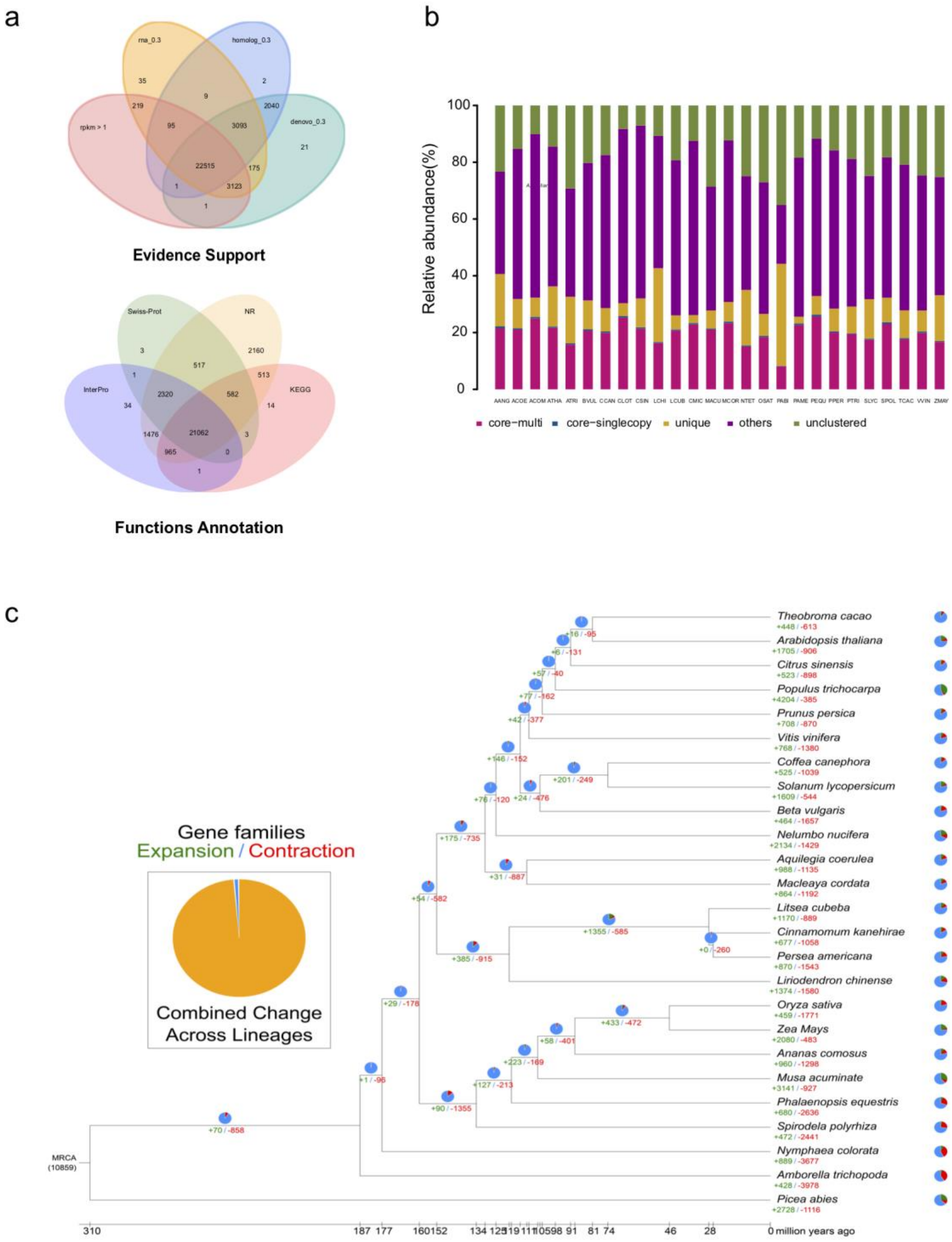

Supplementary Figure 4. Gene structural and functional annotation of the *L. cubeba* genome.

a. Gene functions of the *L. cubeba* genome. Evidence for gene structural annotation in *L. cubeba*. Three methods were employed, including *de novo* prediction, homology searching, and RNA-seq mapping. c. Evidence for gene annotation in *L. cubeba*. b. Orthologous genes in

L. cubeba and other species. The distribution of orthologous genes in *L. cubeba* and other 26 species. Core-multi denotes genes with orthologs in all other species and might have paralogs in the species of one family. Core-single copy refers to genes with orthologs in all other species and no other paralogs in this species within one family. Unique means genes for which only one family contains genes of this species. Other orthologs describe genes not included in the other mentioned categories. Finally, unclustered genes are not clustered into any family. AANG, *Anthoceros angustus*; ACOE, *Aquilegia coerulea*; ACOM, *Ananas comosus*; AOFF, *Asparagus officinalis*; ATHA, *Arabidopsis thaliana*; ATRI, *Amborella trichopoda*; BVUL, *Beta vulgaris*; CCAN, *Coffea canephora*; CKAN, *Cinnamomum kanehirae*; CLOT, *Nelumbo nucifera*; CSIN, *Citrus sinensis*; LCHI, *Liriodendron chinense*; LCUB, *Litsea cubeba*; MACU, *Musa acuminate*; MCOR, *Macleaya cordata*; NTET, *Nymphaea colorata*; PABI, *Picea abies*; PAME, *Persea americana*; PDAC, *Phoenix dactylifera*; PEQU, *Phalaenopsis equestris*; PPER, *Prunus persica*; PTRI, *P. trichocarpa*; SLYC, *Solanum lycopersicum*; SPOL, *Spirodela polyrhiza*; TCAC, *Theobroma cacao*; VVIN, *V. vinifera*. c. Phylogenetic tree showing the evolution of gene-family size. This tree shows the expansion and contraction of gene families for 26 plant species. Numbers at branches indicate the expansion and contraction of gene families. MRCA = most recent common ancestor. Numbers in parentheses represent the number of gene families in the MRCA, as estimated by CAFÉ[31] (version 4.2).

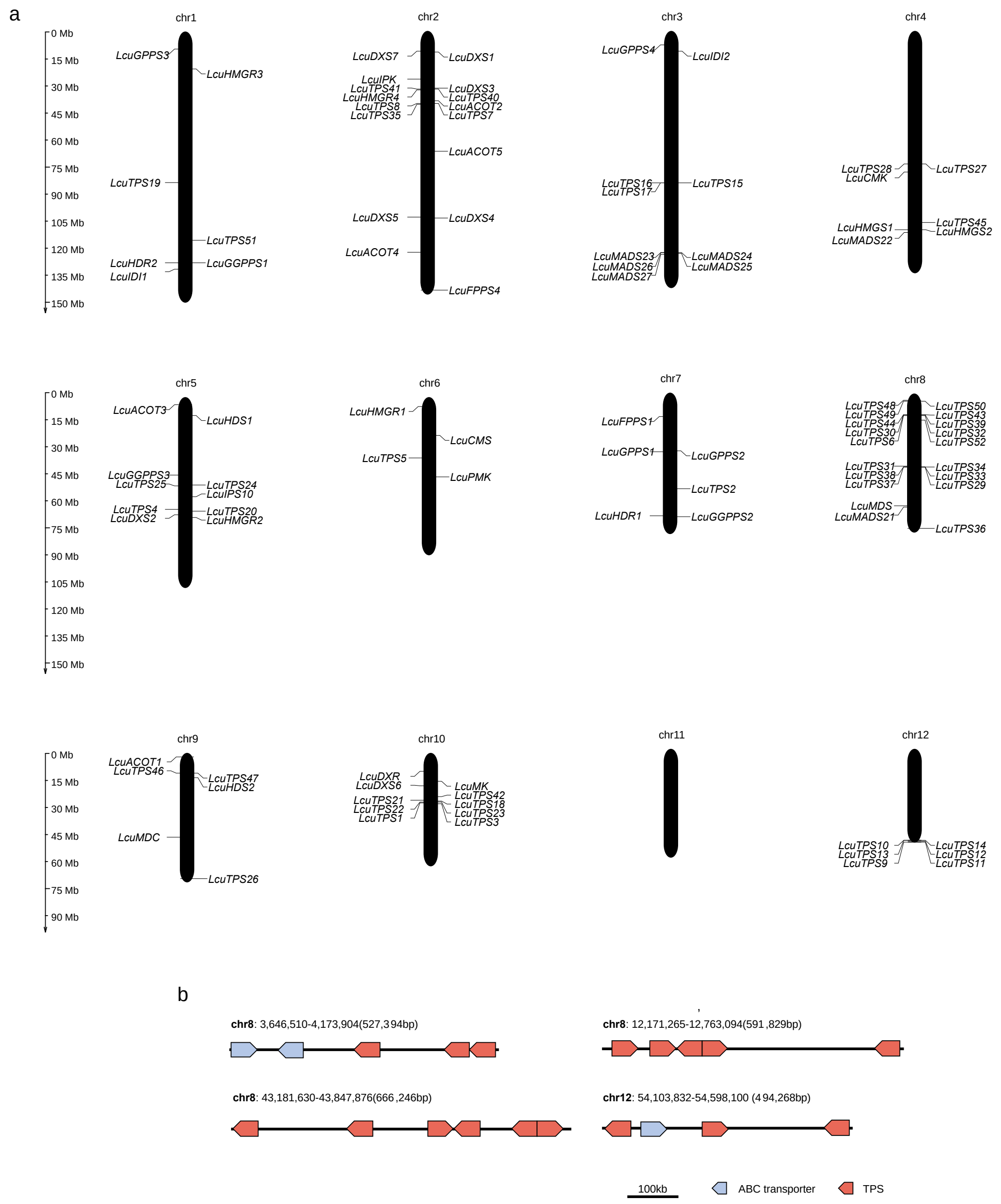

Supplementary Figure 5.

Chromosome location of terpene biosynthesis genes and SOC1 clade genes.

a Tandem and chromosome segmental duplication may contribute to the expansion of *LcuTPSs*, *LcuDXSs* (1-deoxyxylulose 5-phosphate synthase), and SOC1-like genes (*LcuMADS21* to *LcuMADS27*). *LcuTPSs*, *LcuDXSs*, and *SOC1-like* genes were not uniformly distributed across the chromosomes, and some clusters of gene members from individual subfamilies were observed as tandem duplicates (Supplementary Tables 28 and 32). Terpene biosynthesis gene clusters, including *DXS* (the five expanded members with high expression in fruit), *ACOT*, *IPK*, *HMGR*, and *TPS* family members, were found in the 31821 kb (11,756,903

-43,577,938 and 31,821 kb) region of Chromosome 2 (chr2). The terpenoid biosynthase coding gene cluster is referred to as the *TPS* cluster and is found in region 39969 kb (3,878,818-43,847,876 and 44,985 kb) of chromosome 8 (chr8). b. Tandem and chromosome segmental duplication of *LcuTPS*, *ABC transporter members* in chr8 and 12 (other annotated genes are not shown).

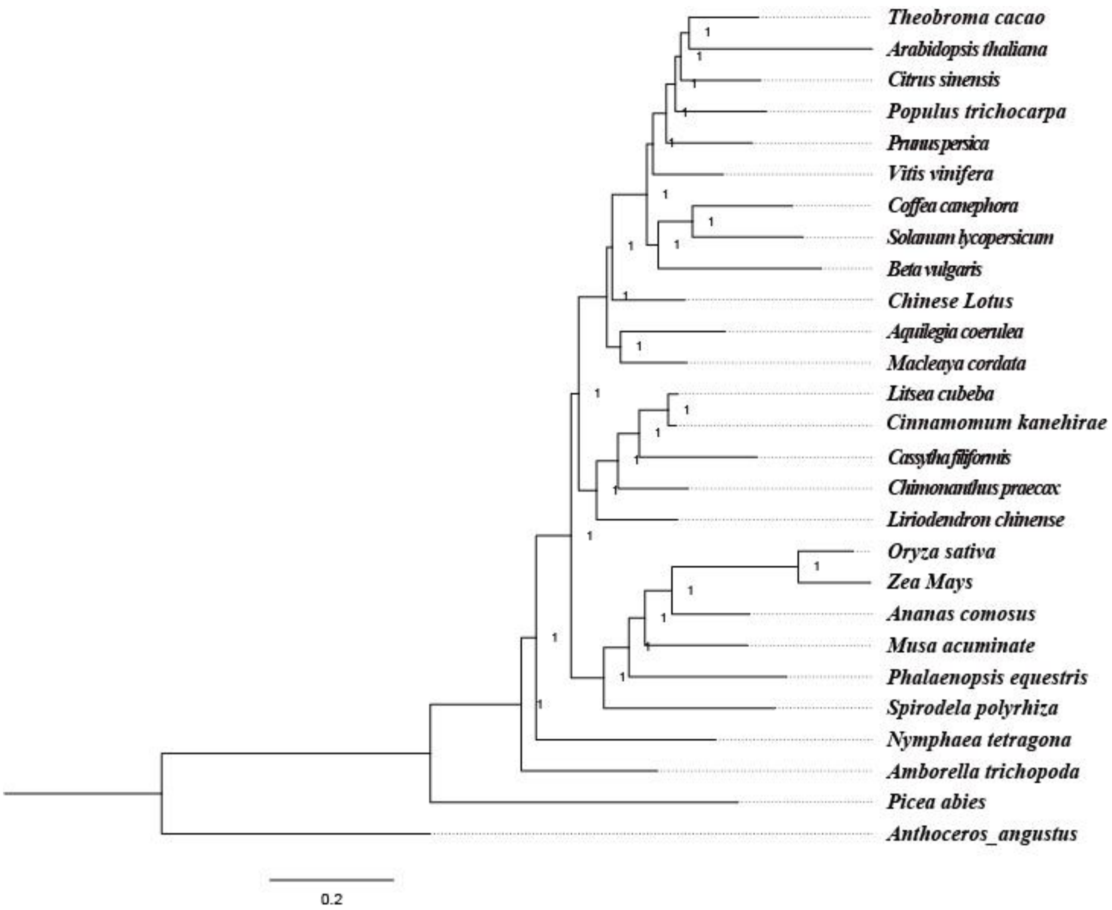

Supplementary Figure 6. Phylogenomic tree of angiosperm with Gramineae.

We developed a phylogenetic tree based on a concatenated sequence alignment of 401 single-copy gene families of *L. cubeba* and of 26 other plant species (including Gramineae species) using MrBayes[32]. Source data are provided as a Source Data file.

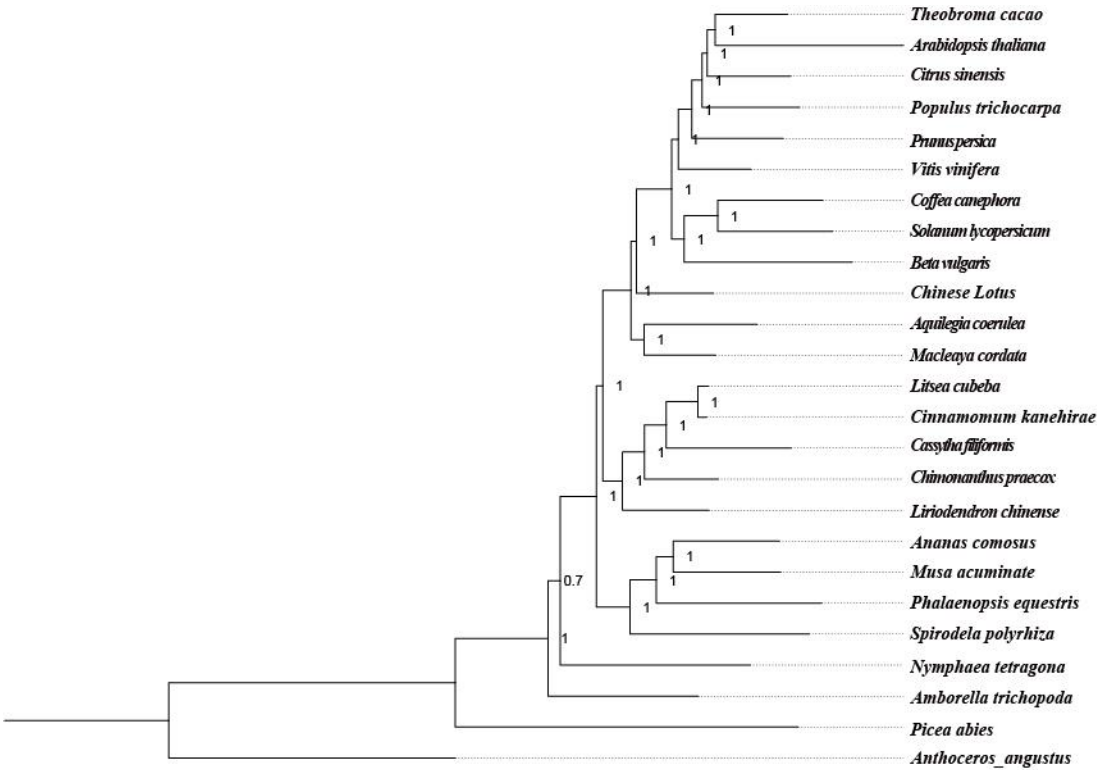

Supplementary Figure 7. Phylogenomic tree of angiosperm without Gramineae.
Phylogenomic tree of angiosperm. We developed a phylogenetic tree based on a concatenated sequence alignment of 308 single-copy gene families from *L. cubeba* and 24 other plant species (not including Gramineae species) using MrBayes[32]. Source data are provided as a Source Data file.

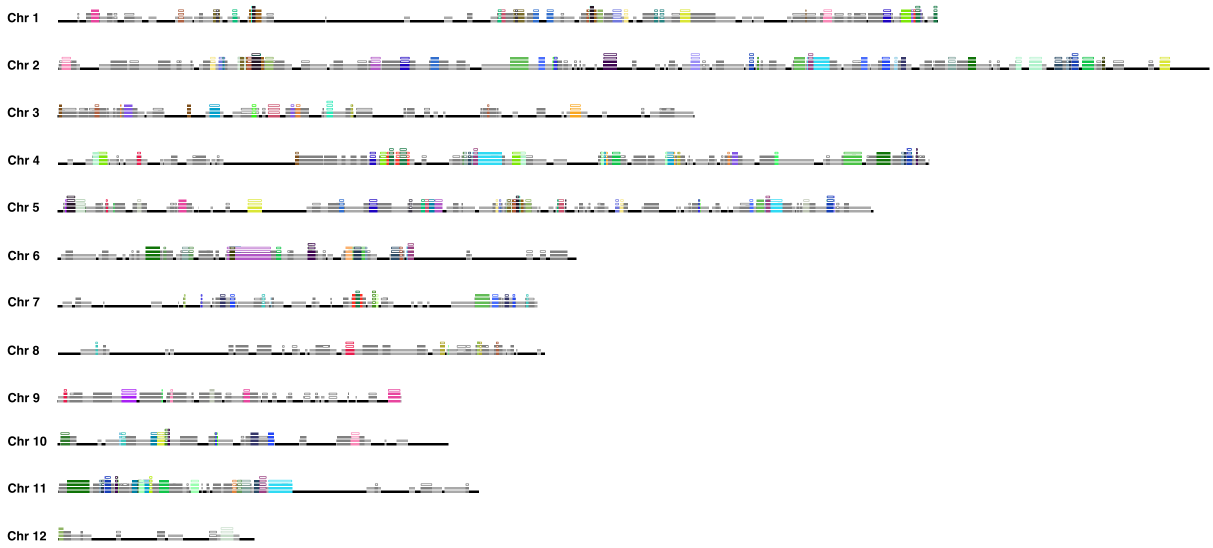

Supplementary Figure 8. Within genome collinearity of *L. cubeba*.

Diagram of collinear/syntenic segments for all chromosomes of the *L. cubeba* genome i. The height of the stack reflects the number of segments that are colinear with the segment on the particular chromosome. Black denotes regions with no collinear segments; light gray denotes regions with two copies of collinear segments; dark gray denotes regions with three copies of collinear segments; and regions with chromatic colors have more than three collinear/syntenic segments in the genome. An empty rectangle indicates that the identified collinear segment only shares a limited number of paralogs with the reference genome and can only be identified through recursive construction of genomic profiles based on relatively recent collinear segments[33,34].

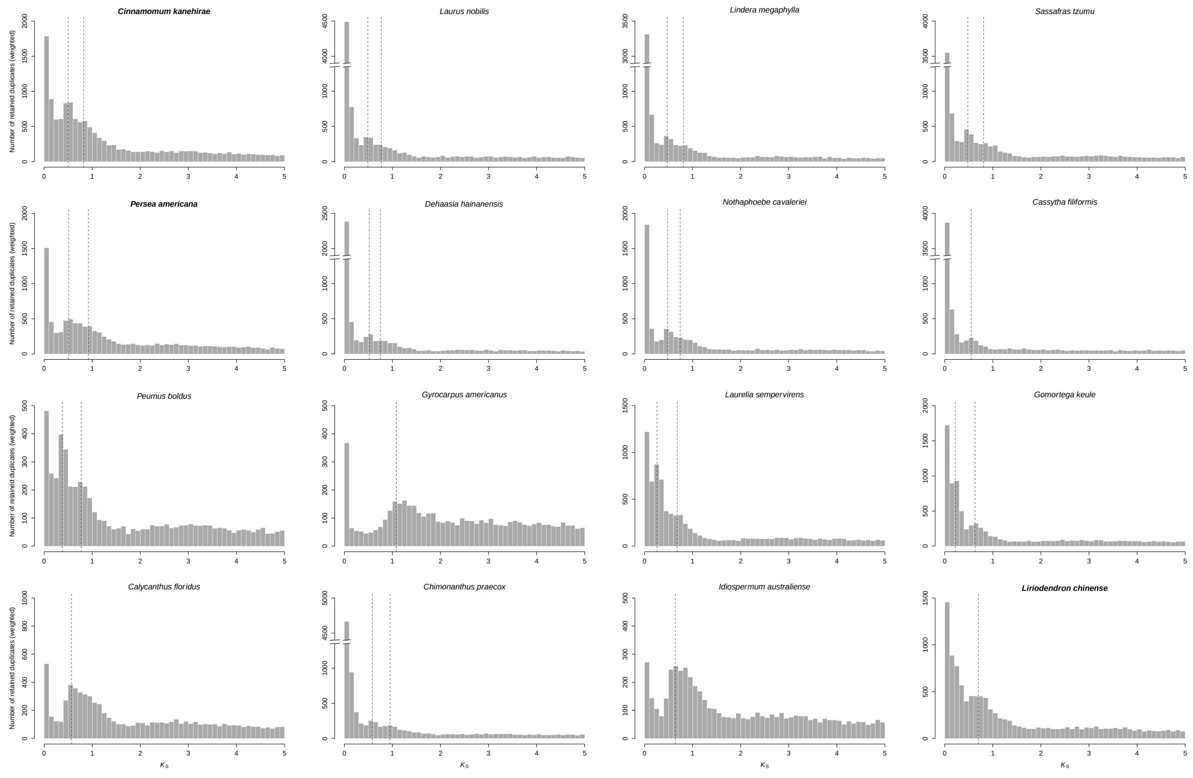

Supplementary Figure 9. *K*$_S$ Distributions of the whole paranome for 16 Lauralean genomes and transcriptomes.

K$_S$ distributions of paralogs are shown in grey and identified peaks are denoted by dotted lines. The two light grey rectangles in the background of each plot highlight the *K*$_S$ peak ranges found in the genome of *L. cubeba* from 0.3-0.645 and 0.645-1.1, respectively (Fig. 2a). The names of species with sequenced genomes are in bold.

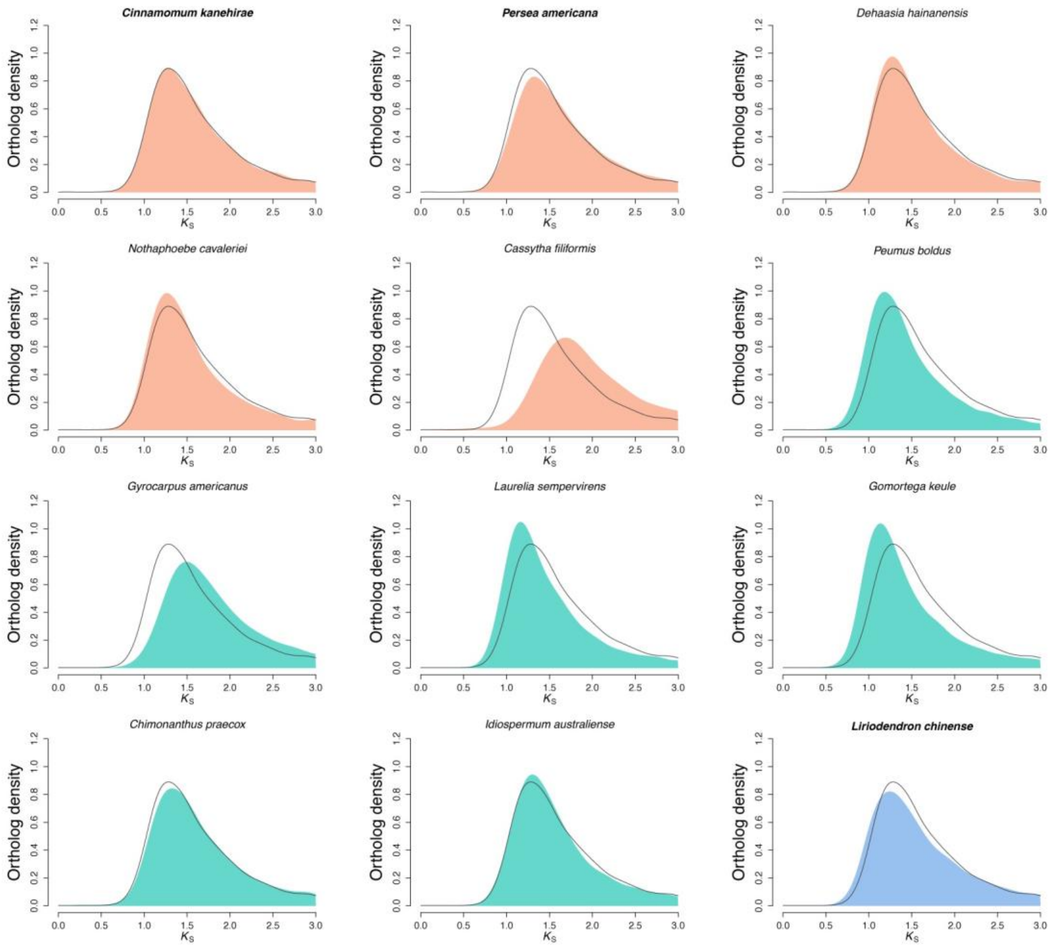

Supplementary Figure 10. K_S Distributions between *V. vinifera* and species from Laurales and Magnoliales.

In each plot, the kernel density estimation (KDE) of an orthologous K_S distribution between *V. vinifera* and a species (color filled) is compared with the KDE of the orthologous K_S distribution between *V. vinifera* and *L. cubeba* (dark grey line). The color of red denotes species in Lauraceae, the color of green denotes species from Laurales but not in Lauraceae, and the color of blue denotes a species from Magnoliales. The names of species with sequenced genomes are in bold.

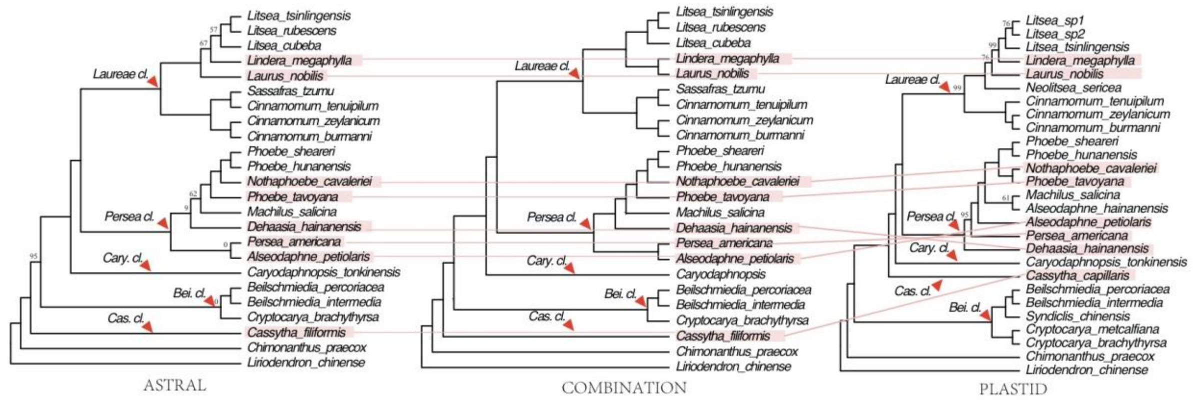

Supplementary Figure 11. The phylogenetic trees of Lauraceae based on three methods. The three methods included MSC tree (left) and BI tree (middle) by single-copy genes, and ML tree by plastid genome (right). The red arrows refer to main taxonomic clades. The red lines and bars display the topological differences among methods. Number close to node is the support value, and only the values below 100 or 1.0 (in BI tree) are marked.

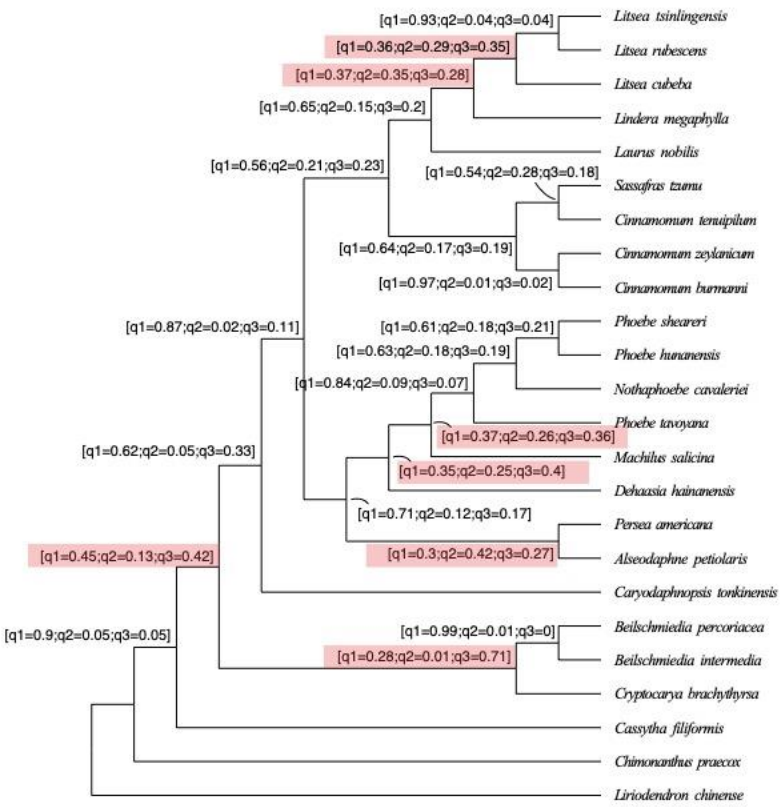

Supplementary Figure 12. The Lauraceae phylogenetic trees annotated with Q value by ASTRAL.

The Quartet score of main topologies (q1) under 50 was considered as significant phylogenetic discordance signals (red bar).

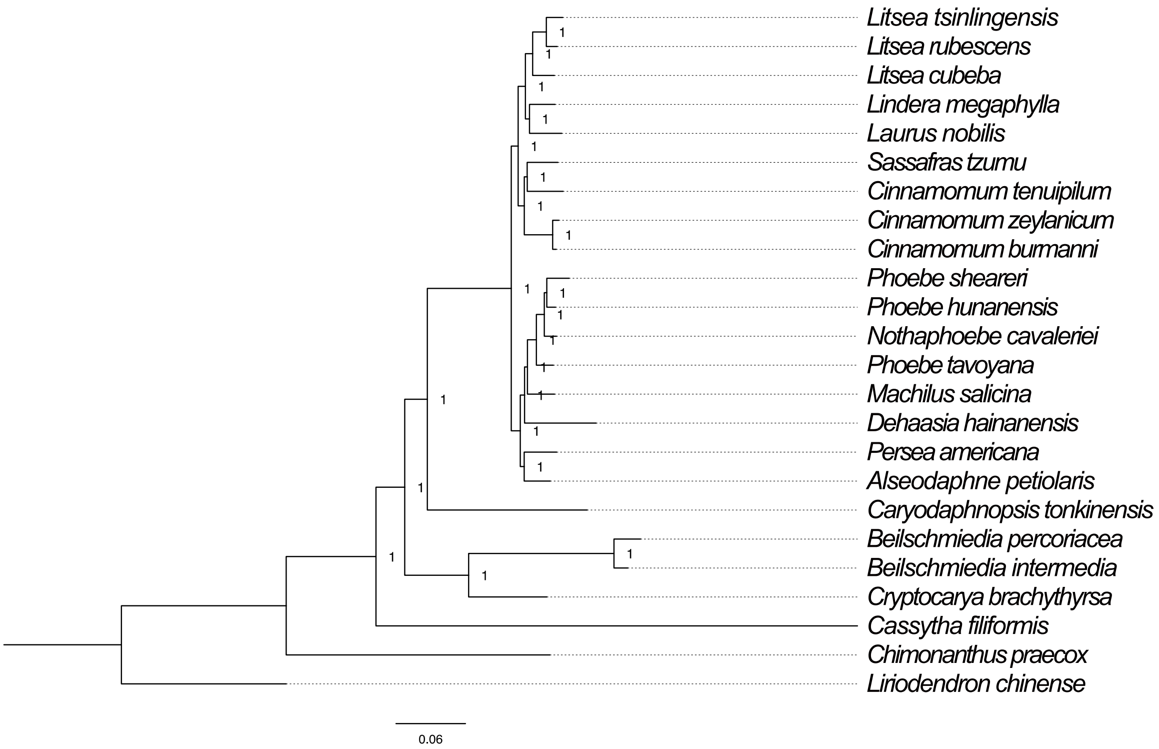

Supplementary Figure 13. The Phylogenomic tree of Lauraceae.

Phylogenomic tree using MrBayes[32] was constructed based on a concatenated sequence alignment of 275 single-copy gene families from 22 species in *Lauraceae*. Source data are provided as a Source Data file.

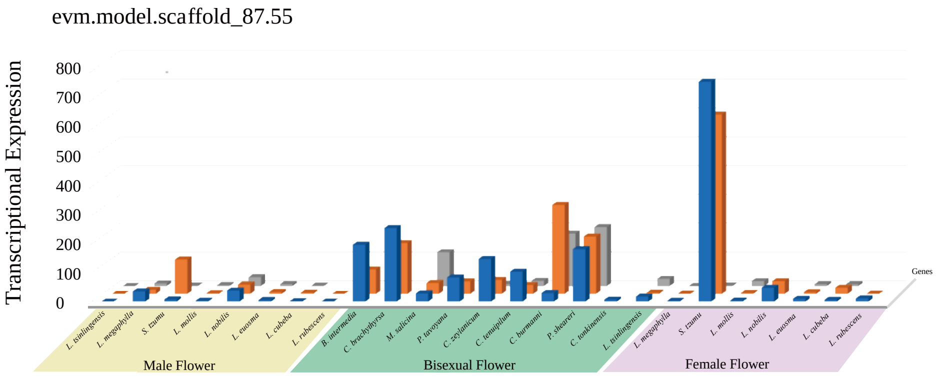

Supplementary Figure 14. The expression modes of Lcu01G_02292 in flower buds of Lauraceae.

A hypothesized protein (Lcu01G_02292 in region of 124099255-124107806 in chr1 of *L. cubeba* genome) exhibiting a differentiated expression mode in eight unisexual species and nine bisexual species of Lauraceae with some exceptions in flowers of *S. tzumu*. Source data are provided as a Source Data file.

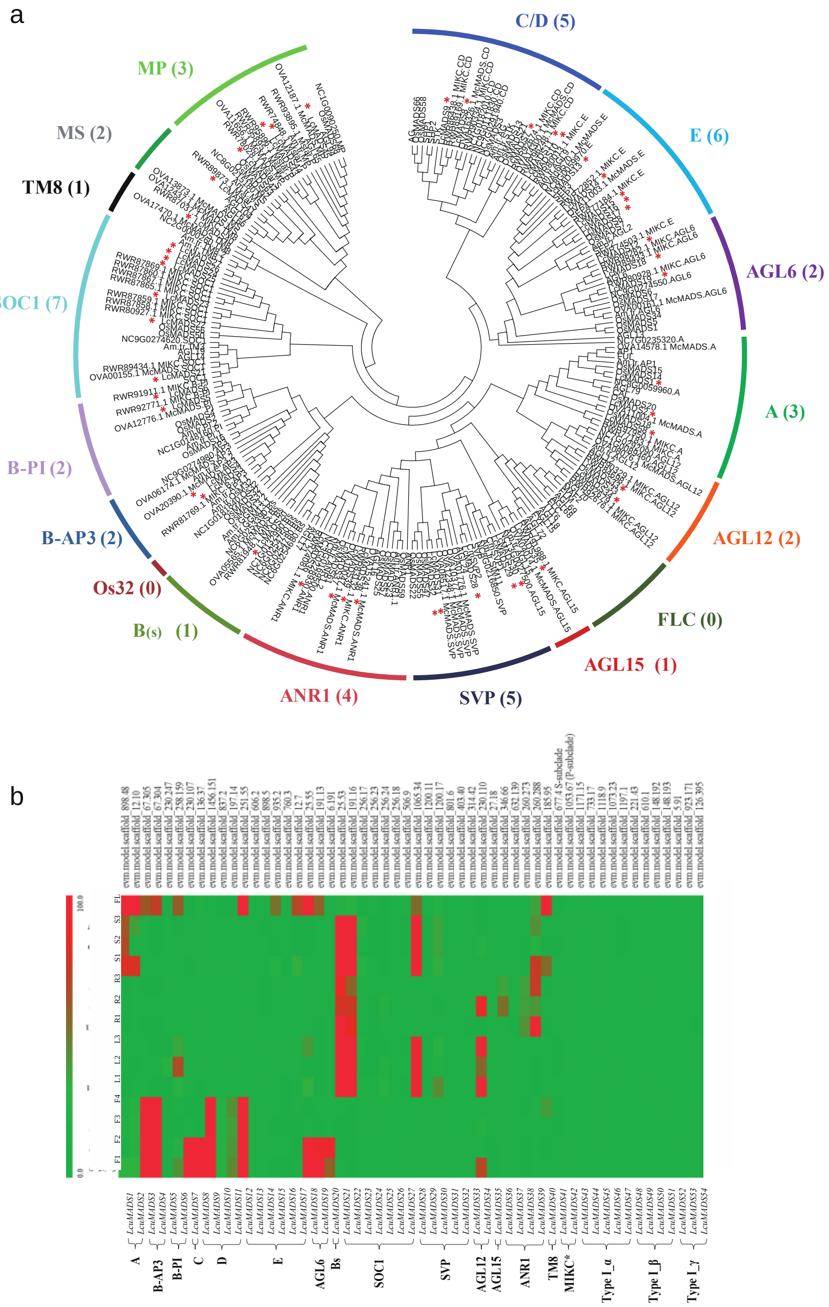

Supplementary Figure 15. MADS-box genes involved in *L. cubeba* morphological evolution.

a Compared to the basal angiosperm *Amborella* and *Nymphaea* (both have 21 MADS-gene clades), most type II MADS-box gene clades exist in *L. cubeba*, with exception of the OsMADS32 clade. Furthermore, the eudicot specific TM8 gene lineage has orthologs in *L. cubeba* and in *Amborella*, *Nymphaea*, and *Macleaya*. b Expression patterns of MADS-box genes in *L. cubeba*. Most of these genes are highly expressed in reproductive tissues, indicating

that these genes play important roles in the floral development of *L. cubeba*. The transcripts of two *SOC1*-like genes (*LcuMADS21* and *LcuMADS22*) are dominantly detected in vegetative tissues, suggesting these two genes are major integrators of flowering signals. R, root; S, stem; L, leaves; FL, flower; F1, fruit 40 days after full bloom; F2, fruit 70 days after full bloom; F3, fruit 100 days after full bloom; F4, fruit 100 days after full bloom. Source data underlying Supplementary Figure 15a are provided as a Source Data file.

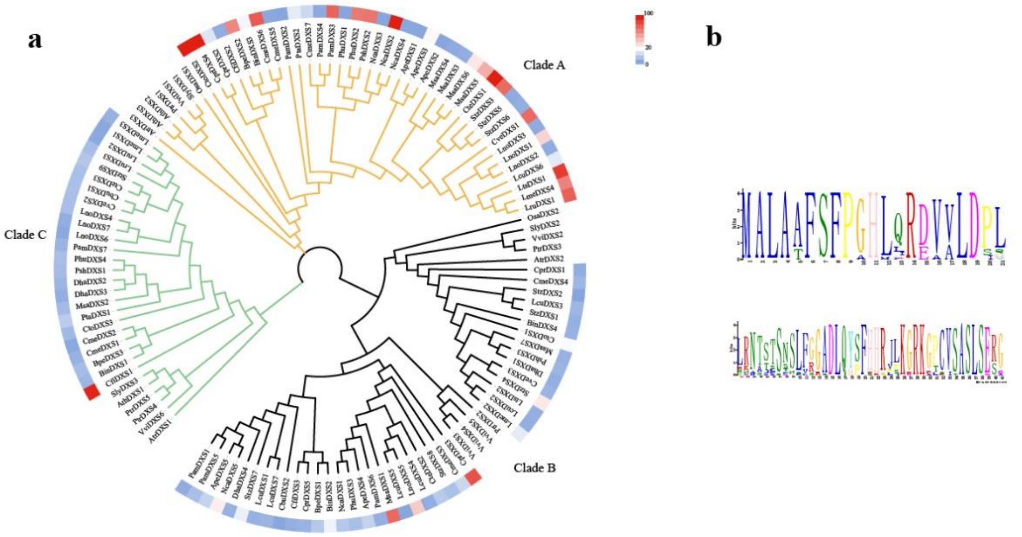

Supplementary Figure 16. Phylogenetic and expression analysis of *DXS* genes.

DXS is a rate-limiting enzyme of the MEP pathway in 22 species of Lauraceae. The levels of expression of *DXS* (FPKM value) are represented by the color bar. b. Two conserved motifs identified in most genera of Lauraceae except for *C. filiformis*, *D. hainanensis*, and *C. burmanni*. Source data underlying Supplementary Figure 16a are provided as a Source Data file.

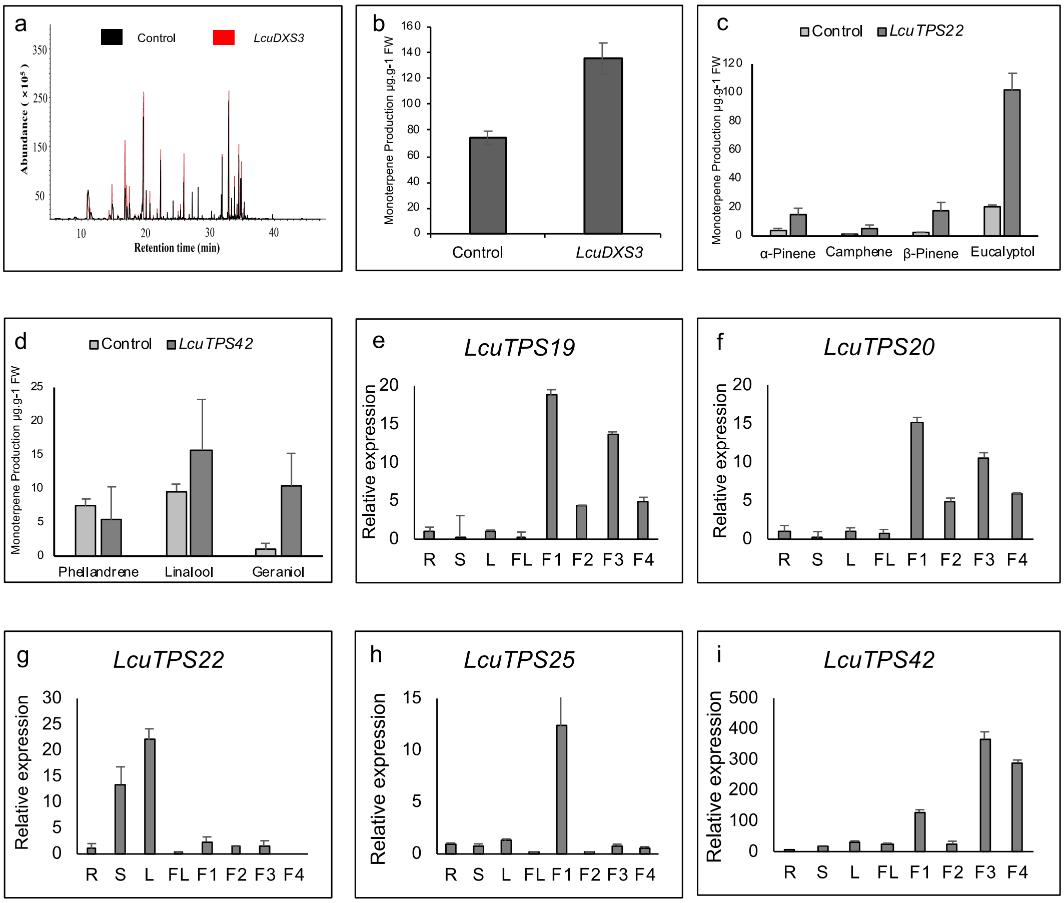

Supplementary Figure 17. Functional verification on LcuDXS3 and LcuTPSs.

a, b. Transient overexpression of *LcuDXS3* resulted in about 2-fold increase of monoterpene in *L. cubeba* leaves. After infiltration, the plants were grown for 2 d and the monoterpene was detected using GC-MS.. c, d. Transient overexpression of *LcuTPS22* and *LcuTPS42* in *L. cubeba* leaves. After infiltration, the plants were grown for 2 d and the monoterpene was detected using GC-MS. e-i. qRT-PCR verification of the expression mode of *LcuTPS19*, *LcuTPS20*, *LcuTPS22*, *LcuTPS25* and *LcuTPS42*. *LcuTPS22* is highly expressed in leaves, and the others exhibit higher expression level in fruits. *LcuTPS25*, 42 are highly expressed in early young fruits when the essential oil begins to be produced in *L. cubeba*. Data represent the mean ± SDs of three biological replicates. Source data underlying Supplementary Figure 17b-i are provided as a Source Data file.

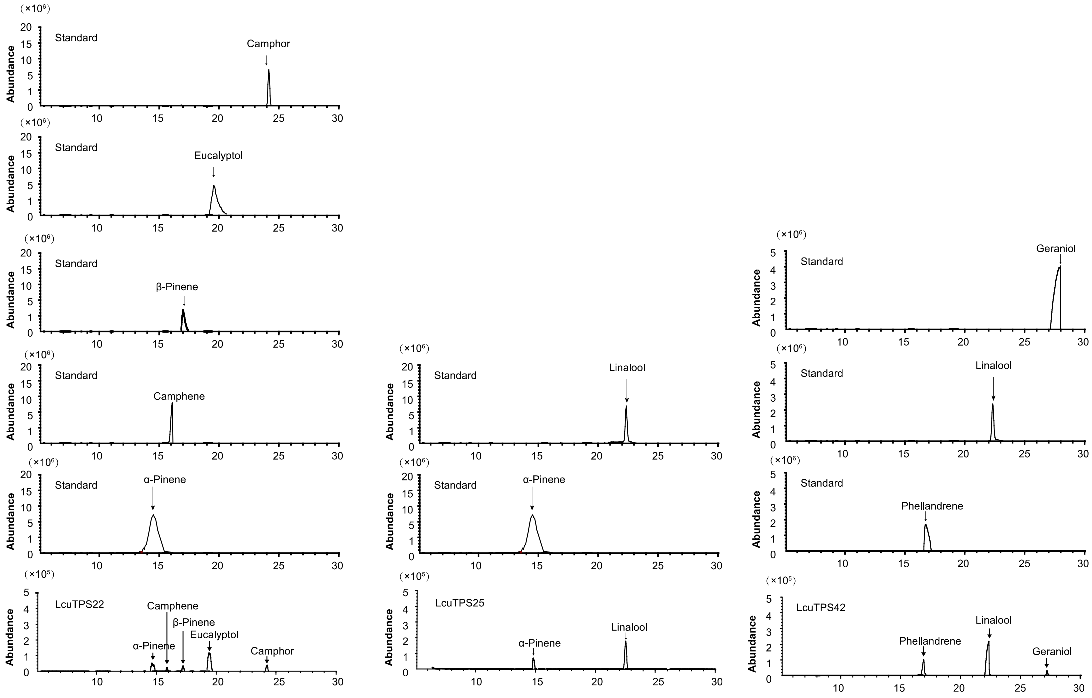

Supplementary Figure 18. The authentic standard used for analyzation of LcuTPS in GC-MS.

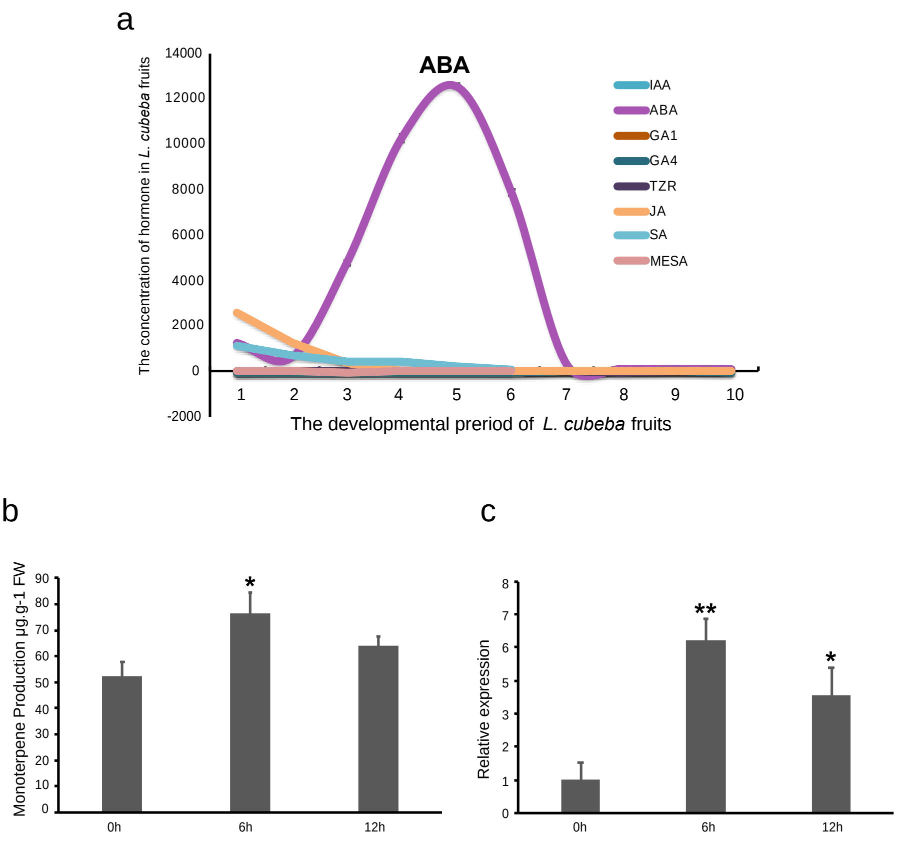

Supplementary Figure 19. Endogenous abscisic acid content and treatment of ABA in *L. cubeba.*

a. The content of endogenous abscisic acid (ABA) during the development of *L. cubeba* fruits. b. The treatment of ABA on *L. cubeba* leaves. c. The induced expression of *LcuTPS22* in *L. cubeba* leaves after treatment with ABA. Data represent the mean ± SDs of three biological replicates. Source data underlying Supplementary Figure 19b and 19c are provided as a Source Data file.

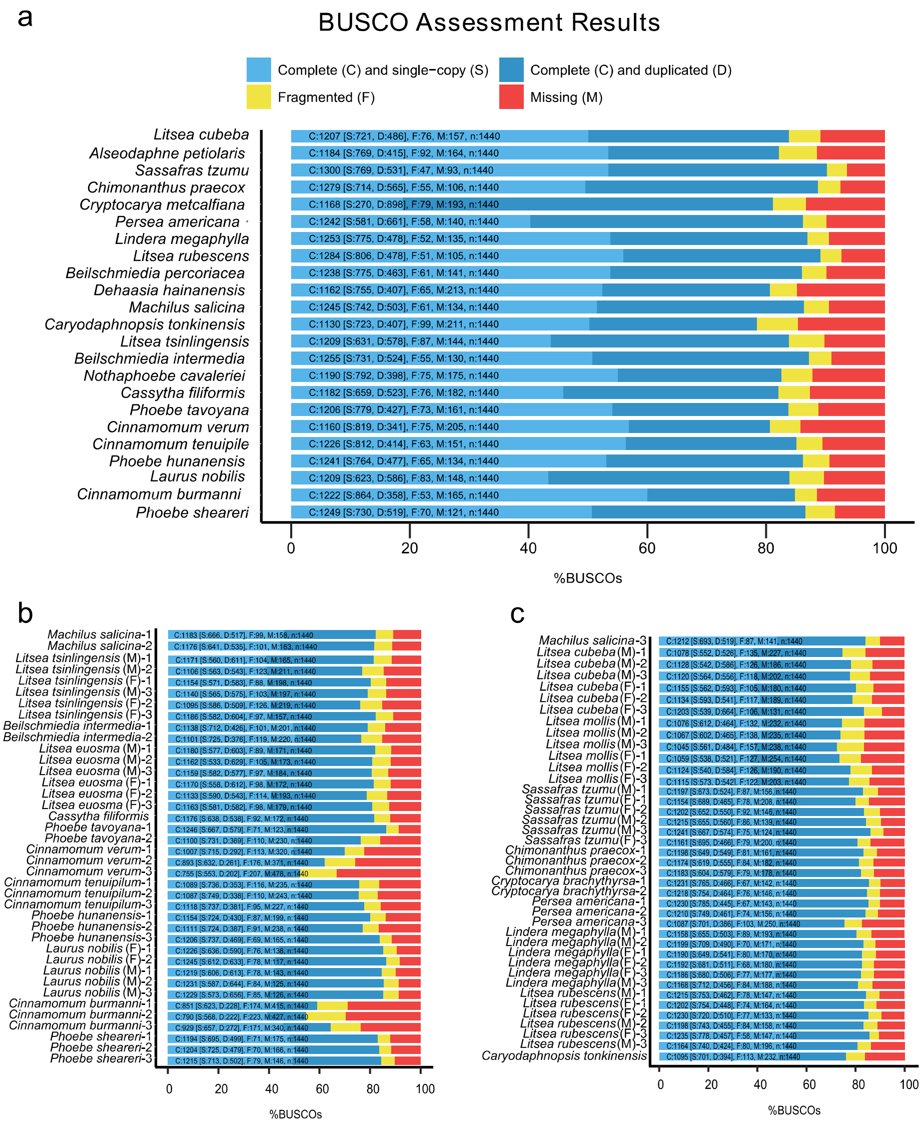

Supplementary Figure 20. BUSCO assessments of transcriptome assembly.

a. A BUSCO assessment of the mixed-tissue transcriptome assemblies for 23 species, representing 16 genera (Supplementary Table 23). The completeness was found to be generally more than 80%. Transcriptome completeness was given as ‘complete plus fragmented’. b. A BUSCO assessment of the flower bud transcriptomes for 21 species, representing 13 genera (Supplementary Table 26). Most transcriptome assemblies, except *Cinnamomum burmanni-1, -2, -3* and *Cinnamomum verum-3*, were found to have a completeness close to or exceeding 80%.

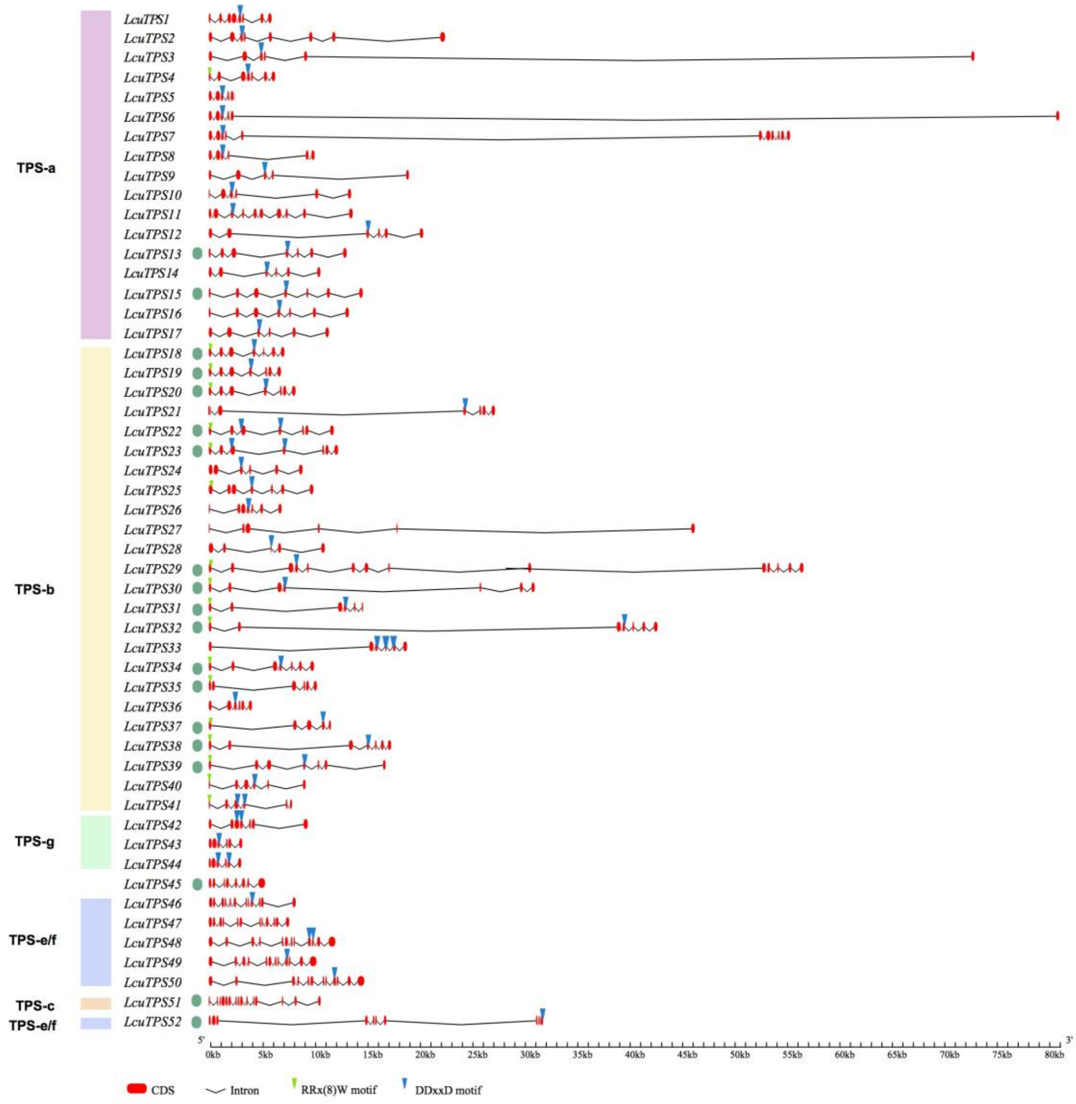

Supplementary Figure 21. Gene structure and classification of the *LcuTPS* family. Exon-intron structures were predicted. Red boxes and black lines represent at scale protein coding exons and introns, respectively. The conserved motifs RR(x)8W and DDxxD are represented by green and blue triangles, respectively. Green circles indicate the prediction of an N- terminal plastidial targeting peptide. Classification into subfamilies was based on phylogenetic analyses.

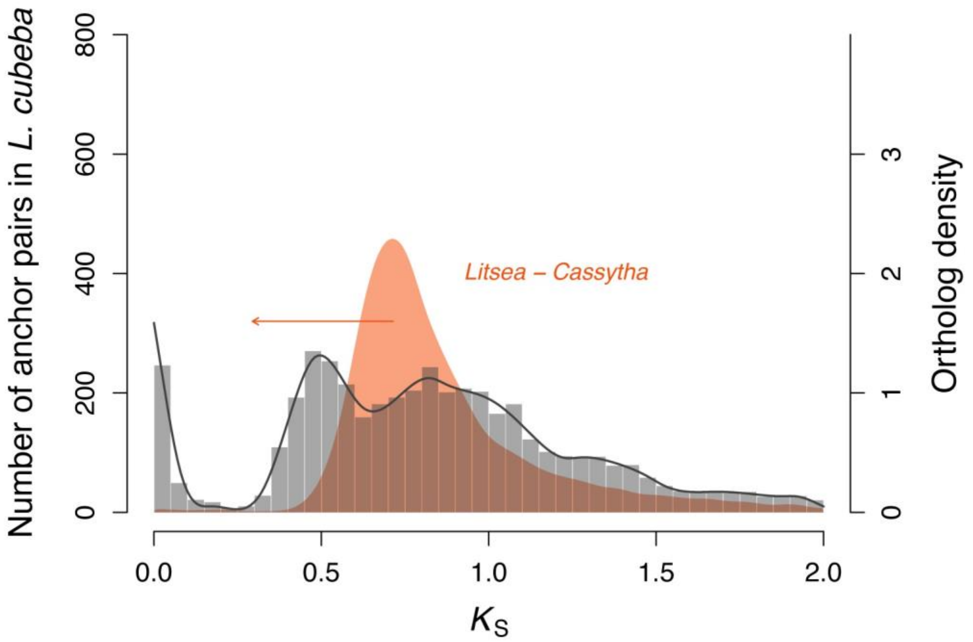

Supplementary Figure 22. *K_S* Distributions for anchor pairs of *L. cubeba* and for orthologs of *L. cubeba* and *C. filiformis*.

Distributions of synonymous substitutions per synonymous site (K_S) for paralogs found in collinear regions (anchor pairs) of *L. cubeba* (dark grey histogram and line, left-hand y-axis; peaks represent WGD events) and for one-to-one orthologs between *L. cubeba* and *C. filiformis* (colored filled curves of kernel-density estimates, right-hand y- axis; a peak represents a species divergence event). The arrows in red indicates an overestimation of the divergence event between *L. cubeba* and *C. filiformis*. The head of the arrow points to the K_S values after correction of differences in substitution rate between the two species based on *L. cubeba* (see Methods).

Supplementary Table 1. ***L. cubeba*** **sequencing data statistics.**

Pair-end libraries	Insert size	Total data (G)	Read length (bp)	Sequence coverage (X)
Illumina reads	350bp	359.77	150	262.58
Pacbio reads	-	213.25	-	155.64
10X Genomics	-	143.28	150	104.57
Total	-	716.30	-	522.79

Supplementary Table 2. Assembly statistics of the *L. cubeba* genome.

Sample ID	Length		Number	
	Contig* (bp)	Scaffold (bp)	Contig*	Scaffold
Total	1,313,552,199	1,325,587,569	3,669	1,514
Max	4,317,025	10,220,425	-	-
Number ≥ 2000	-	-	3,669	1,514
N50	607,340	1,759,806	627	220
N60	477,094	1,418,648	871	304
N70	365,825	1,077,290	1,186	411
N80	265,320	761,030	1,607	559
N90	168,889	447,104	2,217	785

* Contig after scaffolding.

Supplementary Table 3. Hi-C assembly data for *L. cubeba*.

	Input assembly	LACHESIS assembly
Total length	1,325.59 Mb	1,325.69 Mb
Scaffold L50/N50	220 scaffolds; 1.76 Mb	5 scaffolds; 113.31Mb
Scaffold L90/N90	785 scaffolds; 0.45 Mb	11 scaffolds; 64.38 Mb
Longest scaffolds	10.22 Mb	10.22 Mb
Number of scaffolds	1,154	509
Contig N50	607.34 kb	607.34 kb

Supplementary Table 4. CEGMA evaluation of *L. cubeba* genome assembly.

Species	Complete		Complete + partial	
	*Prots	% Completeness	*Prots	% Completeness
L. cubeba	223	89.82	238	95.97

* Prots, number of assembled core genes.

Using tblastn, genewise, and geneid software, Core Eukaryotic Genes Mapping Approach (http://korflab.ucdavis.edu/dataseda/cegma/) was used to determine the genome assembly. A core gene database was constructed from 248 core eukaryotic genes of six model species, including *Arabidopsis thaliana*, *Caenorhabditis elegans*, *Drosophila melanogaster*, *Homo sapiens*, *Saccharomyces cerevisiae*, and *Schizosaccharomyces pombe*. We assembled 238 genes (95.97%), which indicated the fine quality of the genome assembly.

Supplementary Table 5. BUSCO assessment of *L. cubeba* genome assembly and annotation.

	Genome count	Ratio (%)	Protein count	Ratio (%)
Complete BUSCOs	1272	88.4	1284	89.2
Complete single-copy BUSCOs	1182	82.1	1130	78.5
Complete duplicated BUSCOs	90	6.3	154	10.7
Fragmented BUSCOs	42	2.9	72	5
Missing BUSCOs	126	8.7	84	5.8
Total BUSCO group searched	1440		1440	

Supplementary Table 6. Assessment of *L. cubeba* genome assembly using mRNA sequences of *L. cubeba*.

Dataset	Number	Total length (bp)	Sequences covered by assembly (%)	With >90% sequence in one scaffold**		With >50% sequence in one scaffold	
				Number	Percent (%)	Number	Percent (%)
>0 bp	93,790	63,676,995	98.687	84,901	90.522**	91,833	97.913
>200 bp	93,790	63,676,995	98.687	84,901	90.522**	91,833	97.913
>500 bp	35,121	46,477,568	99.245	30,397	86.549	34,453	98.098
>1 k	18,676	34,883,400	99.470	15,790	84.547	18,337	98.185
>2 k	5,909	16,753,702	99.577	4,682	79.235	5,773	97.698

Supplementary Table 7. Statistics of repeat sequences in the *L. cubeba* genome.

	Denovo + Repbase*		TE Proteins**		Combined TEs**	
	Length (bp)	% in Genome	Length (bp)	% in Genome	Length (bp)	% in Genome
DNA element	46,384,309	3.50	27,616,001	2.08	63,026,842	4.75
LINE***	19,595,945	1.48	23,332,745	1.76	36,404,413	2.74
SINE***	158,824	0.01	0	0.00	158,824	0.01
LTR***	613,295,749	46.27	203,565,779	15.36	631,460,170	47.64
Satellite	2,037,158	0.15	0	0.00	2,037,158	0.15
Simple Repeat	1,636,429	0.12	0	0.00	1,636,429	0.12
Unknown***	18,554,430	1.40	0	0.00	18,554,430	1.40
Total	694,105,991	52.36	253,470,797	19.12	735,345,025	55.47

Genomic scaffolds were masked by RepeatMasker (http://www.repeatmasker.org) with default options after using RepeatModeler/RepeatScout/Piler/LTR_finder software with RepBase database prediction.

* Denovo + Repbase denotes transposable elements identified by RepeatMasker (http://www.repeatmasker.org), with default options after RepeatModeler/RepeatScout/Piler/LTR_finder software for use with RepBase database prediction.

** TE proteins were transposable elements, identified in the genome through the annotation of Repeat ProteinMask software, using the RepBase database; combined TEs involve a combination of the above two methods.

*** LINE, long interspersed nuclear element; SINE, short interspersed element; LTR, long terminal repeat; Unknown repeat sequences could not be clustered by Repeat Masker.

Supplementary Table 8. Statistics for TEs in several species.

Species	Genome size	TEs %	LTR %	Gypsy length (Mb)	Gypsy %	Copia length (Mb)	Copia %
C. kanehirae	730.7	47.84	25.53%	335.66	10.40	196.70	6.10
L. cubeba	1325.69	55.47	47.64%	389.58	29.39	210.98	15.92
L. chinense	1742.4	61.64	56.25%	704.67	40.45	227.86	13.08

LTR/Copia and LTR/Gypsy accounted the largest components of the *L. cubeba*, *C. kanehirae*, *L. chinense* genomes.

Supplementary Table 9. Prediction of gene structures of the *L. cubeba* genome.

Gene set		Number	Average transcript length (bp)	Average CDS length (bp)	Average exon length (bp)	Average intron length (bp)	Average exons per gene
De novo	Augustus	21,870	5,917.21	936.45	252.11	1,834.94	3.71
	Glimer HMM	104,685	11,044.73	515.69	174.86	5,401.88	2.95
	SNAP	86,003	21,481.05	758.48	162.96	5,670.48	4.65
	Genscan	73,430	11,792.32	937.63	171.17	2,424.13	5.48
	Geneid	128,683	4,148.28	551.63	162.48	1,501.64	3.4
Homolog	*Amborella trichopoda*	88,915	2,596.5	738.83	337.54	1,562.51	2.19
	Nelumbo_nucifera	55,784	4,413.66	1,095.51	361.49	1,634.16	3.03
	Arabidopsis_thaliana	59,234	3,220.42	811.06	316.04	1,538.24	2.57
	Oryza_sativa	59,109	3,147.92	1,104.48	443.02	1,368.62	2.49
	Macleaya_cordata	92,331	2,835.95	784.39	345.27	1,613.06	2.27
	Vitis_vinifera	55,587	3,631.84	851.61	303.38	1,538.49	2.81
	Citrus_sinensis	53,472	3,946.28	1,237.08	417.02	1,377.72	2.97
RNA-seq	Cufflinks*	67,191	14,127.58	1,769.88	316.56	2,691.76	5.59
	PASA	48,165	8,635.05	1,012.57	219.96	2,115.31	4.6
EVM		42,505	7,550.41	1,010.33	232.6	1,956.02	4.34
PASA-update		42,303	7,618.36	1,017.63	234.16	1,972.83	4.35
General gene set		31,329	9034.05	1145.48	232.33	2007.04	4.93

* Including the UTR region.

Supplementary Table 10. Statistics on the annotation of the *L. cubeba* genome.

Database		Number annotated	Percent annotated (%)
NR		29,595	94.5
Swiss-Prot		24,488	78.2
KEGG		23,140	73.9
	All	25,859	82.5
InterPro	Pfam	23,905	76.3
	GO	17,370	55.4
Annotated		29,651	94.6
Total		31,329	

Supplementary Table 11. Gene-clustering statistics for 26 species.

Species	Genes	Unclustered genes	Clustered genes	Families	Unique families	Unique families genes	Common families	Common families genes	Single copy	Average genes per family
Anthoceros angustus	16511	3847	12664	7738	586	3042	3074	3664	132	1.637
Aquilegia coerulea	24794	3799	20995	13141	652	2549	3074	5341	132	1.598
Ananas comosus	21445	2175	19270	12364	371	1453	3074	5474	132	1.559
Arabidopsis thaliana	27404	3976	23428	13022	892	3875	3074	6064	132	1.799
Amborella trichopoda	26846	7850	18996	12781	1012	4396	3074	4359	132	1.486
Beta vulgaris	22904	4631	18273	12658	608	2326	3074	4848	132	1.444
Coffea canephora	25574	4465	21109	13651	617	2102	3074	5217	132	1.546
Nelumbo nucifera	24124	1708	22416	13779	466	2454	3074	5273	132	1.627
Citrus sinensis	30806	5935	24871	15200	571	1518	3074	6506	132	1.636
Liriodendron chinense	16511	3847	12664	7738	586	3042	3074	3664	132	1.637
Litsea cubeba	24794	3799	20995	13141	652	2549	3074	5341	132	1.598
Cinnamomum kanehirae	26531	3325	23206	14080	259	748	3074	6193	132	1.648
Musa acuminate	36515	10424	26091	13202	835	2340	3074	7807	132	1.976
Macleaya cordata	21911	2698	19213	12669	336	1508	3074	5236	132	1.517
Nymphaea tetragona	31589	7877	23712	12467	1057	6182	3074	4882	132	1.902
Oryza sativa	27694	7493	20201	13499	758	2152	3074	5211	132	1.496
Picea abies	71158	24964	46194	16728	5517	25553	3074	5903	132	2.761
Phalaenopsis equestris	17870	2093	15777	10991	352	1163	3074	4707	132	1.435
Persea americana	24616	4519	20097	13784	259	596	3074	5705	132	1.458
Prunus persica	26873	4246	22627	14206	574	2136	3074	5503	132	1.593
Populus trichocarpa	41331	7779	33552	14941	1058	3896	3074	8154	132	2.246
Solanum lycopersicum	34682	8615	26067	14232	1030	4814	3074	6204	132	1.832
Spirodela polyrhiza	19591	3585	16006	11538	410	1685	3074	4637	132	1.387
Theobroma_cacao	29445	6133	23312	14491	601	2854	3074	5336	132	1.609
Vitis_vinifera	26346	6513	19833	13176	643	1953	3074	5367	132	1.505
Zea mays	40557	10220	30337	15578	1854	6548	3074	6903	132	1.947

Supplementary Table 12. KEGG enrichment analysis of significantly expanded gene families in Lauraceae.

Map ID	Map title	*P* value	Adjusted *P* value	x	y	n	N	GO level
map00920	Sulfur metabolism	4.17E-37	6.25E-36	22	61	135	30806	Over
map01110	Biosynthesis of secondary metabolites	2.20E-33	3.29E-32	63	2457	135	30806	Over
map00902	Monoterpenoid biosynthesis	7.32E-33	1.10E-31	21	74	135	30806	Over
map00270	Cysteine and methionine metabolism	1.82E-24	2.72E-23	22	203	135	30806	Over
map01230	Biosynthesis of amino acids	2.27E-16	3.40E-15	22	480	135	30806	Over
map01100	Metabolic pathways	8.71E-13	1.31E-11	54	4563	135	30806	Over
map01120	Microbial metabolism in diverse environments	1.90E-12	2.85E-11	22	750	135	30806	Over
map00073	Cutin, suberine, and wax biosynthesis	1.58E-09	2.36E-08	9	111	135	30806	Over
map04120	Ubiquitin-mediated proteolysis	5.08E-07	7.61E-06	10	282	135	30806	Over
map00940	Phenylpropanoid biosynthesis	5.11E-07	7.67E-06	11	355	135	30806	Over
map04626	Plant-pathogen interaction	4.43E-06	6.65E-05	14	733	135	30806	Over

Supplementary Table 13. GO enrichment analysis of significantly expanded gene families in Lauraceae.

GO ID	GO term	GO class	*P* value	Adjusted *P* value	x1	x2	n	N	GO level
GO:0010333	Terpene synthase activity	MF	1.81E-34	2.34E-32	22	77	135	30806	Over
GO:0000287	Magnesium ion binding	MF	8.26E-28	1.07E-25	22	145	135	30806	Over
GO:0003824	Catalytic activity	MF	1.55E-19	2.00E-17	86	8184	135	30806	Over
GO:0006979	Response to oxidative stress	BP	5.34E-12	6.88E-10	11	119	135	30806	Over
GO:0005488	Binding	MF	6.27E-12	8.08E-10	82	9868	135	30806	Over
GO:0004601	Peroxidase activity	MF	1.00E-11	1.29E-09	11	126	135	30806	Over
GO:0043167	Ion binding	MF	1.06E-11	1.36E-09	56	5177	135	30806	Over
GO:0006633	Fatty acid biosynthetic process	BP	2.63E-10	3.40E-08	9	91	135	30806	Over
GO:0016747	Transferase activity, transferring acyl groups other than amino-acyl groups	MF	4.06E-10	5.24E-08	13	283	135	30806	Over
GO:0004713	Protein tyrosine kinase activity	MF	9.37E-10	1.21E-07	22	1038	135	30806	Over
GO:0006468	Protein phosphorylation	BP	1.10E-09	1.42E-07	24	1253	135	30806	Over
GO:0008152	Metabolic process	BP	1.17E-09	1.51E-07	71	8588	135	30806	Over
GO:0004672	Protein kinase activity	MF	1.25E-09	1.61E-07	24	1261	135	30806	Over
GO:0016740	Transferase activity	MF	1.52E-09	1.96E-07	37	2890	135	30806	Over
GO:0005515	Protein binding	MF	1.09E-08	1.41E-06	40	3553	135	30806	Over
GO:0004185	Serine-type carboxypeptidase activity	MF	1.49E-08	1.93E-06	7	65	135	30806	Over
GO:0046872	Metal ion binding	MF	2.32E-08	2.99E-06	31	2350	135	30806	Over
GO:0019538	Protein metabolic process	BP	1.99E-06	0.000257	31	2877	135	30806	Over
GO:0055114	Oxidation reduction	BP	2.35E-06	0.000303	20	1372	135	30806	Over
GO:0020037	Heme binding	MF	9.44E-06	0.001218	11	481	135	30806	Over
GO:0005524	ATP binding	MF	2.17E-05	0.002798	24	2160	135	30806	Over
GO:0016491	Oxidoreductase activity	MF	2.29E-05	0.002948	20	1602	135	30806	Over
GO:0005506	Iron ion binding	MF	0.000154	0.019809	9	443	135	30806	Over

Supplementary Table 14. KEGG pathway enrichment of unique gene families in Lauraceae.

Map ID	Map title	*P* value	Adjusted *P* value	x	y	n	N	Enrich direct
map04075	Plant hormone signal transduction	2.91E-06	0.000265	40	625	890	30806	Over
map04076	Circadian rhythm - plant	3.68E-05	0.003345	16	170	890	30806	Over
map04077	Pentose and glucuronate interconversions	0.000244	0.022212	17	220	890	30806	Over

Supplementary Table 15. GO term enrichment of unique gene families in Lauraceae.

GO ID	GO term	GO class	*P* value	Adjusted *P* value	x1	x2	n	N	GO level
GO:0006355	DNA-templated regulation of transcription	BP	4.36E-19	3.05E-16	80	897	890	30806	Over
GO:0019219	Regulation of nucleobase-containing compound metabolic process	BP	4.35E-18	3.05E-15	82	972	890	30806	Over
GO:0031323	Regulation of cellular metabolic process	BP	1.45E-17	1.01E-14	83	1012	890	30806	Over
GO:0006351	DNA-templated transcription	BP	9.95E-17	6.96E-14	81	1005	890	30806	Over
GO:0050794	Regulation of cellular process	BP	5.23E-14	3.66E-11	96	1460	890	30806	Over
GO:0003700	Sequence-specific DNA binding transcription factor activity	MF	4.08E-13	2.85E-10	50	535	890	30806	Over
GO:0016070	RNA metabolic process	BP	2.84E-12	1.99E-09	85	1306	890	30806	Over
GO:0065007	Biological regulation	BP	3.62E-12	2.54E-09	98	1618	890	30806	Over
GO:0003677	DNA binding	MF	1.39E-09	9.75E-07	69	1095	890	30806	Over
GO:0090304	Nucleic acid metabolic process	BP	1.87E-08	1.31E-05	88	1641	890	30806	Over
GO:0006139	Nucleobase-containing compound metabolic process	BP	4.29E-08	3.00E-05	101	2012	890	30806	Over
GO:0046983	Protein dimerization activity	MF	1.02E-07	7.11E-05	28	307	890	30806	Over
GO:0034645	Cellular macromolecule biosynthetic process	BP	1.29E-07	9.03E-05	90	1766	890	30806	Over
GO:0034641	Cellular nitrogen compound metabolic process	BP	4.08E-07	0.000285	102	2137	890	30806	Over
GO:0006725	Cellular aromatic compound metabolic process	BP	4.26E-07	0.000298	102	2139	890	30806	Over
GO:0046483	Heterocycle metabolic process	BP	5.17E-07	0.000362	102	2148	890	30806	Over
GO:1901360	Organic cyclic compound metabolic process	BP	1.12E-06	0.000785	102	2185	890	30806	Over
GO:0003682	Chromatin binding	MF	6.38E-06	0.004463	24	296	890	30806	Over
GO:0006807	Nitrogen compound metabolic process	BP	1.91E-05	0.013385	105	2421	890	30806	Over

Supplementary Table 16. KEGG pathway enrichment of unique gene families in *L. cubeba*.

Map ID	Map title	*P* value	Adjusted *P* value	x	y	n	N	Enrich direct
map01062	Biosynthesis of terpenoids and steroids	1.15E-05	0.001272	7	16	1703	30806	Over
map00910	Nitrogen metabolism	0.000239	0.026516	12	66	1703	30806	Over

Supplementary Table 17. GO term enrichment of unique gene families in *L. cubeba*.

GO ID	GO term	GO class	*P* value	Adjusted *P* value	x1	x2	n	N	GO level
GO:0045261	Proton-transporting ATP synthase complex, catalytic core F(1)	CC	1.21E-12	8.66E-10	14	23	1703	30806	Over
GO:0051052	Regulation of DNA metabolic process	BP	2.45E-12	1.76E-09	15	28	1703	30806	Over
GO:0046961	Proton-transporting ATPase activity, rotational mechanism	MF	4.55E-11	3.27E-08	14	28	1703	30806	Over
GO:0046933	Proton-transporting ATP synthase activity, rotational mechanism	MF	1.49E-10	1.07E-07	14	30	1703	30806	Over
GO:0015986	ATP synthesis coupled proton transport	BP	1.83E-09	1.31E-06	14	35	1703	30806	Over
GO:0044030	Regulation of DNA methylation	BP	3.50E-09	2.51E-06	8	10	1703	30806	Over
GO:0016469	Proton-transporting two-sector ATPase complex	CC	1.55E-08	1.11E-05	17	60	1703	30806	Over
GO:1902600	Hydrogen ion transmembrane transport	BP	3.46E-08	2.49E-05	17	63	1703	30806	Over
GO:0003918	DNA topoisomerase type II (ATP-hydrolyzing) activity	MF	9.10E-08	6.53E-05	10	22	1703	30806	Over
GO:0006265	DNA topological change	BP	6.10E-07	0.000438	10	26	1703	30806	Over
GO:0015078	Hydrogen ion transmembrane transporter activity	MF	8.62E-07	0.000619	22	122	1703	30806	Over
GO:0009538	Photosystem I reaction center	CC	9.20E-07	0.000661	10	27	1703	30806	Over
GO:0009165	Nucleotide biosynthetic process	BP	1.30E-06	0.000931	19	97	1703	30806	Over
GO:0090407	Organophosphate biosynthetic process	BP	3.02E-06	0.002171	26	172	1703	30806	Over
GO:0030337	DNA polymerase processivity factor activity	MF	6.77E-06	0.004859	7	15	1703	30806	Over
GO:0043626	PCNA complex	CC	6.77E-06	0.004859	7	15	1703	30806	Over
GO:0006275	Regulation of DNA replication	BP	1.85E-05	0.013314	7	17	1703	30806	Over
GO:0009521	Photosystem	CC	4.72E-05	0.033874	14	74	1703	30806	Over
GO:0015077	Monovalent inorganic cation transmembrane transporter activity	MF	5.70E-05	0.040959	25	191	1703	30806	Over

Supplementary Table 18. KEGG enrichment analysis of significantly expanded gene families in *L. cubeba.*

Map ID	Map title	*P* value	Adjusted *P* value	x	y	n	N
map04144	Endocytosis	4.88E-54	2.34E-52	87	573	556	30806
map00920	Sulfur metabolism	3.34E-23	1.60E-21	22	61	556	30806
map00604	Glycosphingolipid biosynthesis - ganglio series	4.33E-18	2.08E-16	19	66	556	30806
map00600	Sphingolipid metabolism	7.79E-18	3.74E-16	25	141	556	30806
map02010	ABC transporters	1.32E-17	6.33E-16	25	144	556	30806
map00531	Glycosaminoglycan degradation	2.99E-15	1.44E-13	19	91	556	30806
map00511	Other glycan degradation	1.65E-11	7.93E-10	19	144	556	30806
map00270	Cysteine and methionine metabolism	2.23E-11	1.07E-09	22	203	556	30806
map00052	Galactose metabolism	1.19E-10	5.70E-09	19	161	556	30806
map01040	Biosynthesis of unsaturated fatty acids	3.50E-09	1.68E-07	11	55	556	30806
map00061	Fatty acid biosynthesis	3.39E-07	1.63E-05	11	84	556	30806
map04550	Signaling pathways regulating pluripotency of stem cells	9.24E-07	4.44E-05	9	58	556	30806
map00190	Oxidative phosphorylation	1.80E-06	8.65E-05	17	238	556	30806
map00196	Photosynthesis - antenna proteins	5.70E-06	0.000273	6	26	556	30806
map01212	Fatty acid metabolism	8.99E-06	0.000432	13	163	556	30806
map04141	Protein processing in endoplasmic reticulum	9.80E-06	0.00047	24	484	556	30806
map04540	Gap junction	2.55E-05	0.001225	9	86	556	30806
map04713	Circadian entrainment	2.55E-05	0.001225	9	86	556	30806
map00950	Isoquinoline alkaloid biosynthesis	3.02E-05	0.00145	12	157	556	30806
map04626	Plant-pathogen interaction	3.28E-05	0.001572	30	733	556	30806
map01230	Biosynthesis of amino acids	7.34E-05	0.003524	22	480	556	30806
map03040	Spliceosome	8.39E-05	0.004027	20	417	556	30806
map00350	Tyrosine metabolism	0.000155	0.007421	12	186	556	30806
map04510	Focal adhesion	0.000299	0.01433	9	118	556	30806
map04520	Adherens junction	0.00036	0.017267	9	121	556	30806
map00627	Aminobenzoate degradation	0.000638	0.030643	3	10	556	30806

Supplementary Table 19. GO enrichment analysis of the significantly expanded gene families in *L. cubeba*.

GO ID	GO term	GO class	*P* value	Adjusted *P* value	x1	x2	n	N
GO:0043086	Negative regulation of catalytic activity	BP	4.34E-75	1.02E-72	55	85	556	30806
GO:0042802	Identical protein binding	MF	1.35E-72	3.15E-70	55	91	556	30806
GO:0004252	Serine-type endopeptidase activity	MF	1.02E-71	2.38E-69	67	170	556	30806
GO:0042626	ATPase activity, coupled to transmembrane movement of substances	MF	4.67E-65	1.09E-62	64	180	556	30806
GO:0003824	Catalytic activity	MF	3.15E-64	7.37E-62	336	8184	556	30806
GO:0016787	Hydrolase activity	MF	5.74E-60	1.34E-57	185	2723	556	30806
GO:0004175	Endopeptidase activity	MF	7.68E-52	1.80E-49	70	350	556	30806
GO:0019829	Cation-transporting ATPase activity	MF	5.65E-38	1.32E-35	39	121	556	30806
GO:0005515	Protein binding	MF	1.71E-37	4.00E-35	176	3553	556	30806
GO:0000166	Nucleotide binding	MF	2.55E-36	5.96E-34	156	2932	556	30806
GO:0006508	Proteolysis	BP	3.50E-35	8.19E-33	70	613	556	30806
GO:0017111	Nucleoside-triphosphatase activity	MF	1.65E-34	3.85E-32	83	908	556	30806
GO:0005488	binding	MF	1.85E-34	4.33E-32	317	9868	556	30806
GO:0019538	Protein metabolic process	BP	1.62E-31	3.78E-29	146	2877	556	30806
GO:0016459	Myosin complex	CC	4.55E-30	1.06E-27	19	23	556	30806
GO:0016021	Integral component of membrane	CC	7.60E-30	1.78E-27	75	860	556	30806
GO:0005524	ATP binding	MF	5.03E-29	1.18E-26	120	2160	556	30806
GO:0022857	Transmembrane transporter activity	MF	2.00E-28	4.68E-26	65	673	556	30806
GO:0044765	Single-organism transport	BP	1.93E-26	4.51E-24	80	1103	556	30806
GO:0005215	Transporter activity	MF	6.89E-25	1.61E-22	69	876	556	30806
GO:0004713	Protein tyrosine kinase activity	MF	5.06E-24	1.18E-21	74	1038	556	30806
GO:0043168	Anion binding	MF	4.68E-23	1.10E-20	133	2993	556	30806
GO:0008324	Cation transmembrane transporter activity	MF	1.20E-20	2.80E-18	40	346	556	30806
GO:0006468	Protein phosphorylation	BP	1.88E-20	4.40E-18	76	1253	556	30806
GO:0004672	Protein kinase activity	MF	2.73E-20	6.38E-18	76	1261	556	30806
GO:0044238	Primary metabolic process	BP	4.68E-19	1.09E-16	201	6179	556	30806
GO:1901363	Heterocyclic compound binding	MF	1.09E-18	2.55E-16	179	5253	556	30806
GO:0097159	Organic cyclic compound binding	MF	1.14E-18	2.66E-16	179	5255	556	30806
GO:0043167	Ion binding	MF	3.18E-18	7.44E-16	176	5177	556	30806
GO:0045300	Acyl-[acyl-carrier-protein] desaturase activity	MF	4.53E-18	1.06E-15	11	13	556	30806
GO:0006812	Cation transport	BP	1.25E-17	2.92E-15	40	420	556	30806
GO:0071704	Organic substance metabolic process	BP	4.33E-17	1.01E-14	201	6431	556	30806
GO:0022892	Substrate-specific transporter activity	MF	3.14E-16	7.34E-14	44	557	556	30806
GO:0008152	Metabolic process	BP	5.98E-16	1.40E-13	243	8588	556	30806
GO:0016740	Transferase activity	MF	8.77E-16	2.05E-13	114	2890	556	30806
GO:0003774	Motor activity	MF	2.41E-15	5.64E-13	19	90	556	30806

GO:0016020	Membrane	CC	8.54E-15	2.00E-12	93	2187	556	30806
GO:0005516	Calmodulin binding	MF	8.02E-12	1.88E-09	9	18	556	30806
GO:0004553	Hydrolase activity, hydrolyzing O-glycosyl compounds	MF	2.37E-11	5.54E-09	32	434	556	30806
GO:0004650	Polygalacturonase activity	MF	1.60E-10	3.75E-08	13	66	556	30806
GO:0016831	Carboxy-lyase activity	MF	3.59E-10	8.40E-08	12	57	556	30806
GO:0055085	Transmembrane transport	BP	2.10E-09	4.91E-07	37	667	556	30806
GO:0044699	Single-organism process	BP	7.78E-09	1.82E-06	135	4656	556	30806
GO:0030246	Carbohydrate binding	MF	3.00E-08	7.01E-06	15	137	556	30806
GO:0003779	Actin binding	MF	4.78E-08	1.12E-05	10	55	556	30806
GO:0030036	Actin cytoskeleton organization	BP	4.78E-08	1.12E-05	10	55	556	30806
GO:0045735	Nutrient reservoir activity	MF	8.12E-08	1.90E-05	10	58	556	30806
GO:0006606	Protein import into nucleus	BP	1.05E-07	2.46E-05	4	4	556	30806
GO:0030170	Pyridoxal phosphate binding	MF	1.17E-07	2.75E-05	12	93	556	30806
GO:0016758	Transferase activity, transferring hexosyl groups	MF	2.75E-07	6.43E-05	25	423	556	30806
GO:0048544	Recognition of pollen	BP	6.89E-07	0.000161	11	90	556	30806
GO:0009765	Photosynthesis, light harvesting	BP	1.45E-06	0.000339	6	21	556	30806
GO:0006631	Fatty acid metabolism	BP	8.54E-06	0.002	11	116	556	30806
GO:0006979	Response to oxidative stress	BP	1.09E-05	0.002555	11	119	556	30806
GO:0019752	Carboxylic acid metabolism	BP	1.10E-05	0.002577	23	455	556	30806
GO:0004601	Peroxidase activity	MF	1.88E-05	0.004396	11	126	556	30806
GO:0005975	Carbohydrate metabolism	BP	2.31E-05	0.005407	32	792	556	30806
GO:0044763	Single-organism cellular process	BP	4.69E-05	0.010973	81	2890	556	30806

Supplementary Table 20. KEGG enrichment analysis of significantly contracted gene families in *L. cubeba.*

Map ID	Map title	*P* value	Adjusted *P* value	x	y	n	N	Enrich direct
map04510	Focal adhesion	2.19E-07	6.56E-07	3	118	4	30806	Over
map03015	mRNA surveillance pathway	2.98E-06	8.95E-06	3	281	4	30806	Over

Supplementary Table 21. GO enrichment analysis of significantly contracted gene families in *L. cubeba*.

GO ID	GO term	GO class	*P* value	Adjusted *P* value	x1	x2	n	N	GO level
GO:0016787	Hydrolase activity	MF	6.09E-05	0.001584	4	2723	4	30806	Over
GO:0009678	Hydrogen-translocating pyrophosphatase activity	MF	0.000649	0.016877	1	5	4	30806	Over
GO:0004427	Inorganic diphosphatase activity	MF	0.001557	0.04049	1	12	4	30806	Over

Supplementary Table 22. Number of paralogous genes located on synteny/collinear segments/anchors in *L. cubeba.*

Chromosomes	Genes	2 segments		3 segments		4 segments		5 segments	
		# anchors	% anchors	# anchors	% anchors	# anchors	% anchors	# anchors	% anchors
chr1	3709	844	22.76%	823	22.19%	607	16.37%	77	2.08%
chr2	4853	1477	30.43%	1191	24.54%	976	20.11%	211	4.35%
chr3	2684	515	19.19%	741	27.61%	311	11.59%	28	1.04%
chr4	3673	1038	28.26%	896	24.39%	723	19.68%	102	2.78%
chr5	3438	954	27.75%	842	24.49%	646	18.79%	111	3.23%
chr6	2186	436	19.95%	460	21.04%	478	21.87%	60	2.74%
chr7	2022	671	33.18%	285	14.09%	243	12.02%	36	1.78%
chr8	2052	583	28.41%	511	24.90%	107	5.21%	0	0%
chr9	1447	265	18.31%	618	42.71%	199	13.75%	0	0%
chr10	1647	381	23.13%	350	21.25%	236	14.33%	23	1.40%
chr11	1774	284	16.01%	388	21.87%	467	26.32%	89	5.02%
chr12	829	183	22.07%	135	16.28%	76	9.17%	0	0%
Total	30314	7631	25.17%	7240	23.88%	5069	16.72%	737	2.43%

Supplementary Table 23. Sample information for Illumina transcriptome sequences of 23 species representing 16 genera.

Family	Genus	Species	Male/female	Mix of tissues*
Lauraceae	*Litsea*	*Litsea rubescens*	Female flower	a,b,c,d,e
		Litsea rubescens	Male flower	a,b,c,d,e
		Litsea tsinlingensis	Female flower	a,b,c,d,e,f
		Litsea tsinlingensis	Male flower	a,b,d,e,f
		Litsea cubeba	Female flower	a,b,c,d,e,f
		Litsea cubeba	Male flower	a,b,c,d,e,f
	Lindera	*Lindera megaphylla*	Female flower	a,b,c,d,e
		Lindera megaphylla	Male flower	a,b,c,d,e
	Laurus	*Laurus nobilis*	Female flower	a,b,c,d,e,f
		Laurus nobilis	Male flower	a,b,c,d,e,f
	Sassafras	*Sassafras tzumu*	Female flower	a,b,c,d
		Sassafras tzumu	Male flower	a,b,c,d
	Cinnamomum	*Cinnamomum verum*	Bisexual flower	a,b,c,d,e,f
		Cinnamomum tenuipile	Bisexual flower	a,b,c,d,e,f
		Cinnamomum burmanni	Bisexual flower	a,b,c,d,e,f
	Phoebe	*Phoebe sheareri*	Bisexual flower	a,b,c,d
		Phoebe hunanensis	Bisexual flower	a,b,c,d
		Phoebe tavoyana	Bisexual flower	a,b,c,d,e,f
	Nothaphoebe	*Nothaphoebe cavaleriei*	Bisexual flower	a,b,c,d,e,f
	Dehaasia	*Dehaasia hainanensis*	Bisexual flower	c,d,e,f
	Alseodaphne	*Alseodaphne petiolaris*	Bisexual flower	c,d,e,f
	Machilus	*Machilus salicina*	Bisexual flower	a,b,c,d,e,f
	Persea	*Persea americana*	Bisexual flower	a,b,c,d,e,f
	Beilschmiedia	*Beilschmiedia intermedia*	Bisexual flower	c,d,e,f
		Beilschmiedia percoriacea	Bisexual flower	c,d,e,f
	Cryptocarya	*Cryptocarya brachythyrsa*	Bisexual flower	a,b,c,d,e,f
	Caryodaphnopsis	*Caryodaphnopsis tonkinensis*	Bisexual flower	a,b,c,d,f
	Cassytha	*Cassytha filiformis*	Bisexual flower	a,c,d,e
Calycanthaceae	*Chimonanthus*	*Chimonanthus praecox*	Bisexual flower	a,b,c,d,e,f

* a, b, c, d, e, and f denote flower buds, flowers, leaves, stems, buds, and bark, respectively.

Supplementary Table 24. Sample information for low-coverage genome data of 47 species in 20 genera of Lauraceae.

Family	Genus	Species	Male/female
Lauraceae	*Alseodaphne*	*Alseodaphne hainanensis*	30X
		Alseodaphne petiolaris	30X
	Machilus	*Machilus salicina*	30X
	Nothaphoebe	*Nothaphoebe cavaleriei*	30X
	Persea	*Persea americana*	30X
	Phoebe	*Phoebe sheareri*	30X
		Phoebe hunanensis	30X
		Phoebe tavoyana	30X
	Beilschmiedia	*Beilschmiedia intermedia*	30X
		Beilschmiedia percoriacea	30X
	Dehaasia	*Dehaasia hainanensis*	30X
	Syndiclis	*Syndiclis chinensis*	30X
	Caryodaphnopsis	*Caryodaphnopsis tonkinensis*	30X
	Cinnamomum	*Cinnamomum verum*	30X
		Cinnamomum tenuipile	30X
		Cinnamomum burmanni	30X
	Neocinnamomum	*Neocinnamomum delavayi*	30X
	Actinodaphne	*Actinodaphne lecomtei*	30X
	Neolitsea	*Neolitsea sericea*	30X
	Sassafras	*Sassafras tzumu*	30X
	Laurus	*Laurus nobilis*	30X
	Lindera	*Lindera megaphylla*	30X
	Cryptocarya	*Cryptocarya brachythyrsa*	30X
	Litsea	*Litsea auriculata*	30X
		Litsea chunii	15X
		Litsea coreana var. lanuginosa	15X
		Litsea coreana var. sinensis	15X
		Litsea cubeba	30X
		Litsea dilleniifolia	30X
		Litsea elongata	15X
		Litsea euosma	15X
		Litsea foveolata	15X
		Litsea garrettii	15X
		Litsea glutinosa	15X
		Litsea ichangensis	15X
		Litsea mollis	15X
		Litsea moupinensis	15X
		Litsea moupinensis var. szechuanica	15X
		Litsea pierrei	30X
		Litsea populifolia	15X
		Litsea pungens	15X
		Litsea rubescens	30X
		Litsea sericea	15X
		Litsea tsinlingensis	30X
		Litsea veitchiana	15X
	Cassytha	*Cassytha filiformis*	30X
Calycanthaceae	*Chimonanthus*	*Chimonanthus praecox*	30X

Supplementary Table 25. Low coverage genome sequencing data of species in Lauraceae.

Sample	Mapped reads	Total reads	Mapping rate (%)	Average depth (X)	Coverage at least 1X (%)	Coverage at least 4X (%)
Alseodaphne petiolaris	272501852	313054864	87.05	46.67	50.53	40.07
Sassafras tzumu (M)	319774769	364786294	87.66	54.28	51.11	41.27
Sassafras tzumu (F)	368398302	413672626	89.06	58.1	51.49	42.13
Chimonanthus praecox	94268576	353833062	26.64	50.91	11.2	3.72
Cryptocarya brachythyrsa	87053318	364773164	23.87	38.24	13.85	4.98
Cryptocarya brachythyrsa	66228859	311320044	21.27	29.63	10.49	3.95
Persea americana	324188712	375545574	86.32	53.19	46.62	38.69
Lindera megaphylla (M)	344534098	419360896	82.16	50.91	60.69	49.05
Lindera megaphylla (F)	318043507	345487394	92.06	47.4	60.12	48.55
Litsea rubescens (M)	348900845	370169118	94.25	50.81	58.45	47.38
Litsea rubescens (F)	377865717	403098952	93.74	51.61	57.97	46.84
Beilschmiedia percoriacea	69620489	349990106	19.89	37.76	16.93	4.23
Dehaasia hainanensis	297094499	366089330	81.15	54.2	45.74	37.68
Machilus salicina	344675493	390678134	88.22	52.57	48.22	39.46
Caryodaphnopsis tonkinensis	117100979	390278372	30	48.8	15.37	7.96
Litsea cubeba (M)*2*	544935460	553092328	98.53	53.52	93.98	90.17
Litsea cubeba (M)*3*	479918532	487199966	98.51	45.99	93.56	89.33
Litsea cubeba (F)*4*	575312064	584789226	98.38	55.48	95.13	92.3
Litsea cubeba (F)*5*	729408705	738597592	98.76	70.14	94.41	91.58
Litsea cubeba (F)*6*	603051712	611144078	98.68	54.24	98.93	98.1
Actinodaphne lecomtei	324922193	353517204	91.91	48.41	58.93	48.17
Litsea tsinlingensis (M)	391065660	416672580	93.85	56.88	59.29	48.49
Litsea tsinlingensis (F)	349350581	372075264	93.89	53.02	58.31	47.65
Beilschmiedia intermedia	78298881	323991818	24.17	38.39	10.02	4.22
Nothaphoebe cavaleriei	266289243	412975306	64.48	45.4	48.16	38.93
Cassytha filiformis	42540706	399096908	10.66	21.97	9.75	2.55
Phoebe tavoyana	313389731	362971134	86.34	52.88	48.32	39.94
Cinnamomum verum	322074465	392765320	82	51.08	49.63	40.14
Cinnamomum tenuipile	332673039	373735698	89.01	57.36	47.31	39.68
Phoebe hunanensis	459283912	529725560	86.7	68.99	49.94	41.46
Neocinnamomum delavayi	330722279	368380590	89.78	48.04	59.25	48.35
Syndiclis chinensis	107331782	383799978	27.97	45.49	12.02	4.96
Alseodaphne hainanensis	346550379	389422940	88.99	58.42	49.18	40.54
Cinnamomum burmanni	314524694	368431642	85.37	50.94	49.64	40.36
Phoebe formosana	291993056	333677782	87.51	48.74	47.98	39.31
Neolitsea sericea	456194435	505623272	90.22	60.49	60.79	50.11
Laurus nobilis (M)	324930062	380959338	85.29	45.43	55.84	44.61
Laurus nobilis (F)	247406411	290614132	85.13	37.42	54.38	42.68

Supplementary Table 26. Sample information for the transcriptome data of flower buds for 21 species in 13 genera.

Family	Genus	Species	Male/ female	Sample information
Lauraceae	*Machilus*	*Machilus salicina*	Bisexual flower	Triplicates
	Persea	*Persea americana*	Bisexual flower	Triplicates
	Phoebe	*Phoebe sheareri*	Bisexual flower	Triplicates
		Phoebe hunanensis	Bisexual flower	Triplicates
		Phoebe tavoyana	Bisexual flower	Triplicates
	Beilschmiedia	*Beilschmiedia intermedia*	Bisexual flower	Two repetition
	Caryodaphnopsis	*Caryodaphnopsis tonkinensis*	Bisexual flower	Single sample
	Cinnamomum	*Cinnamomum verum*	Bisexual flower	Triplicates
		Cinnamomum tenuipilum	Bisexual flower	Triplicates
		Cinnamomum burmanni	Bisexual flower	Triplicates
	Sassafras	*Sassafras tzumu*	Male flower	Triplicates
			Female flower	Triplicates
	Litsea	*Litsea rubescens*	Male flower	Triplicates
			Female flower	Triplicates
		Litsea tsinlingensis	Male flower	Triplicates
			Female flower	Triplicates
		Litsea cubeba	Male flower	Triplicates
			Female flower	Triplicates
		Litsea mollis	Male flower	Triplicates
			Female flower	Triplicates
		Litsea euosma	Male flower	Triplicates
			Female flower	Triplicates
	Laurus	*Laurus nobilis*	Female flower	Two repetition
			Male flower	Triplicates
	Lindera	*Lindera megaphylla*	Male flower	Triplicates
			Female flower	Triplicates
	Cryptocarya	*Cryptocarya brachythyrsa*	Bisexual flower	Two repetition
	Cassytha	*Cassytha filiformis*	Bisexual flower	Single sample
Calycanthaceae	*Chimonanthus*	*Chimonanthus praecox*	Bisexual flower	Triplicates

Supplementary Table 27. MADS-box genes in 9 species.

Category	*A. trichopod*[10]	*N. colorata*[35]	*L. cubeba*	*C. kanehirae*[36]	*M. cordata*[37]	Poplar[38]	*Arabidopsis*[39]	Rice[40]	*Apostasia*[41]
Type II (Total)	23	25	46	43	26	64	45	44	27
$MIKC^c$	21	23	41	37	22	55	39	39	25
$MIKC^*$	2	2	5	6	4	9	6	5	2
Type I (Total)	13	36	18	21	24	41	61	31	9
Mα	6	28	9	16	18	23	25	12	5
Mβ	6	4	6	3	3	12	20	9	0
Mγ	1	4	3	2	3	6	16	10	4
Total	36	61	64	64	50	105	106	75	36

Supplementary Table 28. List of 67 MADS-box genes identified in *L. cubeba*.

Gene ID	Name	ORF (bp)	Protein length (aa)	Type	Subfamily	Pseudogene
Lcu01G_01070	*LcMADS1*	723	240	MIKCc	A	
Lcu02G_05242	*LcMADS2*	678	225	MIKCc	A	
Lcu05G_17982	*LcMADS3*	558	185	MIKCc	B-AP3	
Lcu05G_17983	*LcMADS4*	678	225	MIKCc	B-AP3	
Lcu11G_28071	*LcMADS5*	630	209	MIKCc	B-PI	
Lcu11G_29146	*LcMADS6*	636	211	MIKCc	B-PI	
Lcu11G_28191	*LcMADS7*	672	223	MIKCc	C/D	
Lcu06G_19364	*LcMADS8*	663	220	MIKCc	C/D	
Lcu08G_23809	*LcMADS9*	792	263	MIKCc	C/D	
Lcu09G_26065	*LcMADS10*	576	191	MIKCc	C/D	
Lcu09G_24881	*LcMADS11*	672	223	MIKCc	C/D	
Lcu01G_03052	*LcMADS12*	714	237	MIKCc	E	
Lcu04G_12198	*LcMADS13*	186	61	MIKCc	E	
Lcu01G_01071	*LcMADS14*	726	241	MIKCc	E	
Lcu02G_05235	*LcMADS15*	432	143	MIKCc	E	
Lcu03G_09993	*LcMADS16*	186	61	MIKCc	E	
Lcu02G_05240	*LcMADS17*	735	244	MIKCc	E	
Lcu08G_24040	*LcMADS18*	726	241	MIKCc	AGL6	
Lcu04G_13964	*LcMADS19*	723	240	MIKCc	AGL6	
Lcu05G_18186	*LcMADS20*	696	231	MIKCc	Bs	
Lcu08G_24039	*LcMADS21*	681	226	MIKCc	SOC1	
Lcu04G_13962	*LcMADS22*	648	215	MIKCc	SOC1	
Lcu03G_10965	*LcMADS23*	291	96	MIKCc	SOC1	
Lcu03G_10966	*LcMADS24*	705	234	MIKCc	SOC1	
Lcu03G_10970	*LcMADS25*	276	91	MIKCc	SOC1	
Lcu03G_10967	*LcMADS26*	327	108	MIKCc	SOC1	
Lcu03G_10982	*LcMADS27*	183	60	MIKCc	SOC1	
Lcu02G_07456	*LcMADS28*	705	234	MIKCc	SVP	
Lcu06G_18866	*LcMADS29*	477	158	MIKCc	SVP	
Lcu06G_18870	*LcMADS30*	705	234	MIKCc	SVP	
Lcu12G_30131	*LcMADS31*	864	287	MIKCc	SVP	
Lcu12G_30161	*LcMADS32*	1113	370	MIKCc	SVP	
Lcu02G_08080	*LcMADS33*	303	100	MIKCc	AGL12	
Lcu11G_28189	*LcMADS34*	1740	579	MIKCc	AGL12	
Lcu11G_28984	*LcMADS35*	768	255	MIKCc	AGL15	
Lcu01G_02553	*LcMADS36*	663	220	MIKCc	ANR1	
Lcu02G_05078	*LcMADS37*	708	235	MIKCc	ANR1	
Lcu05G_16289	*LcMADS38*	759	252	MIKCc	ANR1	
Lcu05G_16323	*LcMADS39*	708	235	MIKCc	ANR1	
Lcu03G_09280	*LcMADS40*	741	246	MIKCc	TM8	
Lcu06G_18746	*LcMADS41*	933	310	MIKC*	S	
Lcu04G_11551	*LcMADS42*	1002	333	MIKC*	S	
Lcu07G_21871	*LcMADS43*	219	72	MIKC*	P	
Lcu01G_03277	*LcMADS44*	186	61	MIKC*	P	
Lcu10G_26692	*LcMADS45*	1101	366	MIKC*	P	
Lcu03G_11038	*LcMADS46*	1134	377	Type I	Mα	
Lcu04G_12093	*LcMADS47*	342	113	Type I	Mα	
Lcu04G_12027	*LcMADS48*	414	137	Type I	Mα	
Lcu04G_12313	*LcMADS49*	921	306	Type I	Mα	
Lcu04G_12325	*LcMADS50*	1092	363	Type I	Mα	
Lcu01G_02503	*LcMADS51*	768	255	Type I	Mα	
Lcu03G_10324	*LcMADS52*	654	217	Type I	Mα	
Lcu03G_08749	*LcMADS53*	783	260	Type I	M	
Lcu03G_08750	*LcMADS54*	840	279	Type I	M	
Lcu11G_28101	*LcMADS55*	687	228	Type I	Mγ	
Lcu11G_28100	*LcMADS56*	687	228	Type I	Mγ	
Lcu07G_20672	*LcMADS57*	720	239	Type I	Mγ	

Lcu06G_19370	*LcMADS58*	678	225	MIKCc	A	
Lcu04G_12197	*LcMADS59*	369	122	MIKCc	E	v
Lcu03G_10964	*LcMADS60*	462	153	MIKCc	SOC1	v
Lcu02G_05236	*LcMADS61*	165	54	MIKCc	E	v
Lcu05G_18358	*LcMADS62*	963	320	Type I	Mβ	
Lcu05G_18359	*LcMADS63*	1044	347	Type I	Mβ	
Lcu08G_24618	*LcMADS64*	966	321	Type I	Mβ	
Lcu08G_24619	*LcMADS65*	966	321	Type I	Mβ	
Lcu07G_22566	*LcMADS66*	1020	339	Type I	Mβ	
Lcu02G_08563	*LcMADS67*	1296	431	Type I	Mβ	

Supplementary Table 29. Components in *L. cubeba* essential oil.

Compound	1*	2	3	4	5	6	7	8
Geranial	3.13	4.58	8.61	10.71	29.79	41.69	47.93	32.6
Neral	2.76	4.01	7.55	9.40	26.01	36.13	39.69	28.98
Limonene	45.77	61.23	47.14	47.17	17.17	3.59	0.07	15.34
Linalool	1.05	2.00	3.27	3.64	4.08	2.89	2.15	2.59
Eucalyptol	2.13	0.00	2.01	3.08	2.02	0.87	0.05	2.52
5-hepten-2-one, 6-methyl-	0.61	1.10	1.79	2.47	2.59	1.04	0.35	1.57
β-pinene	9.94	6.34	4.14	4.17	1.14	0.13	0.00	1.56
2-cyclohexen-1-one, 4-(2-oxopropyl)-	0.12	0.19	0.35	0.31	0.92	1.22	1.06	1.22
Camphene	9.47	3.84	2.38	2.39	0.6	0.06	0.00	1.00
2-propenoic acid, 2-methyl-, decyl ester	0.00	0.01	0.12	0.21	0.77	0.94	1.59	0.99
1R-α-Pinene	8.64	4.23	2.31	2.2	0.44	0.00	0.00	0.92
Tricyclo[4.3.1.1(3,8)] undecan-1-ol	0.41	0.73	1.19	1.43	1.25	0.69	0.23	0.83
o-mentha-1(7),8-dien -3-ol	0.13	0.18	0.33	0.27	0.75	0.70	0.55	0.83
α-terpineol	0.68	1.16	1.68	0.93	0.96	0.66	0.61	0.81
β-phellandrene	2.06	2.37	1.57	2.04	0.85	0.1	0.00	0.61
Caryophyllene	1.47	0.83	0.41	0.31	0.00	0.51	0.00	0.57
Limonene oxide, trans-	0.24	0.34	0.73	0.57	0.59	0.78	0.26	0.52
Citronellal	0.17	0.13	0.21	0.19	0.51	0.53	0.41	0.51
Borneol	1.18	0.92	1.79	0.82	1.3	0.38	0.72	0.49
(-)-myrtenol	0.18	0.22	0.61	0.43	0.46	0.64	0.34	0.39
Nerol	0.18	0.36	0.78	0.48	0.7	0.36	0.31	0.34
Caryophyllene oxide	1.36	0.00	1.63	0.8	0.91	0.36	0.53	0.32
cis-carveol	0.23	0.44	1.24	0.66	0.51	0.46	0.32	0.32
bornyl acetate	0.02	0.1	0.04	0.02	0.19	0.18	0.16	0.3
cis-linaloloxide	0.00	0.00	0.00	0.00	0.00	0.44	0.11	0.24
trans-p-mentha-2,8-di enol	0.11	0.23	0.56	0.36	0.27	0.27	0.12	0.21
2,4-heptadiene, 2,4-dimethyl-	0.03	0.05	0.19	0.09	0.17	0.32	0.09	0.19
Geraniol	0.05	0.08	0.15	0.08	0.69	0.09	0.18	0.17
Camphene hydrate	0.05	0.06	0.04	0.07	0.12	0.12	0.08	0.16
Phenol, 2,3,5,6-tetramethyl-	0.35	0.49	0.85	1.17	0.51	0.09	0.00	0.15
Furan, 2,3-dihydro-5-methyl -	0.07	0.26	0.54	0.24	0.2	0.2	0.13	0.14
Campholenic aldehyde	0.09	0.05	0.17	0.09	0.19	0.21	0.10	0.14
Hexanoic acid, 3-hexenyl ester, (Z)-	0.08	0.00	0.00	0.13	0.18	0.00	0.00	0.13
trans-2-caren-4-ol	0.00	0.00	0.00	0.00	0.08	0.05	0.05	0.12
Benzene, 1-methyl-2-(1-methyl ethyl)-	0.25	0.00	0.59	0.18	0.08	0.06	0.00	0.12
Geranic acid	0.05	0.03	0.61	0.07	0.26	0.21	0.61	0.09

cis-verbenol	0.08	0.08	0.23	0.18	0.16	0.11	0.05	0.09
DL-camphor	0.05	0.04	0.09	0.06	0.08	0.03	0.00	0.07
Ethyl tiglate	0.02	0.00	0.01	0.02	0.03	0.04	0.00	0.07
5-hepten-1-ol, 2,6-dimethyl-	0.03	0.03	0.09	0.08	0.08	0.07	0.04	0.05
Methyl salicylate	0.02	0.05	0.12	0.08	0.04	0.08	0.00	0.04
Fenchol	0.03	0.02	0.05	0.03	0.05	0.00	0.00	0.03
Neric acid	0.01	0.01	0.18	0.02	0.06	0.07	0.13	0.03
α-phellandrene	0.08	0.14	0.1	0.04	0.00	0.00	0.00	0.03
Pina-2-ene-7-one	0.00	0.02	0.04	0.04	0.04	0.03	0.00	0.03
α-terpinene	0.06	0.06	0.04	0.03	0.02	0.00	0.00	0.03
(E)-β-ocimene	0.05	0.04	0.03	0.06	0.03	0.03	0.00	0.02
Perillic alcohol	0.02	0.03	0.09	0.05	0.04	0.04	0.04	0.02
Naphthalene, decahydro-	0.11	0.00	0.1	0.05	0.05	0.00	0.03	0.02
4-carene	0.02	0.03	0.02	0.04	0.02	0.00	0.00	0.02
α-cubebene	0.03	0.02	0.00	0.00	0.00	0.00	0.00	0.01

* Numbers 1-8 indicate the developmental stage of *L. cubeba* fruit.

Supplementary Table 30. List of TPSs numbers from transctiptome data for various tissues of species in Lauraceae.

Species	TPS Genes						
	TPS-a	TPS-b	TPS-c	TPS-e/f	TPS-g	TPS-x	Total
Litsea cubeba	17	24	1	6	3	1	52
Phoebe sheareri	19	8	0	5	4	0	36
Persea americana	6	17	2	8	2	0	35
Machilus salicina	11	13	0	4	4	0	32
Sassafras tzumu	13	3	0	11	3	0	30
Phoebe hunanensis	15	4	0	4	3	0	26
Laurus nobilis	8	4	1	10	3	0	26
Cinnamomum verum	6	12	1	2	3	0	24
Alseodaphne petiolaris	9	5	1	5	2	0	22
Beilschmiedia intermedia	1	9	1	7	3	0	21
Caryodaphnopsis tonkinensis	12	6	0	2	1	0	21
Litsea rubescens	3	4	1	5	4	1	18
Cassytha filiformis	7	3	0	4	2	0	16
Cryptocarya brachythyrsa	1	10	0	1	3	0	15
Beilschmiedia percoriacea	2	4	0	5	4	0	15
Cinnamomum tenuipilum	7	3	2	3	0	0	15
Cinnamomum burmanni	7	3	2	3	0	0	15
Litsea tsinlingensis	9	1	0	4	1	0	15
Lindera megaphylla	2	3	0	5	4	0	14
Dehaasia hainanensis	0	8	1	1	2	0	12

The various tissues included flower buds, flowers, leaves, stems, buds, and bark. Source data are provided as a Source Data file.

Supplementary Table 31. List of TPSs numbers from Illumina transctiptome data in flower buds in species of Lauraceae.

Species	TPS genes				
	TPS-a	TPS-b	TPS-c	TPS-e/f	TPS-g
Lindera megaphylla	4	4	1	11	3
Phoebe sheareri	4	4	0	2	0
Phoebe tavoyana	2	0	0	12	0
Beilschmiedia intermedia	1	6	1	2	0
Sassafras tzumu	1	1	1	10	2
Cinnamomum burmanni	1	4	1	3	1
Cinnamomum verum	0	1	2	0	7
Cinnamomum tenuipile	1	2	0	0	3
Litsea euosma	3	4	0	0	0
Litsea rubescens	4	13	0	6	2
Litsea tsinlingensis	0	9	2		0
Litsea mollis	6	4	0	10	2
Laurus nobilis	4	0	1	4	0
Cryptocarya brachythyrsa	1	0	1	2	0
Cassytha filiformis	2	0	0	1	1

Source data are provided as a Source Data file.

Supplementary Table 32. TPSs information for *L. cubeba*.

Chromosome location	Gene ID	Gene name		Scaffold location	Protein size	TPS subfamily
chr1	Lcu01G_01644	*LcuTPS19*	97327451	97333890	580	b
chr1	Lcu01G_02299	*LcuTPS51*	145986185	145996961	813	c
chr2	Lcu02G_05130	*LcuTPS41*	34356572	34364506	427	b
chr2	Lcu02G_05131	*LcuTPS40*	34417384	34430169	449	b
chr2	Lcu02G_05475	*LcuTPS8*	42944020	42954101	511	a
chr2	Lcu02G_05478	*LcuTPS7*	43059325	43114741	929	a
chr2	Lcu02G_05491	*LcuTPS35*	43577938	43588445	509	b
chr3	Lcu03G_10508	*LcuTPS16*	103992948	104005176	558	a
chr3	Lcu03G_10509	*LcuTPS15*	104113204	104126620	557	a
chr3	Lcu03G_10510	*LcuTPS17*	104248511	104258782	510	a
chr4	Lcu04G_12301	*LcuTPS28*	79172739	79182657	402	b
chr4	Lcu04G_12302	*LcuTPS27*	79259609	79301171	401	b
chr4	Lcu04G_13529	*LcuTPS45*	113871875	113877064	602	x
chr5	Lcu05G_15827	*LcuTPS24*	77836695	77844710	525	b
chr5	Lcu05G_15831	*LcuTPS25*	78229912	78238959	621	b
chr5	Lcu05G_16176	*LcuTPS4*	92305422	92311143	561	a
chr5	Lcu05G_16198	*LcuTPS20*	93255883	93263573	580	b
chr6	Lcu06G_19740	*LcuTPS5*	58030457	58032925	421	a
chr7	Lcu07G_21431	*LcuTPS2*	55814140	55815345	730	a
chr8	Lcu08G_22664	*LcuTPS48*	3878818	3889846	853	e/f
chr8	Lcu08G_22670	*LcuTPS50*	4056701	4070191	851	e/f
chr8	Lcu08G_22671	*LcuTPS49*	4164494	4173904	853	e/f
chr8	Lcu08G_22874	*LcuTPS43*	12171265	12174498	512	g
chr8	Lcu08G_22876	*LcuTPS44*	12284215	12287390	483	g
chr8	Lcu08G_22877	*LcuTPS39*	12299243	12390069	601	b
chr8	Lcu08G_22878	*LcuTPS30*	12499961	12531035	591	b
chr8	Lcu08G_22883	*LcuTPS32*	12720305	12763094	605	b
chr8	Lcu08G_22893	*LcuTPS6*	13084729	13165824	511	a
chr8	Lcu08G_22935	*LcuTPS52*	42926151	42957493	565	e/f
chr8	Lcu08G_23225	*LcuTPS31*	14070387	14085092	444	b
chr8	Lcu08G_23231	*LcuTPS34*	14292293	14302532	600	b
chr8	Lcu08G_23234	*LcuTPS38*	14501045	14518425	589	b
chr8	Lcu08G_23235	*LcuTPS33*	14557157	14576053	530	b
chr8	Lcu08G_23238	*LcuTPS37*	13864474	13874916	417	b
chr8	Lcu08G_23239	*LcuTPS29*	13951083	14007350	539	b
chr8	Lcu08G_24568	*LcuTPS36*	48859347	48864171	511	b
chr9	Lcu09G_25124	*LcuTPS47*	11809246	11817100	759	e/f
chr9	Lcu09G_25125	*LcuTPS46*	11841577	11849830	752	e/f
chr9	Lcu09G_26017	*LcuTPS26*	74917827	74925005	545	b
chr10	Lcu10G_27145	*LcuTPS42*	25836682	25845705	603	g
chr10	Lcu10G_27165	*LcuTPS21*	27932117	27957394	491	b
chr10	Lcu10G_27166	*LcuTPS18*	28375414	28381880	562	b
chr10	Lcu10G_27179	*LcuTPS22*	29054891	29065574	584	b
chr10	Lcu10G_27180	*LcuTPS23*	29189134	29200186	585	b
chr10	Lcu10G_27191	*LcuTPS1*	29594190	29599753	628	a
chr10	Lcu10G_27196	*LcuTPS3*	29950277	30023251	513	a
chr12	Lcu12G_30299	*LcuTPS10*	54103832	54116000	444	a
chr12	Lcu12G_30302	*LcuTPS14*	54493504	54504137	518	a
chr12	Lcu12G_30304	*LcuTPS13*	54586315	54598100	560	a
chr12	Lcu12G_30311	*LcuTPS12*	54916041	54934378	516	a
chr12	Lcu12G_30313	*LcuTPS9*	55225393	55242571	393	a
chr12	Lcu12G_30314	*LcuTPS11*	55309993	55322295	839	a

Supplementary Table 33. Quantity information for the TPS family of 10 species.

Species	Putative full length TPSs	TPS subfamily							
		a	b	c	d	e/f	g	h	x
Arabidopsis thaliana	32	22	6	1	0	2	1	0	0
Amborella trichopoda	13	0	6	1	0	2	4	0	0
Oryza sativa	30	17	0	1	0	10	2	0	0
Zea mays	29	16	2	3	0	5	3	0	0
Vitis vinifera	53	28	9	2	0	1	13	0	0
Physcomitrella patens	1	0	0	1	0	0	0	0	0
Liriodendron chinense	58	23	17	2	0	10	6	0	0
Selaginella moellendorffii	13	0	0	3	0	2	0	8	0
Cinnamomum kanehirae	79	28	41	5	0	4	0	0	1
Litsea cubeba	52	17	24	1	0	6	3	0	1
Gymnosperms	18	0	0	0	18	0	0	0	0

Supplementary Table 34. Enzyme annotations and mRNA-seq expression levels of *L. cubeba.*

Gene name		Gene name	
Acetyl-CoA: Acetyl-CoA C-acetyltransferase	*ACOT1*	Farnesyl diphosphate synthase	*FPPS1*
	ACOT2		*FPPS2*
	ACOT3		*FPPS3*
	ACOT4		*FPPS4*
	ACOT5	Geranylgeranyl diphosphate synthase	*GGPPS1*
4-(cytidine-5′-diphospho)-2-C-methylerythritol kinase	*CMK*		*GGPPS2*
2-C-methylerythritol-4-phosphate cytidyltransferase	*CMS*		*GGPPS3*
1-deoxyxylulose-5-phosphate reductoisomerase	*DXR*		*GGPPS3*
1-deoxyxylulose-5-phosphate synthase	*DXS1*		*GGPPS3*
	DXS2	Geranyl diphosphate synthase	*GPPS1*
	DXS3		*GPPS2*
	DXS4		*GPPS3*
	DXS5		*GPPS4*
	DXS6	Mevalonate-5-diphosphate decarboxylase	*MDC*
	DXS7	2-C-methylerythritol-2,4-cyclodi phosphate synthase	*MDS*
1-hydroxy-2-methyl-butenyl 4-diphosphate reductase	*HDR1*	Mevalonate kinase	*MK*
	HDR2	Phosphomevalonate kinase	*PMK*
1-hydroxy-2-methyl-2-(E)-butenyl -4-diphosphate synthase	*HDS1*	3-hydroxy-3-methylglutaryl-CoA synthase	*HMGS1*
	HDS2		*HMGS2*
3-hydroxy-3-methylglutaryl-CoA reductase	*HMGR1*		*HMGS3*
	HMGR2	Isopentenyl diphosphate isomerase	*IDI1*
	HMGR3		*IDI2*
	HMGR4	Isopentenyl mono-phosphate kinase	*IPK*

Supplementary Table 35. The information of TPSs in de novo transcriptome against the *L. cubeba* genome data.

TPSs ID in transcriptome *de novo* assembled	*LcuTPS* ID in *L. cubeba* genome	Identity %	Alignment length	e-value	Bit score
Cluster-18093.0	Lcu08G_22935	97.361	758	0	1282
Cluster-943.37864	Lcu09G_25125	99.102	891	0	1596
Cluster-26240.0	Lcu09G_25125	99.118	1020	0	1829
Cluster-46233.4	Lcu10G_27196	97.599	708	0	1214
Cluster-32088.1	Lcu08G_22935	98.225	1127	0	1964
Cluster-28069.0	Lcu03G_10509	99.701	1674	0	3064
Cluster-28069.2	Lcu03G_10509	99.558	1131	0	2061
Cluster-3526.0	Lcu10G_27145	99.863	1460	0	2684
Cluster-46233.1	Lcu10G_27196	97.442	1251	0	2134
Cluster-943.6206	Lcu04G_12301	99.55	666	0	1210
Cluster-13050.0	Lcu10G_27179	97.012	1506	0	2527
Cluster-43352.0	Lcu08G_22670	99.609	1533	0	2798
Cluster-12614.0	Lcu03G_10509	99.598	994	0	1814
Cluster-14370.0	Lcu10G_27145	99.945	1809	0	3336
Cluster-1895.0	Lcu10G_27179	99.535	1506	0	2737
Cluster-46233.2	Lcu05G_16176	99.52	1251	0	2278
Cluster-46233.3	Lcu05G_16176	98.241	1251	0	2189
Cluster-943.42809	Lcu10G_27191	100	902	0	1666

Supplementary Table 36. Primers used for qRT-PCR.

Primer name	Sequence (5' → 3')
LcuTPS19-qRT-F	GTCTATCCAGTGTTACATGTATGAAGC
LcuTPS19-qRT-R	TTGAAGAAAGGGAGTGAAGTAAACT
LcuTPS20-qRT-F	ATGAGGTTGCCAGAGGTGATGTTC
LcuTPS20-qRT-R	ATGAAGAAAGGGAGTGTTGTGAACT
LcuTPS22-qRT-F	TTTCTTTCCAACAATCTCTCGG
LcuTPS22-qRT-R	CTATTGGATTAACTACTTCCTTG
LcuTPS25-qRT-F	GGCGAGTGATCGATACATGA
LcuTPS25-qRT-R	CTGTGAGGGCACATTTGATG
LcuTPS26-qRT-F	CCGGGTTGCTTCCTCTTGAT
LcuTPS26-qRT-R	GTGAGTTCCCCACTGGGATG
LcuTPS42-qRT-F	GTTGTCCTCAGCGGCTTCTT
LcuTPS42-qRT-R	GCTTGGATCGAATGGAGCAT
LcuTPS19-F	ATGGCATTGCAATTGCTTACTC
LcuTPS19-R	CTACATAAACTTAAAGGGTTCAGC
LcuTPS20-F	ATGGCATTGCAATTGCTTACTC
LcuTPS20-R	CTACATAAACTTAAAGGGTTCAGCC
LcuTPS22-F	ATGGCATTGCATTTGCTTACTC
LcuTPS22-R	TACATAATATTGAAGGGTTCAGCTAG
LcuTPS25-F	ATGTCTCTTAATCTCGTCTTCCCAT
LcuTPS25-R	TTATACATTATTAATTGGTATGGGCTC
LcuTPS42-F	ATGTTGTCCTCAGCGGCTTCT
LcuTPS42-R	CTAGATTCTGAAAGTTCCTCT
LcuTPS42-pCAMBIA1300S-F	tcagcagtcgaagagcATGTTGTCCTCAGCGGCTTC
LcuTPS42-pCAMBIA1300S-R	ttagcgtgtgaagagcGATTCTGAAAGTTCCTCTG
LcuTPS42-pET28a-F	tcagcagtcgaagagcATGTTGTCCTCAGCGGCTTC
LcuTPS42-pET28a-R	ttagcgtgtgaagagcGATTCTGAAAGTTCCTCTG
LcuTPS19-pCAMBIA1300S-F	tcagcagtcgaagagcATGGCATTGCAATTGCTTAC
LcuTPS19-pCAMBIA1300S-R	ttagcgtgtgaagagcCATAAACTTAAAGGGTTCA
LcuTPS20-pCAMBIA1300S-F	tcagcagtcgaagagcATGGCATTGCAATTGCTTAC
LcuTPS20-pCAMBIA1300S-R	ttagcgtgtgaagagcCATAAACTTAAAGGGTTCA
LcuTPS22-pCAMBIA1300S-F	tcagcagtcgaagagcATGGCATTGCATTTGCTTAC
LcuTPS22-pCAMBIA1300S-R	ttagcgtgtgaagagcCATAATATTGAAGGGTTCA
LcuTPS22-pET28a-F	tcagcagtcgaagagcATGGCATTGCATTTGCTTAC
LcuTPS22-pET28a-R	ttagcgtgtgaagagcCATAATATTGAAGGGTTCA
LcuTPS25-pCAMBIA1300S-F	tcagcagtcgaagagcATGTCTCTTAATCTCGTCTT
LcuTPS25-pCAMBIA1300S-R	ttagcgtgtgaagagcTACATTATTAATTGGTATG
LcuTPS25-pET28a-F	tcagcagtcgaagagcATGTCTCTTAATCTCGTCTT
LcuTPS25-pET28a-R	ttagcgtgtgaagagcTACATTATTAATTGGTATG

Supplementary References

1. Lander, E. S. & Waterman, M. S. Genomic mapping by fingerprinting random clones: a mathematical analysis. *Genomics* 2, 231-239 (1988).
2. One Thousand Plant Transcriptomes Initiative. One thousand plant transcriptomes and the phylogenomics of green plants. *Nature* 574, 679-685 (2019).
3. Song, Y., *et al*. Evolutionary comparisons of the chloroplast genome in Lauraceae and insights into loss events in the Magnoliids. *Genome Biol. Evol.* 9, 2354-2364 (2017).
4. Chanderbali, A. S. & Renner, W. S. S. Phylogeny and historical biogeography of Lauraceae: evidence from the chloroplast and nuclear genomes. *Anna. Mou. Bot. Gard.* 88, 104-134 (2001).
5. Rohwer, J. G. Toward a phylogenetic classification of the Lauraceae: evidence from matK sequences. *Systematic Botany* 25, 60-71 (2000).
6. Mirarab, S. *et al*. ASTRAL: genome-scale coalescent-based species tree estimation. *Bioinformatics*, 30, i541-i548 (2014).
7. Chen, J. *et al*. An evolutionarily conserved gene, *FUWA*, plays a role in determining panicle architecture, grain shape and grain weight in rice. *Plant J.* 83, 427-38 (2015).
8. Brewer, P. B. *et al*. PETAL LOSS, a trihelix transcription factor gene, regulates perianth architecture in the *Arabidopsis* flower. *Development* 131, 4035-45 (2004).
9. Moreno-Hagelsieb, G. & Latimer, K. Choosing BLAST options for better detection of orthologs as reciprocal best hits. *Bioinformatic*s 24, 319-24 (2008).
10. Amborella Genome Project. The Amborella genome and the evolution of flowering plants. *Science* 342, 1241089 (2013).
11. Lee, J. & Lee, I. Regulation and function of SOC1, a flowering pathway integrator. *J. Exp. Bot.* 61, 2247-2254. (2010).
12. Saladie, M., Wright, L. P., Garcia-Mas, J., Rodriguez-Concepcion, M. & Phillips, M.A. The 2-C-methylerythritol 4-phosphate pathway in melon is regulated by specialized isoforms for the first and last steps. *J. Exp. Bot.* 65, 5077-5092 (2014).
13. Jadaun, J. S. *et al*. Over-expression of *DXS* gene enhances terpenoidal secondary metabolite accumulation in rose-scented geranium and *Withania somnifera*: active involvement of plastid isoprenogenic pathway in their biosynthesis. *Physiol. Plant.* 159, 381-400 (2017).
14. Stamatakis, A. RAxML version 8: a tool for phylogenetic analysis and post-analysis of large phylogenies. *Bioinformatics* 30, 1312-1313 (2014).
15. Bailey, T. L. *et al*. MEME SUITE: Tools for motif discovery and searching. *Nucleic Acids Res*. 37, W202-W208 (2009).
16. Li, X. *et al*. Transcription factor CitERF71 activates the terpene synthase gene CitTPS16 involved in the synthesis of E-geraniol in sweet orange fruit. *J. Exp. Bot.* 68, 4929-4938 (2017).
17. Trapnell, C. *et al*. Differential gene and transcript expression analysis of RNA-seq experiments with TopHat and Cufflinks. *Nat. Protocols* 7, 562-578 (2012).
18. Haas, B. J. *et al*. Improving the *Arabidopsis* genome annotation using maxi-mal transcript alignment assemblies. *Nucleic Acids Res.* 31, 5654-5666 (2003).
19. Stanke, M. & Waack, S. Gene prediction with a hidden Markov model and a new intron

submodel. *Bioinformatics* 19, 215-225 (2003).
20. Majoros, W. H., Pertea, M. & Salzberg, S. L. TigrScan and GlimmerHMM: two open source ab initio eukaryotic gene-finders. *Bioinformatics* 20, 2878-2879 (2004).
21. Korf, I. Gene finding in novel genomes. *BMC Bioinformatics* 5, 59-59 (2004).
22. Burge, C. B. Identification of Genes in Human Genomic DNA. Ph. D. Thesis, California (1997).
23. Parra, G., Blanco, E. & Guiģo, R. GeneID in *Drosophila*. *Genome Res*. 10, 391-393 (2000).
24. Slater, G. S. C. & Birney, E. Automated generation of heuristics for biological sequence comparison. BMC *Bioinformatics* 6, 31-31 (2005).
25. Haas, B. J. *et al*. Automated eukaryotic gene structure annotation using EVidenceModeler and the program to assemble spliced alignments. *Genome Biol.* 9, R7 (2008).
26. Lin, L. Y., Han, X. J., Chen, Y. C., Wu, Q. K. & Wang, Y. D. Identification of appropriate reference genes for normalizing transcript expression by quantitative real-time PCR in *Litsea cubeba*. *Mol. Genet. Genomics* 288, 727-737 (2013).
27. Goossens, A. It is easy to get huge candidate gene lists for plant metabolism now, but how to get beyond? *Mol. Plant* 8, 2-5 (2014).
28. Chen, F., Tholl, D., Bohlmann, J. & Pichersky. E. The family of terpene synthases in plants: a mid-size family of genes for specialized metabolism that is highly diversified throughout the kingdom. *Plant J.* 66, 212-229 (2011).
29. Yin J. *et al*. Co-expression of peppermint geranyl diphosphate synthase small subunit enhances monoterpene production in transgenic tobacco plants. *New Phytol.* 213, 1133-1144 (2017).
30. Chang, Y. T., Chu, F. H. Molecular cloning and characterization of monoterpene synthases from *Litsea cubeba* (Lour.) Persoon. *Tree Genet. Genomes* 7, 835-844 (2011).
31. Han, M. V., Thomas, G. W. C., Lugo-Martinez, J. & Hahn, M. W. Estimating gene gain and loss rates in the presence of error in genome assembly and annotation using CAFE 3. *Mol. Biol. Evol.* 30, 1987-1997 (2013).
32. Huelsenbeck, J. P. & Ronquist, F. MRBAYES: Bayesian inference of phylogenetic trees. *Bioinformatics* 17, 754-755 (2001).
33. Proost, S. *et al*. i-ADHoRe 3.0-fast and sensitive detection of genomic homology in extremely large data sets. *Nucleic Acids. Res.* 40, e11 (2012).
34. Fostier, J. *et al*. A greedy, graph-based algorithm for the alignment of multiple homologous gene lists. *Bioinformatics* 27, 749-756 (2011).
35. Zhang, L. S. *et al*. The water lily genome and the early evolution of flowering plants. *Nature* 577, 79-84 (2020).
36. Chaw, S. M. *et al*. Stout camphor tree genome fills gaps in understanding of flowering plant genome evolution. *Nat. Plants* 5, 63-73 (2019).
37. Liu, X. *et al*. The genome of medicinal plant *Macleaya cordata* provides new insights into benzylisoquinoline alkaloids metabolism. *Mol. Plant* 10, 975-989. (2017).
38. Leseberg, C. H. *et al*. Genome-wide analysis of the MADS-box gene family in *Populus trichocarpa*. *Gene* 378, 84-94 (2006).
39. Parenicova, L. *et al*. Molecular and phylogenetic analyses of the complete MADS-box transcription factor family in *Arabidopsis*: new openings to the MADS world. *Plant Cell*

15, 1538-1551 (2003).
40. Arora, R. *et al*. MADS-box gene family in rice: genome-wide identification, organization and expression profiling during reproductive development and stress. *BMC Genomics* 8, 242 (2007).
41. Zhang, G. *et al*. The Apostasia genome and the evolution of orchid. *Nature* 549, 379-383 (2017).
