# Supplementary material for: The Litsea genome and the evolution of the laurel family
Source: Nat Commun. 2020 Apr 3;11:1675. doi: 10.1038/s41467-020-15493-5 (PMC7125107; doi:10.1038/s41467-020-15493-5)
Supplement: Supplementary file 3 — Reporting Summary [file 41467_2020_15493_MOESM3_ESM.pdf]

nature research

Corresponding author(s): Zhong–Jian Liu

Last updated by author(s): Mar 9, 2020

Reporting Summary

Nature Research wishes to improve the reproducibility of the work that we publish. This form provides structure for consistency and transparency in reporting. For further information on Nature Research policies, see Authors & Referees and the Editorial Policy Checklist .

Statistics

For all statistical analyses, confirm that the following items are present in the figure legend, table legend, main text, or Methods section.

n/a	Confirmed	
☐	☒	The exact sample size (n) for each experimental group/condition, given as a discrete number and unit of measurement
☐	☒	A statement on whether measurements were taken from distinct samples or whether the same sample was measured repeatedly
☒	☐	The statistical test(s) used AND whether they are one- or two-sided<br>*Only common tests should be described solely by name; describe more complex techniques in the Methods section.*
☒	☐	A description of all covariates tested
☒	☐	A description of any assumptions or corrections, such as tests of normality and adjustment for multiple comparisons
☒	☐	A full description of the statistical parameters including central tendency (e.g. means) or other basic estimates (e.g. regression coefficient) AND variation (e.g. standard deviation) or associated estimates of uncertainty (e.g. confidence intervals)
☒	☐	For null hypothesis testing, the test statistic (e.g. F, t, r) with confidence intervals, effect sizes, degrees of freedom and P value noted<br>*Give P values as exact values whenever suitable.*
☐	☒	For Bayesian analysis, information on the choice of priors and Markov chain Monte Carlo settings
☒	☐	For hierarchical and complex designs, identification of the appropriate level for tests and full reporting of outcomes
☒	☐	Estimates of effect sizes (e.g. Cohen's d, Pearson's r), indicating how they were calculated

Our web collection on statistics for biologists contains articles on many of the points above.

Software and code

Policy information about availability of computer code

Data collection	FALCON (ver. 0.5.0), SMRT Link v5.0.0, BWA-mem v0.7.17, Pilon v1.21 and sspacelongreads PBJelly v15.8.24 were used in genome assembly. HiC data was assembled into super-scaffold using SAMtools and LACHESIS. BUSCO, BLASTN v2.3.0, Trinity v2.4.0 and BLAT v35 were used in genome assessment.
Data analysis	Tandem Repeats Finder v4.04, RepeatMasker, RepeatModeler v1.0.11, TBLASTN v2.3.0, BLASTP v2.3.0, InterProScan, tRNAscan-SE v1.3.1, BLASTN v2.3.0 and Piler were used in genome annotation. mclblastline pipeline (v10-201), MUSCLE (v3.8.31), PAML package (v4.4c), PhyML, i-ADHoRe (v3.0) were used for identification of whole-genome duplication. OrthoFinder v2.2.3, Clustal Omega v1.2.4, TrimAl v1.2, RAxML v8.2.11, ASTRAL v5.6.1, PAML MCMCTREE, BLASTP v2.3.0 and Café v4.0.1 were used in phylogenetic tree construction and phylogenomic dating. Trinity v2.4.0 was used for transcriptome assembly. HomBlocks-2017, SplitsTree4, Platanus 1.2.4, bwa0.7.17, bcftools0.1.19 and samtools0.1.19 were used chloroplast genome assembly.

For manuscripts utilizing custom algorithms or software that are central to the research but not yet described in published literature, software must be made available to editors/reviewers. We strongly encourage code deposition in a community repository (e.g. GitHub). See the Nature Research guidelines for submitting code & software for further information.

Data

Policy information about availability of data

All manuscripts must include a data availability statement. This statement should provide the following information, where applicable:

- Accession codes, unique identifiers, or web links for publicly available datasets
- A list of figures that have associated raw data
- A description of any restrictions on data availability

Provide your data availability statement here.

Field-specific reporting

Please select the one below that is the best fit for your research. If you are not sure, read the appropriate sections before making your selection.

☒ Life sciences ☐ Behavioural & social sciences ☐ Ecological, evolutionary & environmental sciences

For a reference copy of the document with all sections, see nature.com/documents/nr-reporting-summary-flat.pdf

Life sciences study design

All studies must disclose on these points even when the disclosure is negative.

Sample size	We produced genome data for Litsea cubeba, together with low-coverage genome data for 47 species of 20 genera and transcriptomic data for 23 species of 16 genera in Lauraceae. Furthermore, we downloaded the genome and transcriptome data for 36 representative plant species.
Data exclusions	No data exclusions in this manuscript
Replication	For the transcriptome sequencing data of flower buds and mix-tissues for 23 species of 16 genera in Lauraceae, three individuals of each species were used and the good reproducibility was confirmed.
Randomization	No randomization in this manuscript as samples were not allocated into experimental groups.
Blinding	No blinding in this manuscript as the data were not allocated into groups

Reporting for specific materials, systems and methods

We require information from authors about some types of materials, experimental systems and methods used in many studies. Here, indicate whether each material, system or method listed is relevant to your study. If you are not sure if a list item applies to your research, read the appropriate section before selecting a response.

Materials & experimental systems

n/a	Involved in the study	
☒	☐	Antibodies
☒	☐	Eukaryotic cell lines
☒	☐	Palaeontology
☒	☐	Animals and other organisms
☒	☐	Human research participants
☒	☐	Clinical data

Methods

n/a	Involved in the study	
☒	☐	ChIP-seq
☒	☐	Flow cytometry
☒	☐	MRI-based neuroimaging
