# Supplementary material for: The Litsea genome and the evolution of the laurel family
Source: Nat Commun. 2020 Apr 3;11:1675. doi: 10.1038/s41467-020-15493-5 (PMC7125107; doi:10.1038/s41467-020-15493-5)
Supplement: Supplementary file 2 — Peer Review [file 41467_2020_15493_MOESM2_ESM.pdf]

Reviewers' comments:

Reviewer #1 (Remarks to the Author):

Review of the Litsea genome and the evolution of the laurel family (Lauraceae).

This paper presents an excellent chromosome-level assembly of Litsea in the Lauraceae. The genome quality seems exceptional with BUSCO score of 93%.

The authors used phylogenetic methods to place Litsea with other Laurales taxa in a tree based on concatenated single-copy gene family alignments. The authors also remark that magnoliids are confidently placed as sister to eudicots. While alignment concatenation is certainly one valid approach to the problem, the understanding that incomplete lineage sorting (ILS) may play a strong role in confounding resolution of early branchings within angiosperms requires an attempt to use coalescent-based approaches that incorporate (e.g.) individual gene-tree information. This is perfectly straightforward to try, so the authors should also report results from a coalescent approach to "control for" confounding ILS. While the topology may well remain the same, that would in fact be a good and supportive finding for the paper.

Regarding "Gene families unique to L. cubeba were significantly enriched in the "biosynthesis of terpenoids and steroids" and "nitrogen metabolism" pathways, and the gene families that have 148 seen significant expansion were enriched in terms of "transferase activity," "catalytic activity," "protein phosphorylation," and "organic cyclic compound binding" in L. cubeba ... Unique or significantly expanded gene families in Lauraceae and L. cubeba were generally enriched in pathways related to the biosynthesis and accumulation of specific scents, such as monoterpenoid biosynthesis, plant hormone signal transduction, ABC transporters, and endocytosis"
- I think that statements like these require more than citation of GO terms or KEGG pathways; many genes of multitudinous functions wind up being annotated by interesting sounding features only to be less exciting when the actual genes or their homologs in Arabidopsis are looked at in detail. Please provide some examples of actual interesting genes, not just annotations, and I'd say for "scents" would be most important for the paper.

The authors' analyses of WGD events is extensive, and the incorporation of an approach to examine substitution rates was important. The discussion of the the possibility of 3 independent WGDs along short branches vis-a-vis rapid diversification in Laurales is important and should make its way into the abstract

In the section on phylogeny within Lauraceae, the network analysis has conclusions that are in my opinion overstated. There are a number of alternative interpretations of the reticulations observed, not only " multiple ancient hybridization events in the history of Lauraceae" as stated so strongly in the supplement. If the authors truly wish to assess the possibility of hybridization, they should use approaches that might reveal this for relatively recent admixtures - F statistics and TreeMix, both based on SNPs, are possibilities. Also possible is ADMIXTURE, based on SNPs. And the low-coverage reads the authors prepared for other purposes would be perfect for this. Networks are often incredibly overinterpreted as evidence for hybridization in the literature.

Why is the biogeographic analysis generally important for a NatComm reader? Not much is made from the findings other than provision of descriptive information. Also the PSMC analysis - why is this important, especially regarding possible bottleneck sharing with camphor??

The analyses of the FUWA and PETAL LOSS genes are very interesting and a nice adjunct to otherwise more standard genome-paper presentation. This should be of general interest to the botanical and evolution-deco community as well.

The terpene biosynthesis examples are also likely to be of interest to the secondary-metabolic community, and the analyses performed seem appropriate and extensive.

Finally, the Cassytha enhanced molecular evolutionary rate issue is of general interest for evolutionary biologists, even though a high-quality genome for it wasn't produced. As such, I think the observation could make its way into the abstract.

Reviewer #2 (Remarks to the Author):

In the manuscript “The Litsea genome and the evolution of the laurel family (Lauraceae)” Chen et al., build an array of resources to understand several important biological aspects of the laurel family. They generate a good draft genome of Listea cubeba, present a comprehensive WGD analysis, conduct an extensive phylogentic analysis of the Lauraceae using 47 species they sequenced, analyze the transcriptomes of unisexual and bisexual flowers and perform some preliminary follow up on important biosynthetic pathways. As the authors state in the first sentence of their conclusion, this will body of work will provide a valuable foundation for Lauraceae evolution. However, despite there being almost 150 pages of text, figures and tables, many of the methods were not explained sufficiently, sections read much more like a graduate thesis than a manuscript, and only some of the data described has been submitted to any repository. The data that was submitted to NCBI was not released (bioprojects could not be found), which makes it very hard to know what data was submitted (assemblies, raw data, gene models?). Moreover, as a reviewer it is important to have access to genome data and to understand the quality. Overall, there is a lot of great work in this manuscript. It either needs to be broken up into several manuscripts or the descriptions, findings, and results need to be more concise with direct links to specific methods used.

The premise of the manuscript is that the authors wanted to sequence Listsea cubeba to understand the evolutionary relationships within the Laurales and to address the debate about its phylogenetic position (lines 57-61). If this is the case then the authors should introduce the three recent genomes published on this topic in the introduction so that the reader has a broader context of what is known and how the current work either clarifies the debate or does not. The authors do utilize these genomes throughput the manuscript but at no point is there a discussion of what was found in those papers or how their interpretation either agrees or disagrees with those findings. The best parts of the manuscript are actually looking at the biosynthetic pathways or gene families that important. This is also only briefly mentioned in the introduction, so there is not much context some of this is in the supplemental notes and in the results/discussion but it would strengthen the manuscript to have more clearly articulated in the introduction.

It looks like the authors assembled the genome using falcon-unzip so the resulting assembly, which had a modest N50 considering the amount of PacBio sequence they generated, was still not haplotype resolved based on their BUSCO duplicate score. In general, this might not be a big deal since lots of genomes have been published that are not haplotype resolved, but the authors go on to do extensive gene family analysis. How does having almost 25% duplicate genes play a role in the analysis, overrepresented families etc? Also, it is interesting, and possibly suspect, that 10X actually increase the scaffold N50, but there is not much information on how this was done so it is very hard to evaluate. It is great that the authors went the next step and scaffolded the assembly into chromosomes using HiC. It is our experience that with that level of BUSCO duplication the HiC map would not look like ED figure 2. Did the authors look at collinearity with any other genomes to add support to the structure of the HiC scaffolded genome? If the genome had been available to the reviewer these may have been some of the quality checks tested.

It is unclear from the text and also the extensive supplements how the 47 low coverage genomes

were used. There is mapping information Supp Table 30, but were these mapped to L. cubeba? Was the chloroplast analysis on de novo assembled chloroplasts or mapped? These should be very clear in the main text and methods sections. For the transcriptome work, it seems that the analysis was conducted on assembled transcriptomes. Do we have any sense of what the quality of those transcriptome assemblies are? And then there are parts where the transcriptome data is used for expression analysis. Was this by mapping to the L. cubeba genome or done another way? Is the differential analysis provided anywhere in the text? Seems like the genes that are discussed are just cherry picked based on the literature-but it is possible they came out of an analysis that was not clear. The methods for the final validation experiments should be included in the main methods, even briefly so it is clear what was done. There are many questions like this throughout the manuscript where lots of works was done but it is not clear from the methods, figure legends or supplement how things were exactly carried out. Finally, the WGD section is very long covering lines 155 to 300 and several pages. The style of this section is not consistent with the rest of the manuscript and could benefit from some revisions to make it more concise.

Reviewer #3 (Remarks to the Author):

The manuscript by Chen and coworkers combines large-scale comparative genomics, transcriptomics and phylogenomics approaches to provide a deeper understanding of three major features of speciation within the family of Lauraceae, which are divergence of panicle and sexual differentiation, the phylogenetic relationships across the Lauraceae and related species, and the diversity of scent metabolism. The authors describe that several whole genome duplication events and expansion and likely divergence of key gene families have been major contributors to these speciation events. Gene expression and transient over-expression studies further suggest functions of selected genes identified in this study. The article is overall well written and presented. It provides expansive genome and transcriptome data and new genomic insights into the evolution of this relevant plant family.

However, in the opinion of this reviewer, the manuscript falls severely short of providing convincing data for the 'functional verification of key genes' as stated by the authors. Details regarding this major concern are listed below.

Firstly, the section on gene functional analyses is confusing to read, since it jumps from terpene synthases to ABA-insensitive genes, to transporters and again to terpene synthases without providing a clear argument why these candidate genes were chosen. For example, why were TPS22 and TPS42 chosen for analysis, but not TPS18-20, which are among the most abundant terpene synthase genes? Given the availability of robust assay systems for mono- and sesqui-terpene synthases, a more comprehensive functional study would greatly strengthen the arguments on scent metabolism made by the authors.

Furthermore, conclusions on gene functions are largely based solely on gene expression data, providing limited evidence for actual pathway involvement. In addition, prior studies on terpene synthase expression were not taken into consideration [e.g. Han et al. (2013) PlosOne 8(10):e76890]. For the in vitro functional characterization of TPS22no method is given, making it difficult to assess these results. For example, were the enzyme products verified by means of NMR, authentic standards or comparison to a reference database? Similarly, why was L. cubeba chosen as a system for transient over-expression rather than other available expression systems that prevent the issue of the endogenous monoterpene background in L. cubeba?

With respect to the discovery and annotation of terpene synthases, how was this achieved? For example, were terpene synthase candidate sequences identified in genome and transcriptome data curated for chimeric transcripts that often occur in this gene family given the high amino acid identity among terpene synthases? Additionally, beyond phylogeny-based annotation of TPSa, TPSb an diterpene synthases, were functional motifs (such as the mono-TPS RRX8W motif) and plastidial

transit peptides taken into account to solidify TPS family associations?

Related to these above comments, the authors state that the observed co-expression patterns of TPS42, DXS3, ABIC5 and ABCC5 show that these genes ‘are actively involved in monoterpene biosynthesis’. Mere co-expression is my no means sufficient evidence to proof pathway involvement. Hence, the statement on page 28, lines 499-501 along with similar statements on gene function verification throughout the manuscript should be carefully revised.

Other comments:

Page 4, line 51: The authors state that ‘the morphological adaptation in Lauraceae needs to be clarified’. While this reviewer agrees on this point, a justification based on knowledge in the field would be beneficial for a broader audience.

Page 24, line 435: At least a brief comment on what the major volatile components in Lauraceae are should be given, including the citation of relevant reference work.

Page 24, lines 439-440. The authors relate the expansion of the clade B DXS family with higher terpenoid accumulation in selected species. However, based on prior studies, it seems equally possible that the expansion of the DXS gene family enables the separation of DXS activities under different environmental stimuli, whereas changes in the gene expression may contribute to higher pathway productivity. This should be discussed.

Page 25, lines 451-452: Full amino acid length terpene synthases are listed here as ‘>200 bp’/ Do the authors mean amino acids? Even if so, mono- and sesqui-terpene synthases are typically ~550 amino acids long. As such including candidates far shorter than this will almost certainly overestimate the gene family size.

Page 26, line 473 & Page 28, line 496: The authors draw conclusions on the physiological roles of terpene synthases and their products merely based on their presence/absence or their gene expression levels in selected tissues without providing further data or support based on existing knowledge. As such, these conclusions are not sufficiently supported here.

Reviewer #1 (Remarks to the Author):

The authors used phylogenetic methods to place *Litsea* with other Laurales taxa in a tree based on concatenated single–copy gene family alignments. The authors also remark that magnoliids are confidently placed as sister to eudicots. While alignment concatenation is certainly one valid approach to the problem, the understanding that incomplete lineage sorting (ILS) may play a strong role in confounding resolution of early branchings within angiosperms requires an attempt to use coalescent–based approaches that incorporate (e.g.) individual gene–tree information. This is perfectly straightforward to try, so the authors should also report results from a coalescent approach to "control for" confounding ILS. While the topology may well remain the same, that would in fact be a good and supportive finding for the paper.

Response: We agree with the reviewer that ILS might indeed play an important role in confounding the resolution of early branches within angiosperms, and have therefore now also applied a coalescent–based approach that incorporates information from 160 individual gene–trees. The results of this analysis have been added to the new Figure 1 of the revised manuscript. We have also adapted the text in the Figure legend of Figure 1, and the text of the revised manuscript (lines 135 to 168).

Regarding "Gene families unique to L. cubeba were significantly enriched in the “biosynthesis of terpenoids and steroids” and “nitrogen metabolism” pathways, and the gene families that have 148 seen significant expansion were enriched in terms of “transferase activity,” “catalytic activity,” “protein phosphorylation,” and “organic cyclic compound binding” in L. cubeba ... Unique or significantly expanded gene families in Lauraceae and L. cubeba were generally enriched in pathways related to the biosynthesis and accumulation of specific scents, such as monoterpenoid biosynthesis, plant hormone signal transduction, ABC transporters, and endocytosis".– I think that statements like these require more than citation of GO terms or KEGG pathways; many genes of multitudinous functions wind up being annotated by interesting sounding features only to be less exciting when the actual genes or their homologs in Arabidopsis are looked at in detail. Please provide some examples of actual interesting genes, not just annotations, and I'd say for "scents" would be most important for the paper.

Response: As suggested by the reviewer, we have now provided and discussed some examples of ‘actual interesting genes’, including monoterpene synthases (TPS-b), ABSCISIC ACID–INSENSITIVE 5 (ABI5) and ABC transporter C family members in the revised version of our manuscript. It is interesting to notice that the TPS and ABC transporter members form gene clusters on chromosome 8 and 12 (**Supplementary Fig. 5**).

The authors' analyses of WGD events is extensive, and the incorporation of an approach to examine substitution rates was important. The discussion of the possibility of 3 independent WGDs along short branches vis–a–vis rapid diversification in Laurales is important and should make its way into the abstract.

Response: As suggested by the reviewer, in the revised version of our paper, we have now added a statement on the possibility of three independent WGDs to the abstract.

In the section on phylogeny within Lauraceae, the network analysis has conclusions that are in my opinion overstated. There are a number of alternative interpretations of the reticulations observed, not only " multiple ancient hybridization events in the history of Lauraceae" as stated so strongly in the supplement. If the authors truly wish to assess the possibility of hybridization, they should use approaches that might reveal this for relatively recent admixtures – F statistics and TreeMix, both based on SNPs, are possibilities. Also possible is ADMIXTURE, based on SNPs. And the low–coverage reads the authors prepared for other purposes would be perfect for this. Networks are often incredibly overinterpreted as evidence for hybridization in the literature.

Response: We agree with the reviewer that there might be alternative interpretations of the reticulations observed. The SplitTree4 software could only detect potentially inconsistent signals for our phylogeny. Because we found a topological difference between different markers, we obtained a method to estimate this discordance. TreeMix and ADMIXTURE are both well–known phylogenomic software packages and could provide robust evidence for potential introgression and hybridization. We therefore attempted to use these two methods on our SNPs data. However, we could not obtain enough shared SNPs for Lauraceae species. Although we increased the missing rate to as high as 75% for our Lauraceae species, we could only obtain 25,098 SNP loci, which would no doubt cause a loci bias. Thus, we did not use TreeMix and ADMIXTURE on our data for two reasons. 1) There was a too low coverage for the short reads data against our reference genome because Lauraceae is a family with high diversity (**Supplementary Table 25**), such as *Chimonanthus praecox* with mapping rate 26.64%, *Cryptocarya brachythyrsa* with mapping rate 21.27%, *Beilschmiedia percoriacea* with mapping rate 19.89%, *Cassytha filiformis* with mapping rate 10.66%. And the high missing rate for the SNP dataset would cause a false positive result. 2. We only had single individual to represent a species, and they also represented different genera or tribes. We did not have population or continuous sampling data, and therefore it was not possible to use ADMIXTURE. Instead, to describe the topological heterogeneity, we compared trees that were reconstructed by different molecular markers (the nuclear genes, and plastid genomes), and also used both MSC method and ASTRAL to identify possible phylogenetic conflict (see **Fig. 3 and Supplementary Figs. 11,12**) (lines 281 to 292).

Why is the biogeographic analysis generally important for a NatComm reader? Not much is made from the findings other than provision of descriptive information. Also the PSMC analysis – why is this important, especially regarding possible bottleneck sharing with camphor??

Response: We agree with the reviewer's comment and have now removed the biogeographic analysis and PSMC analysis from the revised version of our manuscript.

Finally, the Cassytha enhanced molecular evolutionary rate issue is of general interest for evolutionary biologists, even though a high–quality genome for it wasn't produced. As such, I think the observation could make its way into the abstract.

Response: We agree that the Cassytha enhanced molecular evolutionary rate issue is of general interest for evolutionary biologists, and we have now included this result in the abstract of our revised manuscript.

Reviewer #2 (Remarks to the Author):

In the manuscript "The *Litsea* genome and the evolution of the laurel family (Lauraceae)" Chen et al., build an array of resources to understand several important biological aspects of the laurel family. They generate a good draft genome of *Listea cubeba*, present a comprehensive WGD analysis, conduct an extensive phylogentic analysis of the Lauraceae using 47 species they sequenced, analyze the transcriptomes of unisexual and bisexual flowers and perform some preliminary follow up on important biosynthetic pathways. As the authors state in the first sentence of their conclusion, this will body of work will provide a valuable foundation for Lauraceae evolution. However, despite there being almost 150 pages of text, Figures and Tables, many of the methods were not explained sufficiently, sections read much more like a graduate thesis than a manuscript, and only some of the data described has been submitted to any repository. The data that was submitted to NCBI was not released (bioprojects could not be found), which makes it very hard to know what data was submitted (assemblies, raw data, gene models?). Moreover, as a reviewer it is important to have access to genome data and to understand the quality. Overall, there is a lot of great work in this manuscript. It either needs to be broken up into several manuscripts or the descriptions, findings, and results need to be more concise with direct links to specific methods used.

Response: We would like to thank the reviewer for his/her positive comments and helpful suggestions. To better explain the methods used, we have now provided more detailed explanations on "Low coverage genome sequencing and plastid genome assembly" (lines 778–793), "Transcriptomic data and analysis in Lauraceae" (lines 794–814) and "TPSs identification and functional validation experiments." (lines 847–878) in different subsections of the 'Methods'. To make the manuscript more clearly and concise, we have also revised the 'Results and discussion' section, as also suggested by reviewer 1. We have further revised the subsections "Genome sequencing and annotation" (lines 89–133), "Biosynthesis of the specific scent in Lauraceae" (lines 364–428), and shortened the "Whole genome duplications in Laurales"

subsection (lines 178 to 253), while removing "the Biogeography of Lauraceae" as suggested by reviewer 1.

All the data described and discussed in this manuscript, including whole genome assembly data (raw data, assemblies) (PRJNA562049), transcriptome data of 23 Lauraceae species (PRJNA562115) and low–coverage genome data of 47 Lauraceae species (PRJNA562080), have now been released to the NCBI.

The premise of the manuscript is that the authors wanted to sequence *Litsea cubeba* to understand the evolutionary relationships within the Laurales and to address the debate about its phylogenetic position (lines 57–61). If this is the case then the authors should introduce the three recent genomes published on this topic in the introduction so that the reader has a broader context of what is known and how the current work either clarifies the debate or does not. The authors do utilize these genomes throughput the manuscript but at no point is there a discussion of what was found in those papers or how their interpretation either agrees or disagrees with those findings. The best parts of the manuscript are actually looking at the biosynthetic pathways or gene families that important. This is also only briefly mentioned in the introduction, so there is not much context some of this is in the supplemental notes and in the results/discussion but it would strengthen the manuscript to have more clearly articulated in the introduction.

Response: To better understand the evolutionary relationships within the Laurales, we sequenced the *L. cubeba* genome and referred to the three recently published genomes of *Liriodendron chinense*, *Cinnamomum kanehirae* and *Persea americana* in the introduction. These different genomes were utilized in the phylogenetic analysis. According to the reviewer's suggestion, we have now provided a summary of the findings of these previous studies and their interpretation in terms of the phylogenetic position of magnoliids in the introduction (lines 63-75) and subsection "The Phylogenetic Relationships among Magnoliids, Eudicots, and Monocots" (lines 135–168).

It looks like the authors assembled the genome using falcon–unzip so the resulting assembly, which had a modest N50 considering the amount of PacBio sequence they generated, was still not haplotype resolved based on their BUSCO duplicate score. In general, this might not be a big deal since lots of genomes have been published that are not haplotype resolved, but the authors go on to do extensive gene family analysis. How does having almost 25% duplicate genes play a role in the analysis, overrepresented families etc?

Response: The high level of BUSCO duplicated genes was an intermediate result, which represented an assessment of the initial genome assembly rather than of the final genome. We apologize for not correcting the BUSCO results in our initial manuscript. We took steps to remove genome redundancy from our assembly, and these steps are described in our revised manuscript. We have now updated the BUSCO results, with the BUSCO results for the final version of the genome containing 6.3% duplicates (**Supplementary Table 5**). We have also added detailed descriptions of the assembly steps for heterozygous sequence removal in the of "Genome assembly and assessment" subsection (lines 582 to 623).

Also, it is interesting, and possibly suspect, that 10X actually increase the scaffold N50, but there is not much information on how this was done so it is very hard to evaluate.

Response: In the revised version of our manuscript, we have now added a description of the scaffolding process using 10X genomics data (lines 602 to 608).

It is great that the authors went the next step and scaffolded the assembly into chromosomes using HiC. It is our experience that with that level of BUSCO duplication the HiC map would not look like ED Figure 2. Did the authors look at collinearity with any other genomes to add support to the structure of the HiC scaffolded genome? If the genome had been available to the reviewer these may have been some of the quality checks tested.

Response: We apologize for the confusing description. The high level of BUSCO duplicated genes in our initial manuscript was an intermediate result (see also response higher), which represented an assessment of the initial genome assembly, and not the final version of the genome. We apologize for not correcting this supplementary Table in our initial version of the manuscript. As described in our revised manuscript, we conducted a genome redundancy filtering step before applying 10X genomics and Hi–C scaffolding. After filtering, the redundancy level of the genome was significantly reduced, with the proportion of BUSCO duplicated genes reduced to 6.3% (see **Supplementary Table 5**). To evaluate the Hi–C assembly, a chromosome-scale genome assembly of close relatives might be required. However, genome resources for Lauraceae are/were rare. The recently published *C. kanehirae* genome had a genome size of 730.7 Mb, which was significantly smaller than our genome, and the divergence time between *C. kanehirae* and *L. cubeba* was estimated to be 28 Mya; thus, several large-scale genome rearrangement events might have occurred between these two species. In this situation, we did not try to evaluate our assembly using the *C. kanehirae* genome.

It is unclear from the text and also the extensive supplements how the 47 low coverage genomes were used. There is mapping information Supp Table 30, but were these mapped to *L. cubeba*? Was the chloroplast analysis on de novo assembled chloroplasts or mapped? These should be very clear in the main text and methods sections.

Response: We have now provided a description to address this. Low coverage genome sequence data were generated for 47 species, including a 15x strategy for species in *Litsea* and 30 x strategy for species in other genera in Lauraceae (**Supplementary Table 24**). The chloroplast genome data were de novo assembled from the low coverage genomes sequencing data and used to construct a phylogenetic tree (**Supplementary Fig. 11**). To further assess the possibility of hybridization in Lauraceae, the low coverage genome sequencing data were mapped to *L. cubeba* and the mapping information was shown in **Supplementary Table 25**. We have included a description in the "Low coverage genome sequencing and plastid genome assembly" subsection (lines 778 to 793) of 'Methods'.

For the transcriptome work, it seems that the analysis was conducted on assembled transcriptomes. Do we have any sense of what the quality of those transcriptome assemblies are? And then there are parts where the transcriptome data is used for expression analysis. Was this by

mapping to the *L. cubeba* genome or done another way? Is the differential analysis provided anywhere in the text? Seems like the genes that are discussed are just cherry picked based on the literature–but it is possible they came out of an analysis that was not clear.

Response: We performed the assessment of the quality of the transcriptome assemblies using BUSCO (https://busco.ezlab.org/). A quality assessment of the mixed-tissue transcriptome assemblies for 23 species, representing 16 genera (**Supplementary Table 23**), indicated that the completeness was generally more than 80% (**Supplementary Figure 20**). The assessment results for the flower bud transcriptomes for 21 species, representing 13 genera (**Supplementary Table 26**), are shown in **Supplementary Figure 20**. Most transcriptome assemblies, except *Cinnamomum burmanni*–1,–2,–3 and *Cinnamomum verum*–3, were found to have a completeness close to or exceeding 80%. Therefore, we excluded the transcriptome data of *Cinnamomum burmanni*–1,–2,–3 and *Cinnamomum verum*–3 (**Supplementary Fig. 20**) when we analyzed the gene differentially expressed genes in male and female flower buds of Lauraceae. We have added the description of the transcriptome assembly and data analysis in the 'Method' section, titled "Transcriptomic data and analysis in Lauraceae" (lines 794–814).

The methods for the final validation experiments should be included in the main methods, even briefly so it is clear what was done. There are many questions like this throughout the manuscript where lots of works was done but it is not clear from the methods, Figure legends or supplement how things were exactly carried out.

Response: We have now added a "TPSs identification and functional validation experiments" (lines 847–878) subsection to the 'Method' section. To clearly explain how the work was undertaken, we have included "Low coverage genome sequencing and plastid genome assembly" (lines 778–793), "Transcriptomic sequencing and analysis in Lauraceae" (lines 794–814) subsections in the 'Method' section, as advised by the reviewer. We have also provided more detailed descriptions for "Genome assembly and assessment" (lines 582–623) and "Phylogenetic reconstruction" (lines 686–721) subsection in the 'Method' section and have referred to these descriptions in the figure legends.

Finally, the WGD section is very long covering lines 155 to 300 and several pages. The style of this section is not consistent with the rest of the manuscript and could benefit from some revisions to make it more concise.

Response: We have now shortened the WGD section, and have made it more concise "Whole genome duplications in Laurales" (lines 178 to 253).

Reviewer #3 (Remarks to the Author):

In the opinion of this reviewer, the manuscript falls severely short of providing convincing data for the 'functional verification of key genes' as stated by the authors. Details regarding this major

concern are listed below.

Firstly, the section on gene functional analyses is confusing to read, since it jumps from terpene synthases to ABA–insensitive genes, to transporters and again to terpene synthases without providing a clear argument why these candidate genes were chosen. For example, why were TPS22 and TPS42 chosen for analysis, but not TPS18–20, which are among the most abundant terpene synthase genes? Given the availability of robust assay systems for mono– and sesqui–terpene synthases, a more comprehensive functional study would greatly strengthen the arguments on scent metabolism made by the authors.

Response: To ensure that the section on gene functional analysis reads more smoothly, we have now reorganized the description in the "Biosynthesis of the specific scent in Lauraceae" subsection (lines 364–428). Although TPS18-20, 22, and 42 are among the most abundant terpene synthase genes, the corresponding TPS19-20 enzymes have been functionally characterized in vitro (Chang and Chu, 2011) and were not indicated to be potential mediators for the biosynthesis of the main components. With respect to the availability of the assay systems for mono– and sesqui–terpene synthases, we further conducted an experiment to establish the functional verification of TPS19 ,20, 22, 25 and 42 in tobacco according to the reviewer's comments (**Fig. 5, Supplementary Figs. 17,18, and Supplementary Note 12**).

Chang, Y.T. & Chu, F.H. Molecular cloning and characterization of monoterpene synthases from *Litsea cubeba* (Lour.) Persoon. Tree Genet. *Genomes* **7**, 835–844 (2011)

Furthermore, conclusions on gene functions are largely based solely on gene expression data, providing limited evidence for actual pathway involvement. In addition, prior studies on terpene synthase expression were not taken into consideration [e.g. Han et al. (2013) PlosOne 8(10):e76890].

Response: For the functional verification of genes involved in monoterpene biosynthesis, we conducted an endogenous and heterologous transient over–expression assay in vivo, and the in vitro functional characterization of TPS19 ,20, 22, 25 and 42. Therefore, we made conclusions not solely based on gene expression data, but more according to the functional verification. There is limited evidence for actual pathway involvement. We have revised the conclusions section in the updated manuscript. (lines 398-428). Also, in the revised manuscript, we have now referred to previous studies [e.g. Han et al. (2013) *PLoS ONE* **8**, e76890]

Han, X.J., Wang, Y.D., Chen, Y.C., Lin, L.Y., Wu, Q.K. Transcriptome sequencing and expression analysis of terpenoid biosynthesis genes in *Litsea cubeba*. *PLoS ONE* **8**, e76890 (2013)

For the in vitro functional characterization of TPS22 no method is given, making it difficult to assess these results. For example, were the enzyme products verified by means of NM R, authentic standards or comparison to a reference database?

Response: In the revised version of our manuscript, we have added a description of this method in

the “TPSs identification and functional validation experiments” (lines 847–878) subsection of the ‘Method’ section.

“The volatiles were analyzed using GC-MS. To identify the target monoterpene, the retention time was compared with that of an authentic standard purchased from Sigma-Aldrich, which was further validated using the NIST Mass Spectral Library.” (lines 874–876)

Similarly, why was *L. cubeba* chosen as a system for transient over–expression rather than other available expression systems that prevent the issue of the endogenous monoterpene background in *L. cubeba*?

Response: It is a good idea to choose a system that avoids the endogenous monoterpene background. We have now provided new data to address this. To avoid the issue of the endogenous monoterpene background, the heterologous transient over–expression of LcuTPS19, 20, 22, 25 and 42 was employed in tobacco (*Nicotiana tabacum*) leaves (**Fig. 5, Supplementary Note 12**). Tobacco produces almost no monoterpene, but it does produce diterpenoids (Yin et al., 2017), and therefore is an ideal system for monoterpene gene functional verification.

Yin, J.L., Wong, W.S., Jang, I.C., Chua, N.H. Co–expression of peppermint geranyl diphosphate synthase small subunit enhances monoterpene production in transgenic tobacco plants. *New Phytol*. **213**, 1133–1144 (2017)

With respect to the discovery and annotation of terpene synthases, how was this achieved? For example, were terpene synthase candidate sequences identified in genome and transcriptome data curated for chimeric transcripts that often occur in this gene family given the high amino acid identity among terpene synthases? Additionally, beyond phylogeny–based annotation of TPSa, TPSb an diterpene synthases, were functional motifs (such as the mono–TPS RRX8W motif) and plastidial transit peptides taken into account to solidify TPS family associations?

Response: With respect to the discovery and annotation of terpene synthases, we adopted the following procedure: “Candidate TPSs were scanned using pfamscan based on the HMMER suite (http://hmmer.janelia.org/) in the predicted proteome of *L. cubeba* and other species. The PF01397 and PF03936 model data were used as queries (e-value<10^{-5}). The putative full-length TPSs (>200 amino acids in length) were further inspected with InterProScan5 (http://www.ebi.ac.uk/interpro/scan.html). The full-length TPSs were analyzed with ChloroP for the prediction of N-terminal plastidial targeting peptides (http://www.cbs.dtu.dk/services/ChloroP/). An analysis of the exon/intron structures of the full-length TPS genes were also conducted using GSDS (http://gsds.cbi.pku.edu.cn/), and the conserved motif RRx(8)W and DDXXD were also labeled (**Supplementary Fig. 21**).” (lines 848– 855).

To determine whether chimeric transcripts existed for members of the gene family of terpene synthases, we conducted a local blast (E value < 1×10^{-6}) using 18 transcripts from the de novo assembled transcriptome of *L. cubeba* against the genome data to access the best hits. Finally, 18 transcripts were acquired that corresponded to the sequences in genome data (**Supplementary**

Table 35). Furthermore, we also conducted a local blast of all the TPSs from the transcriptome data of other species in Lauraceae in transcriptome data against the *L. cubeba* genome data, and found that all the TPSs are corresponded to the genome data. The results indicated there was no chimeric transcript error in the TPSs from our transcriptome data.

Related to these above comments, the authors state that the observed co–expression patterns of TPS42, DXS3, ABIC5 and ABCC5 show that these genes 'are actively involved in monoterpene biosynthesis'. Mere co–expression is my no means sufficient evidence to proof pathway involvement. Hence, the statement on page 28, lines 499–501 along with similar statements on gene function verification throughout the manuscript should be carefully revised.

Response: We agree with the reviewer's comments and have revised the statement. We deleted the sentence "Conclusively, the unique genes of Lauraceae, LcuABIC5 and LcuABCC5, co–expressed with the expanded genes LcuTPS42 and LcuDXS3 and are actively involved in monoterpene biosynthesis in the fruit of *L. cubeba*." in the revised version of our as well as removed similar statements regarding gene function verification throughout the manuscript.

Other comments:
Page 4, line 51: The authors state that 'the morphological adaptation in Lauraceae needs to be clarified'. While this reviewer agrees on this point, a justification based on knowledge in the field would be beneficial for a broader audience.

Response: We have now provided some additional text in the introduction regarding the current understanding of the morphological adaptation in Lauraceae (lines 56-60)

Page 24, line 435: At least a brief comment on what the major volatile components in Lauraceae are should be given, including the citation of relevant reference work.
Response: We have now included a brief statement regarding the detailed major volatile components in Lauraceae (lines 365–367).

Page 24, lines 439–440. The authors relate the expansion of the clade B DXS family with higher terpenoid accumulation in selected species. However, based on prior studies, it seems equally possible that the expansion of the DXS gene family enables the separation of DXS activities under different environmental stimuli, whereas changes in the gene expression may contribute to higher pathway productivity. This should be discussed.

Response: We agree with reviewer's point. We have now addressed this by adding the following text to the discussion: "It is possible that the gene duplication of TPS and DXS gene families lead to the separation of biological features in the terpenoid production (Chen et al., 2011; Saladie et al., 2014), and gene expression may enhance the production of main components in terpene." (lines 393–385).

Chen, F., Tholl, D., Bohlmann, J. & Pichersky. E. The family of terpene synthases in plants: a mid-size family of genes for specialized metabolism that is highly diversified throughout the kingdom.

Plant J. **66**, 212-229 (2011).
Saladie, M., Wright, L. P., Garcia-Mas, J., Rodriguez-Concepcion, M. & Phillips, M. A. The 2-C-methylerythritol 4-phosphate pathway in melon is regulated by specialized isoforms for the first and last steps. *J. Exp. Bot.* **65**, 5077–5092 (2014).

Page 25, lines 451–452: Full amino acid length terpene synthases are listed here as '>200 bp'/ Do the authors mean amino acids? Even if so, mono– and sesqui–terpene synthases are typically ~550 amino acids long. As such including candidates far shorter than this will almost certainly overestimate the gene family size.

Response: We agree with the reviewer's comments. We changed "200 bp" to "200 aa" in the statement "full amino acid length > 200 aa". Generally, in *L. chinense*, *C. kanehirae, L. cubeba,* and *P. americana* genome data, TPSs analysis is based on >200 aa. We selected TPSs > 200 aa long when comparing these genome data. We also learned that a TPS with 292 amino acids, CitTPS16, could be functional in biological process from the literature (Li et al., 2017). For example, CitTPS16 are involved in the synthesis of E–geraniol in sweet orange fruit (Li et al., 2017). To avoid missing any potential TPSs members, we finally used TPSs with full amino acid length > 200 aa for analysis. We also agree with the reviewer's comments that mono– and sesqui–terpene synthases are typically ~550 amino acids long. There are 41 LcuTPSs in *L. cubeba*. We took LcuTPSs with ~550 amino acids (LcuTPS19, 20, 22, 25, and 42) into consideration for gene functional verification.

Li, X., Xu, Y., Shen, S., Yin, X., Klee, H., Zhang, B., Chen., K. Transcription factor CitERF71 activates the terpene synthase gene CitTPS16 involved in the synthesis of E-geraniol in sweet orange fruit. *J. Exp. Bot.* **68**, 4929–4938 (2017)

Page 26, line 473 & Page 28, line 496: The authors draw conclusions on the physiological roles of terpene synthases and their products merely based on their presence/absence or their gene expression levels in selected tissues without providing further data or support based on existing knowledge. As such, these conclusions are not sufficiently supported here.

Response: Because these conclusions were not sufficiently supported, we have now deleted the statement.

Reviewers' comments:

Reviewer #1 (Remarks to the Author):

The majority of concerns I had have now been addressed in this revision. Regarding incomplete lineage sorting (ILS) and angiosperm phylogenetic relationships, it would be more complete to compare the authors new ASTRAL studies with the ILS investigations presented as part of the avocado genome project (Rendon-Anaya et al, 2019). There, the authors studied gene family turnover at internal branches for different resolutions of magnoliids, with the conclusion that magnoliids sister to eudicots+monocots was best supported by likelihood testing. Nonetheless, those authors presented the case that ILS may have generated a "biologically hard" problem that perhaps cannot be resolved using sequence data. Those authors presented a syntenic distance tree based on synonymous substitution peaks and neighbor-joining that again supported magnoliids sister to euciots+monocots. An incredibly simple approach subject to substantial error, to be sure, but probably also worth mentioning here, since the authors go to the trouble to note another synteny-based approach.... The present authors cite synteny network analyses done by some of them supporting monocots+magnoliids (also seen in a recent Biorxiv paper by the same authors)... but these were based on presence/absence data (presence/absence of syntenic cluster), and since loci can be inherited by hybridization (for example allopolyploidy), if I were the present authors, I would also note that phylogenies based on actual gene adjacencies (linked blocks) could provide the best overall answer. Last, some of the figures include Cinnamomum micranthum but the publication of that genome used the name Cinnamomum kanehirae.

Reviewer #2 (Remarks to the Author):

The authors have sufficiently addressed the concerns raised in the first submission. However, the WGD section is still too long in relation to the contribution to the overall manuscript.

Reviewer #3 (Remarks to the Author):

In this revised version of the manuscript, the authors largely addressed my concerns outlined for the initial submission of this article. I specifically, appreciate the authors effort in generating additional enzyme biochemical data that provide evidence for the role of individual terpene synthases in the biosynthesis of major scent components in L. cubeba. However, there are a few aspects that, in my view, require further revision ad discussion.

1) In the revised methods section, the authors state that they used full-length ORFs for in vitro activity assays of monoterpene synthases. Is this indeed correct? More commonly, monoterpene synthases are assayed as truncated enzymes lacking the plastidial signaling peptide and the authors do describe that they performed searches for plastid targeting peptides.

2) As per my prior concern, the authors used a cut-off of >200aa and an E-value of 10-5 to call full-length TPS genes. In my opinion, these values provide a low stringency threshold and hold the risk of over-counting the members of the gene family. While I do not insist on repeating the experiment with a more stringent threshold, the potential for the presence of partial or pseudogenes in the presented gene families should be discussed.

3) Also as per my prior comments, the authors observed a correlation of gene family size with increased essential oil amounts across the tested plants. This is very interesting, since the majority of published studies more typically show that differential regulation of TPS and more so key regulatory upstream pathway genes (such as DXS) control overall pathway productivity. This is also supported by the authors experiment in over-expressing the DXS3 gene. I believe the manuscript would benefit

from a more detailed discussion regarding the possible contribution of gene family size versus gene regulation (and likely a combination of both) on terpenoid accumulation.

Additional minor comments:

Line 123: A reference for the statement on the roles of TPS in scent metabolism in species of Lauraceae should be included.
Line 367: replace "volatile oils" with "essential oils"; replace "produced from Lauraceae" with "produced by Lauraceae"
Lien 369: Provide reference for the stated terpenoids produced in Lauraceae
Line 369: Replace "limited rating" with "rate-limiting"
Line 400: remove "the main"
Line 402: add "predicted as" after 27 …
Line 417: replace "the monoterpene was" with "monoterpenoids were"
Line 418: add "analysis" after GC-MS
Line 418: "[Asterisks indicate …]" I do not see any asterisks defining statistical values in the figure?
Line 420: Replace "GC-MS using authentic standards" with "GC-MS analysis as compared to authentic standards".
Line 440: Remove "expanded" before "TPS-b"
Line 862: Reference 46 cites a review on TPS functions rather
Line 872: Replace "vitro" with "in vitro"; replace "[there was …]" with "endogenous transient over-expression was performed in "

On the basis of these suggestions, I recommend the authors carefully review the manuscript to optimize language and formatting.

Reviewer #1 (Remarks to the Author):

The majority of concerns I had have now been addressed in this revision. Regarding incomplete lineage sorting (ILS) and angiosperm phylogenetic relationships, it would be more complete to compare the authors new ASTRAL studies with the ILS investigations presented as part of the avocado genome project (Rendon-Anaya et al, 2019). There, the authors studied gene family turnover at internal branches for different resolutions of magnoliids, with the conclusion that magnoliids sister to eudicots+monocots was best supported by likelihood testing. Nonetheless, those authors presented the case that ILS may have generated a "biologically hard" problem that perhaps cannot be resolved using sequence data. Those authors presented a syntenic distance tree based on synonymous substitution peaks and neighbor-joining that again supported magnoliids sister to euciots+monocots. An incredibly simple approach subject to substantial error, to be sure, but probably also worth mentioning here, since the authors go to the trouble to note another synteny-based approach.... The present authors cite synteny network analyses done by some of them supporting monocots+magnoliids (also seen in a recent Biorxiv paper by the same authors)... but these were based on presence/absence data (presence/absence of syntenic cluster), and since loci can be inherited by hybridization (for example allopolyploidy), if I were the present authors, I would also note that phylogenies based on actual gene adjacencies (linked blocks) could provide the best overall answer. Last, some of the figures include Cinnamomum micranthum but the publication of that genome used the name Cinnamomum kanehirae.

Response: *In the revised manuscript, we have included discussions about ILS and the gene turnover analyses performed in the avocado genome project. As suggested by the reviewer, we have now also proposed that synteny-based methods could be alternative approaches to solve the phylogenetic position of magnoliids, mentioning both the synteny-based methods used in the avocado genome project and the work from some of us published on Biorxiv and currently under review (lines 152 to 163). The name Cinnamomum kanehirae has been corrected.*

Reviewer #2 (Remarks to the Author):

The authors have sufficiently addressed the concerns raised in the first submission. However, the WGD section is still too long in relation to the contribution to the overall manuscript.

Response: *We have considerably (by about 25%) shortened the section on ‘whole genome duplications in Laurales’, as requested by the reviewer. We have reduced Fig. 2 and moved part of Fig. 2 to the supplementary (new supplementary Figure 8), as well as part of the text describing whole genome duplication analysis (Supplementary Note 2).*

Reviewer #3 (Remarks to the Author):

In this revised version of the manuscript, the authors largely addressed my concerns outlined for the initial submission of this article. I specifically, appreciate the authors effort in generating additional enzyme biochemical data that provide evidence for the role of individual terpene synthases in the biosynthesis of major scent components in L. cubeba. However, there are a few aspects that, in my view, require further revision ad discussion.

1) In the revised methods section, the authors state that they used full-length ORFs for in vitro activity assays of monoterpene synthases. Is this indeed correct? More commonly, monoterpene synthases are assayed as truncated enzymes lacking the plastidial signaling peptide and the authors do describe that they performed searches for plastid targeting peptides.

Response: *We have chosen TPS22, 25, and 42, three highly expressed TPS genes in the biosynthesis of essential oil, for the in vitro activity assays. According to the searching results for plastid targeting peptides, there were no plastid targeting peptides for TPS25 and 42. Therefore, we performed in vitro activity assays with the full-length ORFs of the two TPS genes. For TPS22, although it has the plastid targeting peptides, the expressed and purified protein based on the full-length ORFs, did not form inclusion bodies.*

2) As per my prior concern, the authors used a cut-off of >200aa and an E-value of 10-5 to call full-length TPS genes. In my opinion, these values provide a low stringency threshold and hold the risk of over-counting the members of the gene family. While I do not insist on repeating the experiment with a more stringent threshold, the potential for the presence of partial or pseudogenes in the presented gene families should be discussed.

Response: *In the revised version of our manuscript, we added a sentence to claim the possibility of including partial and/or pseudogenes (lines 687 to 693).*

3) Also as per my prior comments, the authors observed a correlation of gene family size with increased essential oil amounts across the tested plants. This is very interesting, since the majority of published studies more typically show that differential regulation of TPS and more so key regulatory upstream pathway genes (such as DXS) control overall pathway productivity. This is also supported by the authors experiment in over-expressing the DXS3 gene. I believe the manuscript would benefit from a more detailed discussion regarding the possible contribution of gene family size versus gene regulation (and likely a combination of both) on terpenoid accumulation.

Response: *Thanks for the suggestion. In the revised version of our manuscript, we have added a discussion of the possible contribution of gene family size and gene regulation on terpenoid accumulation in L. cubeba (lines 378 to 386).*

Additional minor comments:

Line 123: A reference for the statement on the roles of TPS in scent metabolism in species of Lauraceae should be included.
***Response:** we added a reference.*

Line 367: replace "volatile oils" with "essential oils"; replace "produced from Lauraceae" with "produced by Lauraceae"
***Response:** We revised the words according to the reviewer's suggestions.*

Lien 369: Provide reference for the stated terpenoids produced in Lauraceae
***Response:** we added a reference.*

Line 369: Replace "limited rating" with "rate-limiting"
***Response:** Done.*

Line 400: remove "the main"
***Response:** Done.*

Line 402: add "predicted as" after 27 …
***Response:** Done.*

Line 417: replace "the monoterpene was" with "monoterpenoids were"
***Response:** Done.*

Line 418: add "analysis" after GC-MS
***Response:** Done.*

Line 418: "[Asterisks indicate …]" I do not see any asterisks defining statistical values in the figure?
***Response:** We deleted the sentence. The significant differences were not analyzed because no monoterpenes were detected in the control.*

Line 420: Replace "GC-MS using authentic standards" with "GC-MS analysis as compared to authentic standards".
***Response:** Done.*

Line 440: Remove "expanded" before "TPS-b"
***Response:** Done.*

Line 862: Reference 46 cites a review on TPS functions rather
***Response:** we cited a review on TPS functions here.*

Line 872: Replace “vitro” with “in vitro”; replace “[there was …]” with “endogenous transient over-expression was performed in “

Response: *Done.*

On the basis of these suggestions, I recommend the authors carefully review the manuscript to optimize language and formatting.

Response: *We carefully revised the manuscript so that it could comply with the format requirements of Nature Communications, and we have also further improved the language in the manuscript.*

REVIEWERS' COMMENTS:

Reviewer #1 (Remarks to the Author):

The authors have considered my last coments and incorporated them, though I disagree with their definition of incomplete lineage sorting (ILS):

"Although ILS is a result of nucleotide polymorphisms in the ancestral populations, prevalent copy number variations could also exist in the ancestral populations and exacerbate the effects of ILS, as suggested by gene count data used to infer the phylogenetic position of magnoliids in a previous study on the P. americana genome"

I think ILS is better understood as a problem of ancestral polymorphism not sorting according to the species tree, and this polymorphism is in terms of haplotypes or alleles - not necessarily via SNPs.. Structural variants ("copy number variations") can also help define haplotypic or allelic states. Therefore, copy number variants (e.g., tandem duplicates) can contribute to ILS directly (different numbers of tandems in arrays not sorting according to the species tree), as well as by differential extinction of (paralogous) copies leaving genealogies based on orthology difficult to discern from those based on orthology plus paralogy.

Anyway, a minor point for the paper itself ;) ;) but an important one I think when considering the role ILS can play in obscuring ancient branchings among angiosperm groups.

Reviewer #3 (Remarks to the Author):

The authors have satisfactorily addressed my comments regarding the prior version of this manuscript.

REVIEWERS' COMMENTS:

Reviewer #1 (Remarks to the Author):

The authors have considered my last comments and incorporated them, though I disagree with their definition of incomplete lineage sorting (ILS):

"Although ILS is a result of nucleotide polymorphisms in the ancestral populations, prevalent copy number variations could also exist in the ancestral populations and exacerbate the effects of ILS, as suggested by gene count data used to infer the phylogenetic position of magnoliids in a previous study on the *P. americana* genome"

I think ILS is better understood as a problem of ancestral polymorphism not sorting according to the species tree, and this polymorphism is in terms of haplotypes or alleles - not necessarily via SNPs.. Structural variants ("copy number variations") can also help define haplotypic or allelic states. Therefore, copy number variants (e.g., tandem duplicates) can contribute to ILS directly (different numbers of tandems in arrays not sorting according to the species tree), as well as by differential extinction of (paralogous) copies leaving genealogies based on orthology difficult to discern from those based on orthology plus paralogy.

Anyway, a minor point for the paper itself ;) ;) but an important one I think when considering the role ILS can play in obscuring ancient branchings among angiosperm groups.
Response: We revised the comments according to the reviewer’s comments. Please see MS p21, 500-502 lines.
